# Synthesis of New Triazolopyrazine Antimalarial Compounds

**DOI:** 10.3390/molecules26092421

**Published:** 2021-04-21

**Authors:** Daniel J. G. Johnson, Ian D. Jenkins, Cohan Huxley, Mark J. Coster, Kah Yean Lum, Jonathan M. White, Vicky M. Avery, Rohan A. Davis

**Affiliations:** 1 Griffith Institute for Drug Discovery, School of Environment and Science, Griffith University, Brisbane, QLD 4111, Australia; d.johnson@griffithuni.edu.au (D.J.G.J.); i.jenkins@griffith.edu.au (I.D.J.); c.huxley@griffithuni.edu.au (C.H.); m.coster@griffith.edu.au (M.J.C.); k.lum@griffith.edu.au (K.Y.L.); v.avery@griffith.edu.au (V.M.A.); 2 NatureBank, Griffith University, Brisbane, QLD 4111, Australia; 3 School of Chemistry and Bio21 Institute, The University of Melbourne, Melbourne, VIC 3010, Australia; whitejm@unimelb.edu.au; 4 Discovery Biology, Griffith University, Brisbane, QLD 4111, Australia

**Keywords:** Open Source Malaria, drug discovery, synthesis, triazolopyrazine, late-stage functionalization, photoredox, Diversinate^™^, methylation, difluoroethylation, antimalarial, X-ray, radical disproportionation, mechanism

## Abstract

A radical approach to late-stage functionalization using photoredox and Diversinate^™^ chemistry on the Open Source Malaria (OSM) triazolopyrazine scaffold (Series 4) resulted in the synthesis of 12 new analogues, which were characterized by NMR, UV, and MS data analysis. The structures of four triazolopyrazines were confirmed by X-ray crystal structure analysis. Several minor and unexpected side products were generated during these studies, including two resulting from a possible disproportionation reaction. All compounds were tested for their ability to inhibit the growth of the malaria parasite *Plasmodium falciparum* (3D7 and Dd2 strains) and for cytotoxicity against a human embryonic kidney (HEK293) cell line. Moderate antimalarial activity was observed for some of the compounds, with IC_50_ values ranging from 0.3 to >20 µM; none of the compounds displayed any toxicity against HEK293 at 80 µM.

## 1. Introduction

The 1,2,4-triazolo[4,3-a]pyrazine scaffold has been shown to display a variety of biological activities of importance to drug discovery and development, as well as chemical biology research. Examples of bioactivity associated with this electron-deficient and nitrogen-rich heterocyclic scaffold include compounds that bind to the *N*-methyl-D-aspartate subtype 2B receptor, which plays an important role in neurological disease states [1], patented inhibitors of renal outer medullary potassium channels [2], compounds that inhibit kidney urea transport [3], and bromodomain inhibitors, which may have implications for future cancer therapies [4].

**Figure 1:**
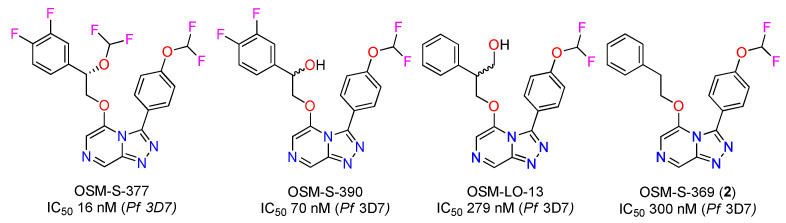
Some analogues under investigation by the OSM, including OSM-S-369, which was further investigated in this work [6].

For the past few years, the Open Source Malaria (OSM) consortium [5] has had a growing interest in the 1,2,4-triazolo[4,3-*a*]pyrazine scaffold, which has been designated Series 4 [6]. This compound series is believed to have several advantages over the previous series that have been or are being investigated within the OSM. In vitro testing has revealed human liver microsomal and hepatocyte stability, as well as hepatic intrinsic clearance of <8.1 µL/min/mg [6]. The series has demonstrated potency down to 0.016 µM against the malaria parasite *Plasmodium falciparum* (*Pf*) and appears to have little poly-pharmacology or cytotoxicity, giving confidence in its specificity and tolerability, making it ideal for a targeted medication [6]. A number of the more potent triazolopyrazine compounds identified to date are shown below in Figure 1, along with in vitro IC_50_ values. Through OSM investigations into the mechanism of action of the Series 4 compounds, it has been suggested that these compounds inhibit the ATPase, *Pf*ATP4 [7]. The inhibition of *Pf*ATP4 has been investigated with a number of other antimalarial compounds, including compounds based on 4-cyano-3-methylisoquinolines [8], and pyrazoloamides [9]. *Pf*ATP4 is considered to function as a Na^+^/H^+^-ATPase, which allows the malaria parasite to regulate Na^+^ to maintain cell homeostasis [9,10,11]. It was proposed that Series 4 compounds have the ability to interfere with this process, which means the parasite is unable to regulate Na^+^ [7]. The disruption of Na^+^ regulation results in a significant increase in the acid load of the cell, which can lead to parasite growth inhibition and ultimately parasite death [9,10,11].

A current aim for the Series 4 lead optimization involves improving solubility and metabolic stability, while maintaining potency [6]. Late-stage functionalization (LSF), incorporating small incremental modifications, has been employed to achieve this [4]. This strategy involves using C-H bonds as chemical handles for functionalization, bypassing the need for de novo synthetic strategies that may be costly and time consuming.

Herein we report the synthesis of several new triazolopyrazine derivatives employing LSF radical chemistry, in particular, photoredox methylations and Diversinate^™^ transformations. In vitro antimalarial activity and cytotoxicity are also reported for all analogues generated during these studies.

## 2. Results and Discussion

### 2.1. Synthesis of OSM-Based Scaffolds via Coupling of Primary Alcohols with Chloro-Heterocycles

**Scheme 1:**
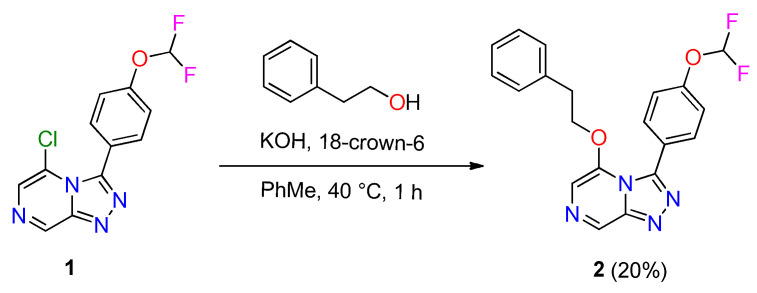
Synthesis of compound **2**.

Compound **1**, which was used in this project as a scaffold for semisynthesis and late-stage functionalization, has been previously synthesized and reported by the OSM project [12]. Initially, scaffold **1** was converted into a series of ether-substituted triazolopyrazine compounds, using a procedure (Scheme 1) based on that reported by Tse [13].

Two of the ether triazolopyrazine derivatives (compounds **2** and **3**) were known OSM compounds. Compound **2**, previously reported to display potent activity against *Pf* 3D7 with an IC_50_ value of 0.301 µM [14], was synthesized as the starting point for the proposed photoredox catalysis reactions and LSF Diversinate^™^ chemistry. Compound **1** was treated with 2-phenylethanol, potassium hydroxide (KOH), and 18-crown-6, dissolved in toluene (PhMe), and then briefly heated (Scheme 1). The reaction products were subsequently purified by silica flash chromatography using *n*-hexane/EtOAc stepwise gradient to afford pure **2**. No reaction optimization was undertaken since a sufficient quantity of the triazolopyrazine derivative **2** was obtained for the planned LSF chemistry.

**Scheme 2:**
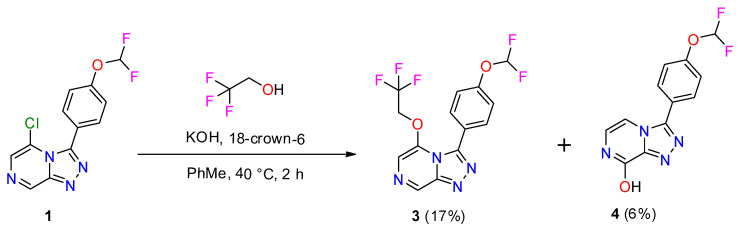
Synthesis of compound **3** and side product **4**.

**Scheme 3:**
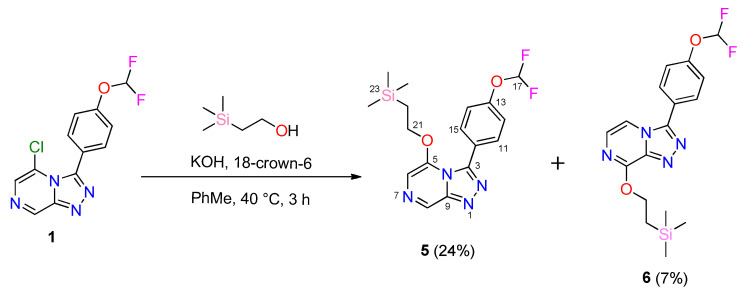
Synthesis of compounds 5 and 6.

Compound **3**, a previously reported OSM compound with low *Pf* activity (IC_50_ = 10 µM), was part of the inherited data set reported in the OSM master compounds’ list [15]. It was synthesized as an alternative starting point for the proposed photoredox catalysis reactions and LSF Diversinate^™^ chemistry. The synthesis of this compound was accompanied by the formation of an unexpected and unusual side product **4** (Scheme 2). The mechanism of formation of **4** is presumably analogous to that for the formation of **6** (Scheme 3) except that the attacking nucleophile is hydroxide rather than alkoxide. The strong inductive effect exerted by the three ß-fluorines of the 2,2,2-trifluoroethanol would decrease the nucleophilicity of the corresponding alkoxide, rendering hydroxide a competitive nucleophile.

**Figure 2:**
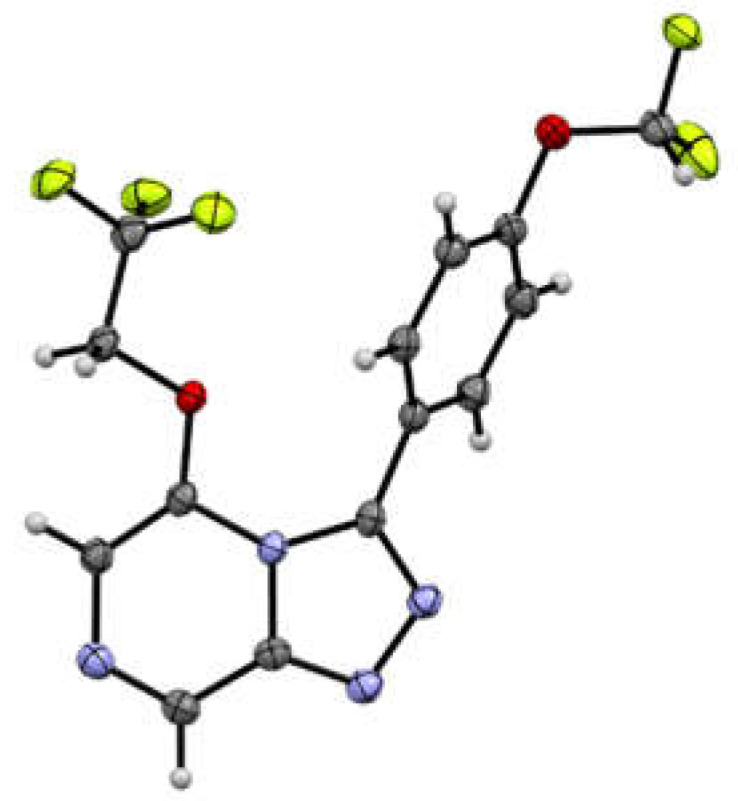
ORTEP drawing of compound **3**.

Crystals of compound **3** were successfully analyzed via X-ray crystallography, confirming the structure assignment (Figure 2). This is the first reported crystal structure of an ether-substituted triazolopyrazine.

The novel compound **5** was also synthesized using the commercially available primary alcohol, 2-(trimethylsilyl)ethanol. A triazolopyrazine derivative containing silicon that was chosen as the OSM project had not previously investigated any silyl analogues. While silicon-based drugs are rare, some examples of drug-like candidates containing silicon, which are bioavailable via an oral route of administration, are known [16].

Compound **5** was produced using the previously described method, except that the reaction time was increased to 3 h (Scheme 3). The synthesis of compound **5** was accompanied by the formation of another unusual side product (**6**). A proposed mechanism for how the *tele*-substitution product **6** is formed was recently published by Todd et al. [17].

The structures of all synthesized ethers and side products were determined using 1D/2D NMR and HRMS (see Supplementary Materials). An example of the full characterization for the new compound **5** is given below. Compound **5** was isolated as brown crystals and was assigned the molecular formula C_17_H_20_F_2_N_4_O_2_Si following analysis of HRESIMS ion at *m/z* 379.1396 [M+H]^+^ (calcd for C_17_H_21_F_2_N_4_O_2_Si, 379.1396). The ^1^H NMR spectrum in DMSO-*d*_6_ for **5** (Table 1) indicated the presence of two methylenes [δ_H_ 0.91 (H-22) and 4.33 (H-21)] and four aromatic protons [δ_H_ 7.81 (H-11 and H-15) and 7.31 (H-12 and H-14)]. The ^2^*J*_HF_ splitting of a triplet (73.6 Hz) was used to identify the proton located in the difluoromethoxy group [δ_H_ 7.36 (H-17)], and a strong singlet signal was used to identify the trimethylsilyl group [δ_H_ 0.07 (H-24, H-25 and H-26). The ^13^C NMR spectrum and edited HSQC spectrum indicated the presence of 17 carbons, including three methyls, two methylenes, six aromatic methines, one fluorinated methine, and five non-protonated sp^2^ carbons.

**Figure 3:**
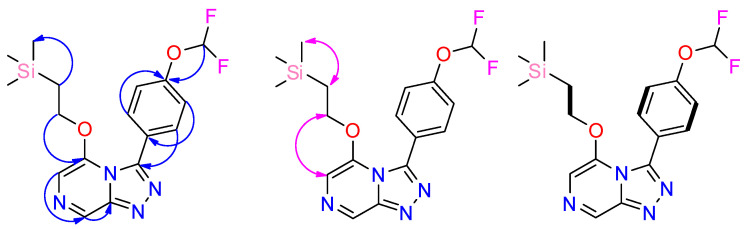
Key HMBC (→), ROESY (↔), and COSY (**—**) correlations for **5**.

**Figure 4:**
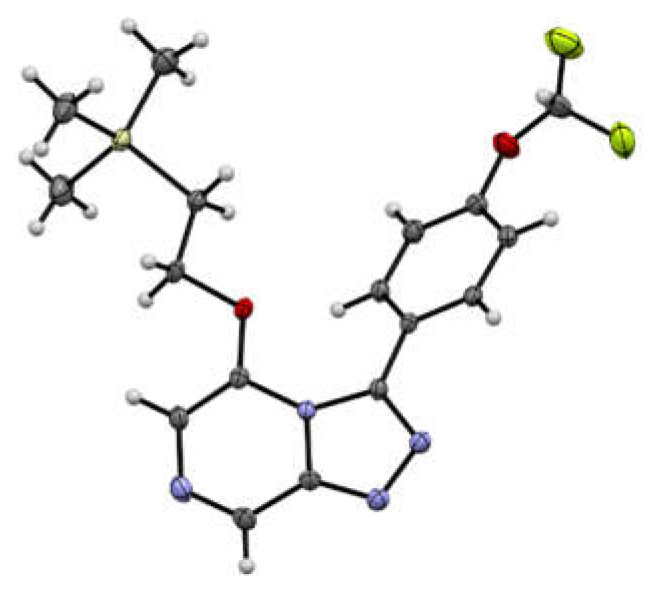
ORTEP drawing of compound **5**.

Analysis of the COSY system was able to corroborate the relationship of the doublet signals in the aromatic ring as being a part of the same spin system (H-11, H-12, H-14, and H-15). Analysis of the ^13^C NMR and HMBC correlations from the difluoromethoxy proton along with ^3^*J*_C-F_ splitting (3.3 Hz) allowed for the unambiguous assignment of C-13 (δ_C_ 151.9). This then allowed for definitive NMR assignments of the rest of the aromatic ring. Of note, HMBC correlations for H-11 and H-15 were identified to δ_C_ 151.9. Furthermore, another strong HMBC correlation from both H-11 and H-15 facilitated the assignment of C-3 (δ_C_ 145.6) on the triazolo ring. The coupling between the methylene protons δ_H_ 4.33 (H-21) and δ_H_ 0.91 (H-22) observed in COSY spectrum, together with a key ROESY correlation from δ_H_ 4.33 to δ_H_ 7.59 (H-6) and the HBMC correlations from the δ_H_ 4.33 to δ_C_ 143.9, supported the addition of the ether sidechain to the triazolopyrazine core at C-5. The HMBC correlation between the methylene H-22 to the trimethylsilyl group carbons C-24, C-25, and C-26 (δ_C_ -1.7) further confirmed the presence of trimethylsilyl ether. Key COSY, HMBC, and ROESY correlations for compound **5** are shown in Figure 3. These data enabled the chemical structure of **5** to be assigned. Furthermore, crystals obtained for **5** following silica flash chromatography and subsequent X-ray crystallography studies confirmed the NMR-based structure assignment. The ORTEP drawing of **5** is shown below in Figure 4.

### 2.2. Late-Stage Functionalization: Methylation of Series 4 Scaffolds and Ether Derivatives via Photoredox Catalysis

**Scheme 4:**
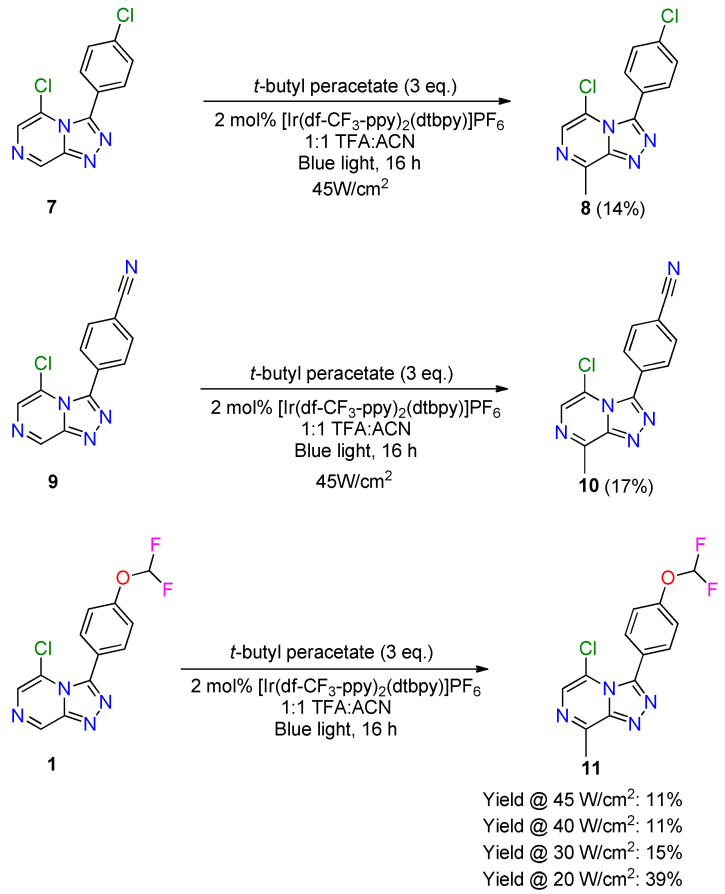
Synthesis of **8**, **10,** and **11** via photoredox-catalyzed methylation (for structure of the iridium catalyst, see Ref. [16]).

The first LSF reactions utilizing photoredox catalysis involved the use of a linear reflector made by Kessil^®^, which allowed for control over the intensity of the blue light (456 nm) and reaction vessel distance from the light source. The scaffolds **1**, **7**, and **9**, were initially used to test the methylation photoredox chemistry, based on methodology reported by DiRocco et al. [18]. Reactions were carried out using the scaffold substrate (0.1 mmol), *t*-butyl peracetate (3 eq.), iridium catalyst [Ir(dF-CF_3_-ppy)_2_(dtbpy)]PF_6_] (2 mol%). The nitrogen-sparged 1:1 TFA/ACN reaction mixture was stirred for 16 h while being irradiated with blue light from the Kessil^®^ photoreactor. Purification of the crude reaction products was carried out using reversed phase C_18_ HPLC (MeOH/H_2_O/0.1% TFA). Products and yields are reported in Scheme 4 below.

It was found that the yield of compound **11** could be significantly improved by reducing the light intensity from 45 to 20 W/cm^2^ (Scheme 4). Compounds **8**, **10,** and **11** were fully characterized using 1D/2D NMR and HRMS in order to confirm the methylation site on this heterocyclic scaffold.

**Scheme 5:**
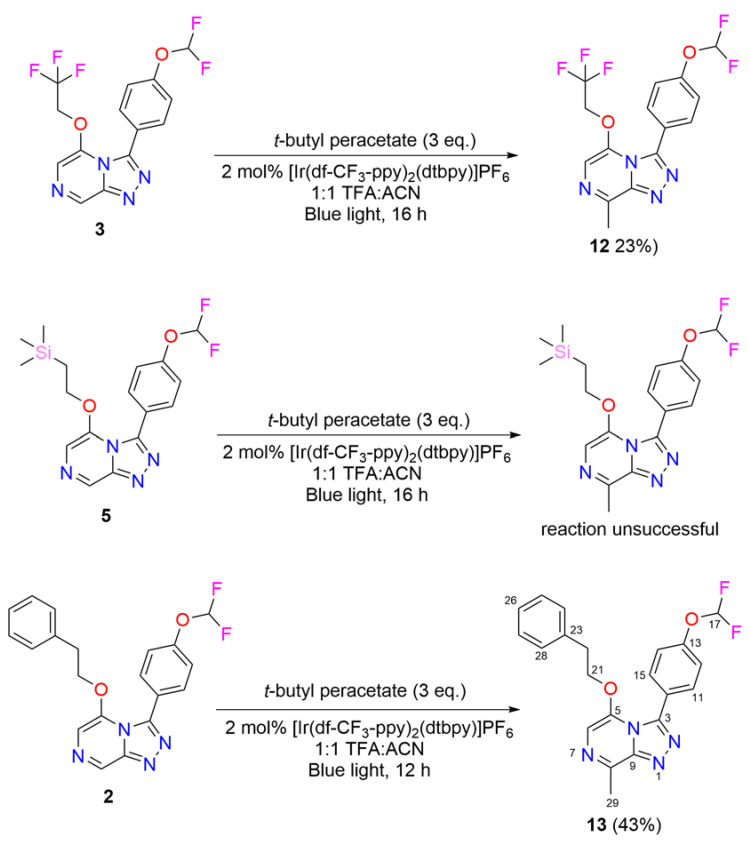
Photoredox-catalyzed methylation of the scaffolds **2**, **3,** and **5**.

Using these optimized reaction conditions described above, scaffold **3** was converted into the methylated derivative **12** in 23% yield (Scheme 5). However, using the same reaction conditions with scaffold **5** did not produce any methylated product. As all of the starting material had been consumed (as determined by LCMS), either **5** (or methylated **5**) must have been degraded, possibly via photodegradation, a noted criticism of photoredox synthesis in the literature [19]. Taking this into account with the methylation reaction of **2**, the reaction time was reduced from 16 to 12 h, resulting in the methylated product **13** in 43% yield (Scheme 5).

The structures of all synthesized methylated products were determined using 1D/2D NMR and HRMS (see Supplementary Materials). An example of full characterization for **13** is given below. Compound **13** was purified as a clear film and was assigned the molecular formula C_21_H_19_F_2_N_4_O_2_ following analysis of HRESMS ion at *m/z* 397.1470 [M + H]^+^. The ^1^H NMR spectrum of **13** in DMSO-*d*_6_ (Table 2) indicated the presence of two methylenes [δ_H_ 2.86 (H-22) and 4.42 (H-21)], 10 aromatic protons [δ_H_ 7.39 (H-6), δ_H_ 7.74 (H-11 and H-15), 7.29 (H-12 and H-14), δ_H_ 6.90 (H-24 and H-28), 7.17 (H-25 and H-27), 7.16 (H-26)], and a methyl singlet [δ_H_ 2.72 (H-29)]. The ^1^*J*_H-F_ splitting of a triplet (73.7 Hz) was used to identify the proton located in the difluoromethoxy group [δ_H_ 7.36 (H-17)]. Analysis of the COSY system allowed for two aromatic systems to be distinguished, with four aromatic protons [δ_H_ 7.74 (H-11 and H-15) and 7.29 (H-12 and H-14)] belonging to one system and five aromatic protons [δ_H_ 6.90 (H-24 and H-28), 7.17 (H-25 and H-27), and 7.16 (H-26)] belonging to the other.

The ^13^C NMR spectrum revealed a ^3^*J*_C-F_ triplet splitting (3.2 Hz) for δ_C_ 152.0, which allowed the assignment of C-13. This was supported by the HMBC correlation observed from the difluoromethoxy proton δ_H_ 7.36 to δ_C_ 152.0. Furthermore, HMBC correlations of H-12 and H-14 to δ_C_ 124.8 (C-10) and H-11 and H-15 to δ_C_ 152.0 (C-13) and δ_C_ 146.3 confirmed that this ring system attached to the triazolo ring at position 3.

**Figure 5:**
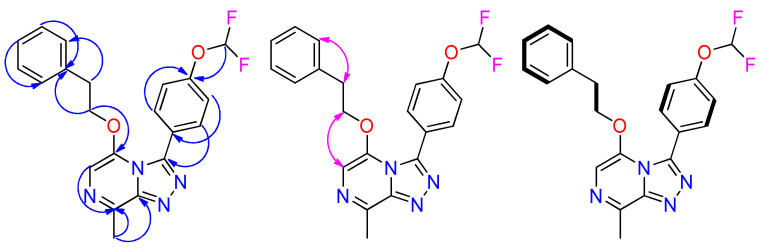
Key HMBC (→), ROESY (↔), and COSY (**—**) correlations for **13**.

The presence of phenyl ether sidechain was supported by the HMBC correlations from the aromatic protons δ_H_ 6.90 to δ_C_ 33.9 (C-22), and δ_H_ 4.42 to δ_C_ 137.4 (C-23) and δ_C_ 142.8 (C-5) of the pyrazine ring. This was further supported by a strong ROE correlation from H-6 (δ_H_ 7.39) to the methylene at H-21 (δ_H_ 4.42). The methyl group protons H-29 showed a two-bond HMBC to the C-8 position and a three-bond HMBC to C-9 (δ_C_ 146.8), confirming its position on the triazolopyrazine core. Key COSY, HMBC, and ROESY correlations for compound **13** are shown in Figure 5.

### 2.3. Late-Stage Functionalization Using Baran Diversinates^™^

The goal was to add a series of fluoroalkyl groups to the 8-position of the triazolopyrazine scaffold **7** using Diversinate^™^ chemistry. To this end, a method was adapted from Kuttruff et al., where a solvent system of 1:1 CH_2_Cl_2_/DMSO was used along with a Diversinate^™^ salt, the oxidant *tert*-butylhydroperoxide (TBHP), and TFA, since protonation of the pyrazine nitrogen adjacent to position 8 was expected to facilitate the radical addition [20].

**Scheme 6:**
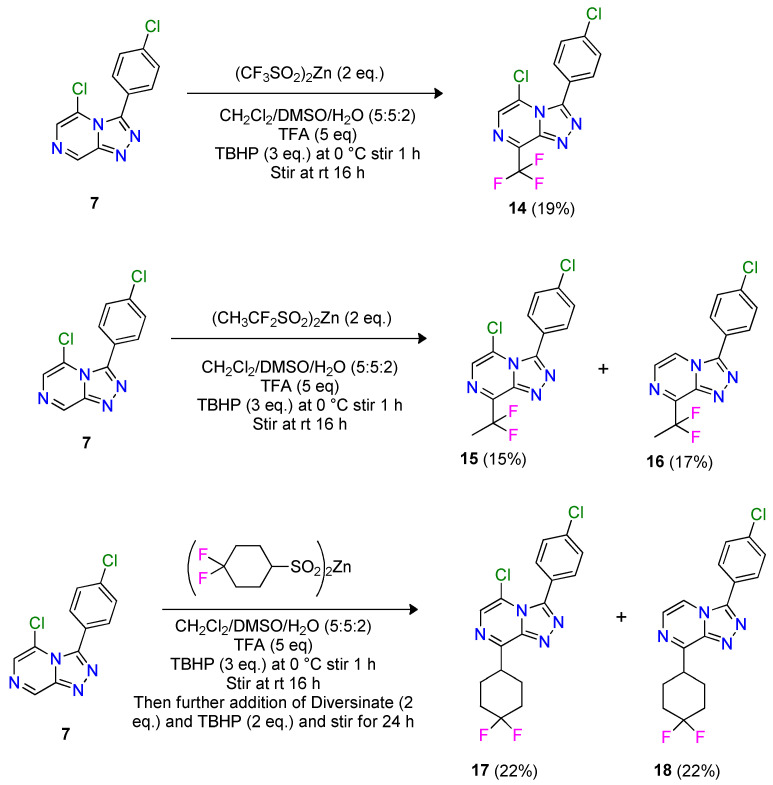
Introduction of fluoroalkyl groups via Diversinate^™^ chemistry.

The Diversinate^™^ reagents investigated were zinc trifluoromethanesulfinate (TFMS) sodium 1,1-difluoroethanesulfinate (DFES), sodium 4,4-difluorocyclohexanesulfinate (DFHS), and sodium 1-(trifluoromethyl)cyclopropanesulfonate (TFCS). The reaction was carried out as follows: a mixture of the scaffold **7**, Diversinate^™^ (2 eq.), and TFA (5 eq.) in DMSO/CH_2_Cl_2_/H_2_O (5:5:2) was stirred for 30 min at room temperature and then cooled to 4 °C. Aqueous TBHP (70%, 3 eq.) was then added over five minutes and stirring continued for 1 h before slowly warming to rt with stirring for a further 16 h. The reaction mixture was then dried under vacuum, and products were isolated and purified using C_18_ HPLC (MeOH/H_2_O/0.1% TFA). This methodology was successful with three of the four Diversinates^™^ (Scheme 6), with only the TFCS reaction failing to produce a product. Two of the products were unexpected. These were the 5-dechlorinated products **16** and **18**.

**Scheme 7:**
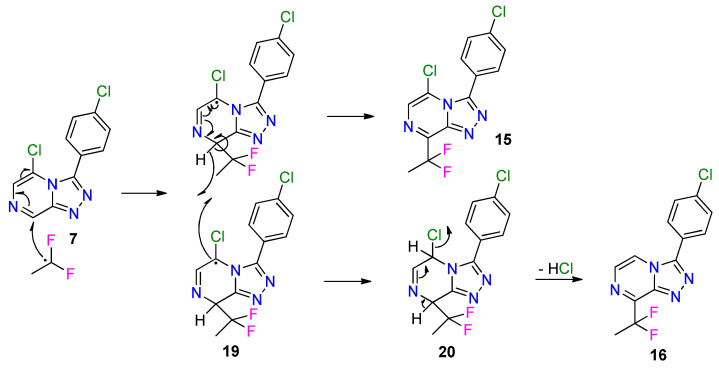
Proposed mechanism for the formation of compound **15** and dechlorinated product **16**.

A mechanism for the formation of these dechlorinated products is suggested in Scheme 7. For example, addition of the 1,1-dichloroethyl radical to the scaffold **7** would generate the radical **19**. As **19** is stabilized by resonance, it could achieve a sufficient concentration to undergo a radical disproportionation reaction whereby the radical center of one molecule of **19** abstracts a hydrogen atom from a second molecule of **19** to generate a molecule of product **15** and the dihydro intermediate **20**. Loss of HCl (water could act as a base here) from **20** generates the 5-dechlorinated product **16**. Compelling evidence for this mechanism is that it predicts equal amounts of **15** and **16** and equal amounts of **17** and **18**, as found. What is not clear is why the formation of **14** was not accompanied by formation of the corresponding 5-dechlorinated product. Possibly the mechanism of formation of **14** did not involve disproportionation, but instead involved hydrogen atom abstraction by the CF_3_ radical (the CF_3_ radical would be expected to be more electrophilic than the other two fluoroalkyl radicals).

**Figure 6:**
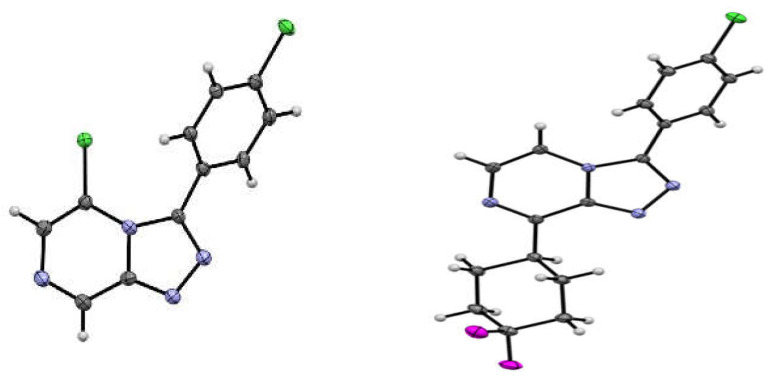
ORTEP drawing of compounds **7** and **18**.

The products **14**–**18** were characterized by 1D/2D NMR and HRMS, and the structure of **18** was confirmed by X-ray crystal structure determination (see Supplementary Materials). Furthermore, during C_18_ HPLC purification of the Diversinate^™^ reactions described above, a small amount of X-ray quality crystalline starting material (**7**) was obtained. The ORTEP of compounds **7** and **18** are shown below in Figure 6.

### 2.4. Bioactivity and Preliminary SAR

Using the software DataWarrior^®^ [21], all compounds were found to have no violations of Lipinski’s “Rule of Five” in silico, suggesting the base requirement for orally active drug-like compounds was met [22]. The semisynthesized/functionalized triazolopyrazine compounds and the scaffold triazolopyrazine precursors, compounds **1**–**18**, were tested for their antimalarial activity against the 3D7 (chloroquine-sensitive strain) and Dd2 (chloroquine, pyrimethamine, and mefloquine resistant strain) *P. falciparum* [23]. Where an accurate IC_50_ value could not be determined due to 100% growth inhibition and a plateau not being reached, but where ≥90% growth inhibition was attained at the top screening concentration, an estimated IC_50_ value was calculated. Cytotoxicity data were also acquired for all scaffolds and synthesized compounds using a human embryonic kidney cell line (HEK293) (Table 3) [24], demonstrating that none of the compounds exhibited cytotoxicity against HEK293 cells when tested at concentrations up to 80 μM.

The scaffold compounds **1**, **7,** and **9**, as well as **11**, the methylated derivative of **1**, exhibited poor activity against 3D7 *P. falciparum,* having the respective IC_50_ values of 16.8, 12.6, 18.9, and 12.5 μM. While **11** retained comparable, if not minimally better, activity than **1**, the other methylated scaffold derivatives (**8** and **10**) experienced a reduction in antimalarial potency.

The ether compound **2**, a known OSM compound, exhibited strong activity against the *Pf* 3D7 parasites with an IC_50_ value of 0.3 μM, which is in agreement with activities reported for other OSM participants [14]. Methylation of **2**, generating compound **13**, resulted in a 37-fold decrease in potency with an IC_50_ value of 11.1 μM. Compound **3** demonstrated a poor IC_50_ value of 16.7 μM and its methylated ether derivative **12** did not display ≥90% inhibition, so a predicted IC_50_ value was not calculated. This data indicates methylation at the C-8 position had a detrimental effect on antimalarial activity.

The Diversinate^™^ derivatives of compound **7** showed mixed results, with compounds **14**, **15,** and **16** showing an increase in potency over the parent scaffold compound **7** (IC_50_ = 12.6 μM)**,** while the difluorocyclohexyl functionalized compounds **17** and **18** showed limited activity. Of the Diversinate^™^ derivatives made during these studies, compound **15** (IC_50_ = 1.7 μM), which exhibited a 7.3-fold improvement in potency compared to the parent scaffold (**7**), identified the difluoroethane moiety as a promising functional group that should be applied to other promising leads within the OSM project. Furthermore, with over 30 Diversinate^™^ reagents commercially available, we believe additional LSF investigations on OSM scaffolds using this chemistry are warranted.

The Dd2 data largely followed the same overall trend as the 3D7 results. However, the Dd2 antimalarial activity was reduced across all tested compounds with the exception of compound **13**, which displayed a slight increase in potency when compared to the 3D7 results. Of note, compound **15** remained the most potent functionalized compound generated during these studies upon comparison of both the Dd2 and 3D7 data, while compounds **5**, **11**, **12, 16,** and **17** displayed large decreases in potency when tested against the drug-resistant strain.

## 3. Materials and Methods

### 3.1. General Experimental Procedures

NMR spectra were recorded on a Bruker Avance III 500 MHz spectrometer (Zürich, Switzerland) equipped with a BBFO Smartprobe at 25 °C. The ^1^H and ^13^C NMR chemical shifts were referenced to the solvent peak for DMSO-*d*_6_ (δ_H_ 2.50 and δ_C_ 39.52). A JASCO V-650 UV/Vis spectrophotometer (Tokyo, Japan) was used for recording UV spectra. LRESIMS data were recorded on a Thermo Fisher Scientific MSQ Plus single quadrupole mass spectrometer (Waltham, MA, USA). HRESIMS data were acquired on a Bruker maXis II ETD ESI-qTOF (Bremen, Germany). An open glass column (25 mm × 80 mm) with sintered glass frit and tap was packed with Merck silica gel (40–63 µm, 143 Å) (Darmstadt, Germany) and used for all silica flash columns. Alltech Davisil C_18_-bonded silica (35–75 µm, 150 Å) (Sydney, NSW, Australia) was used for pre-adsorption of crude reaction products then packed into an Alltech guard cartridge (10 mm × 30 mm) (Sydney, NSW, Australia) in order to facilitate semipreparative HPLC. A Thermo Fisher Scientific Dionex Ultimate 3000 HPLC (Waltham, MA, USA) was used for semipreparative HPLC separations. TLC analysis was carried out using Merck silica gel 60 F_254S_ aluminum plates (Darmstadt, Germany). A Thermo Electron C_18_-bonded silica Betasil column (5 µm, 143 Å, 21.2 mm × 150 mm) (Waltham, MA, USA) was used for semipreparative HPLC separation. All solvents used for chromatography, UV, and MS were Honeywell Burdick & Jackson HPLC grade (Muskegon, MI, USA) or RCI Labscan HPLC grade (Bangkok, Thailand). H_2_O was Sartorius arium proVF (Göttingen, Germany) filtered. Synthetic reagents were purchased from Sigma-Aldrich (St. Louis, MO, USA) or AK Scientific Inc. (Union City, CA, USA) and used without further purification. A Kessil PR-160L-456 nm blue light lamp was used as a light source for the photoredox reactions. Kinesis clear glass vials and screw caps (1.5 mL) (Brisbane, QLD, Australia) and an Industrial Equipment & Control Pty Ltd. stirrer hotplate were used for all photoredox and Diversinate^™^ reactions.

### 3.2. General Procedure for Coupling of Primary Alcohol with Chloro-Heterocycle Scaffold

The chloro-heterocycle scaffold (1.70 mmol) and primary alcohol (1 eq., 1.70 mmol) were dissolved in anhydrous toluene (10 mL), along with KOH (312 mg, 5.58 mmol, 3.3 eq.) and 18-crown-6 (36 mg, 0.14 mmol, 0.08 eq.). The reaction mixtures were subsequently stirred at 40 °C for 1 to 3 h with reactions monitored by TLC. Upon reaction completion, the mixture was diluted with H_2_O (60 mL) and then extracted with EtOAc (3 × 20 mL). The organic extracts were combined, dried (Na_2_SO_4_), filtered, and concentrated under reduced pressure to give a crude product that was preadsorbed to silica (~1 g), then purified by silica flash column chromatography using a 10% stepwise gradient from 100% *n*-hexane to 100% EtOAc (100 mL elutions), followed by final flushes with 10% MeOH/90% CH_2_Cl_2_ (100 mL) and 20% MeOH/80% CH_2_Cl_2_ (100 mL). Fractions containing UV-active material, as deemed by TLC, were analyzed by ^1^H NMR spectroscopy and LCMS, in order to identify products of interest; only fractions containing the desired product in high purity (>95%) were combined.

#### 3.2.1. Compound **2**

*3-[4-(Difluoromethoxy)phenyl]-5-(2-phenylethoxy)-[1,2,4]triazolo[4,3-α]pyrazine* (**2**). White amorphous solid (125.4 mg, 33%); UV (MeOH) λ_max_ (log ε) 241 (4.64), 286 (4.11), 315 (4.51) nm; ^1^H NMR (500 MHz, DMSO-*d*_6_) δ_H_ 2.90 (2H, t, *J* = 6.5 Hz, H-22), 4.50 (2H, t, *J* = 6.5 Hz, H-21), 6.91 (2H, m, H-24, H-28), 7.16 (1H, m, H-26), 7.18 (2H, m, H-25, H-27), 7.30 (2H, m, H-12, H-14), 7.37 (1H, t, ^2^*J*_HF_ = 73.7 Hz, H-17), 7.60 (1H, s, H-6), 7.77 (2H, m, H-11, H-15), 9.04 (1H, s, H-8); ^13^C NMR (125 MHz, DMSO-*d*_6_) δ_C_ 33.8 (C-22), 71.2 (C-21), 108.8 (C-6), 116.2 (t, ^1^*J*_CF_ = 258.8 Hz, C-17), 117.6 (C-12, C-14), 124.7 (C-10), 126.3 (C-26), 128.3 (C-24, C-28), 128.6 (C-25, C-27), 132.6 (C-11, C-15), 135.0 (C-8), 137.3 (C-23), 145.5 (C-3), 143.8 (C-5), 147.4 (C-9), 152.0 (t, ^3^*J*_CF_ = 3.2 Hz, C-13); (+)-LRESIMS *m/z* (rel. int.) 383 (100) [M + H]^+^, 765 (30) [2M + H]^+^; (+)-HRESIMS *m/z* 383.1315 [M + H]^+^ (calcd for C_20_H_17_F_2_N_4_O_2_, 383.1314), 405.1133 [M + Na]^+^ (calcd for C_20_H_16_F_2_N_4_NaO_2_, 405.1134).

#### 3.2.2. Compound **3**

*3-[(4-Difluoromethoxy)phenyl]-5-(2,2,2,-trifluroethoxy)-[1,2,4]triazolo[4,3-α]pyrazine* (**3**). White amorphous solid (105.3 mg, 17%); mp 169–170 °C; UV (MeOH) λ_max_ (log ε) 239 (4.95), 282 (4.54), 311 (4.48) nm; ^1^H NMR (500 MHz, DMSO-*d*_6_) δ_H_ 5.05 (2H, q, ^2^*J*_HF_ = 8.5 Hz, H-21), 7.29 (2H, m, H-12, H-14), 7.35 (1H, t, ^2^*J*_HF_ = 73.8 Hz, H-17), 7.69 (1H, s, H-6), 7.79 (2H, m, H-11, H-15), 9.15 (1H, s, H-8); ^13^C NMR (125 MHz, DMSO-*d*_6_) δ_C_ 66.1 (q, ^2^*J*_CF_ = 36.2 Hz, C-21), 109.7 (C-6), 116.1 (t, ^1^*J*_CF_ = 258.8 Hz, C-17), 117.7 (C-12, C-14), 122.6 (q, ^1^*J*_CF_ = 277.2 Hz, C-22), 124.4 (C-10), 132.4 (C-11, C-15), 136.8 (C-8), 142.6 (C-5), 145.7 (C-3), 147.4 (C-9), 152.0 (t, ^3^*J*_CF_ = 3.3 Hz, C-13); (+)-LRESIMS *m/z* (rel. int.) 361 (100) [M + H]^+^; (+)-HRESIMS *m/z* 383.0536 [M + H]^+^ (calcd for C_14_H_9_F_5_N_4_NaO_2_, 383.0538).

#### 3.2.3. Compound **4**

*3-[4-(Difluoromethoxy)phenyl]-[1,2,4]triazolo[4,3-α]pyrazin-8-ol* (**4**). Beige amorphous solid (28.7 mg, 6%); UV (MeOH) λ_max_ (log ε) 247 (4.83), 293 (4.26) nm; ^1^H NMR (500 MHz, DMSO-*d*_6_) δ_H_ 6.92 (1H, d, *J* = 5.8 Hz, H-6), 7.40 (1H, t, ^2^*J*_HF_ = 73.6 Hz, H-17), 7.42 (2H, m, H-12, H-14), 7.43 (1H, d, *J* = 5.8 Hz, H-5), 7.91 (2H, m, H-11, H-15), 11.48 (1H, br s, H-20); ^13^C NMR (125 MHz, DMSO-*d*_6_) δ_C_ 103.9 (C-5), 116.1 (t, ^1^*J*_CF_ = 258.7 Hz, C-17), 118.7 (C-6), 119.2 (C-12, C-14), 122.6 (C-10), 130.4 (C-11, C-15), 145.3 (C-9), 152.5 (t, ^3^*J*_CF_ = 3.3 Hz, C-13), 153.0 (C-8); (+)-LRESIMS *m/z* (rel. int.) 279 (100) [M + H]^+^; (+)-HRESIMS *m/z* 279.0688 [M + H]^+^ (calcd for C_12_H_9_F_2_N_4_O_2_, 279.0688), 301.0507 [M + Na]^+^ (calcd for C_12_H_8_F_2_N_4_NaO_2_, 301.0508).

#### 3.2.4. Compound **5**

*3-[4-(Difluoromethoxy)phenyl]-5-[2-trimethylsilyl)ethoxy]-[1,2,4]triazolo[4,3-α]pyrazine* (**5**). Brown crystals (153.2 mg, 24%); mp 135 °C; UV (MeOH) λ_max_ (log ε) 243 (4.48), 317 (3.96) nm; ^1^H NMR (500 MHz, DMSO-*d*_6_) δ_H_ 0.07 (9H, s, H-24, H-25, H-26), 0.91 (2H, m, H-22), 4.33 (2H, m, H-21), 7.31 (2H, m, H-12, H-14), 7.36 (1H, t, ^2^*J*_HF_ = 73.6 Hz, H-17), 7.59 (1H, s, H-6), 7.81 (2H, m, H-11, H-15), 9.01 (1H, s, H-8); ^13^C NMR (125 MHz, DMSO-*d*_6_) δ_C_ -1.7 (C-24, C-25, C-26), 16.5 (C-22), 69.4 (C-21), 109.1 (C-6), 116.1 (t, ^1^*J*_CF_ = 258.3 Hz, C-17), 117.6 (C-12, C-14), 124.8 (C-10), 132.8 (C-11, C-15), 134.7 (C-8), 143.9 (C-5), 147.5 (C-9), 145.6 (C-3), 151.9 (t, ^3^*J*_CF_ = 3.3 Hz, C-13); (+)-LRESIMS *m/z* (rel. int.) 379 (26) [M + H]^+^, 675 (60) [2M + H]^+^; (+)-HRESIMS *m/z* 379.1396 [M + H]^+^ (calcd for C_17_H_21_F_2_N_4_O_2_Si, 379.1396), 401.1216 [M + Na]^+^ (calcd for C_17_H_20_F_2_N_4_NaO_2_Si, 401.1216).

#### 3.2.5. Compound **6**

*3-[4-(Difluoromethoxy)phenyl]-8-[2-(trimethylsilyl)ethoxy)-[1,2,4]triazolo[4,3-α]pyrazine* (**6**). White amorphous solid (153.0 mg, 24%); UV (MeOH) λ_max_ (log ε) 243 (4.73), 276 (4.43) nm; ^1^H NMR (500 MHz, DMSO-*d*_6_) δ_H_ 0.11 (9H, s, H-24, H-25, H-26), 1.24 (2H, m, H-22), 4.65 (2H, m, H-21), 7.41 (1H, t, ^2^*J*_HF_ = 73.7 Hz, H-17), 7.43 (2H, m, H-12, H-14), 7.47 (1H, d, *J* = 4.9 Hz, H-6), 7.98 (2H, m, H-11, H-15), 8.17 (1H, d, *J* = 4.9 Hz, H-5); ^13^C NMR (125 MHz, DMSO-*d*_6_) δ_C_ -1.3 (C-24, C-25, C-26), 16.8 (C-22), 65.2 (C-21), 111.9 (C-5), 116.1 (t, ^1^*J*_CF_ = 258.4 Hz, C-17), 119.2 (C-12, C-14), 122.7 (C-10), 130.2 (C-11, C-15), 139.9 (C-9), 152.4 (t, ^3^*J*_CF_ = 3.3 Hz, C-13), 153.3 (C-8); (+)-LRESIMS *m/z* (rel. int.) 379 (100) [M + H]^+^; (+)-HRESIMS *m/z* 379.1397 [M + H]^+^ (calcd for C_17_H_21_F_2_N_4_O_2_Si, 379.1396), 401.1216 [M + Na]^+^ (calcd for C_17_H_20_F_2_N_4_NaO_2_Si, 401.1216).

### 3.3. General Procedure for Photoredox Catalysis Methylation of Heterocyclic Scaffolds

The photocatalyst ([Ir(dF-CF_3_-ppy)_2_(dtbpy)]PF_6_) (2.3 mg, 0.002 mmol, 0.02 eq.), heterocyclic scaffold (0.1 mmol) and solvent (1 mL) (1:1 ACN:TFA), was added to a clear, glass vial and the solution was degassed using nitrogen for 3 min. *Tert*-butyl peracetate (96 µL, 50% solution in mineral spirits, 0.3 mmol, 3.0 eq.) was added and the reaction vial was positioned on top of a light intensity map that was attached to the top of the stirrer hotplate. A calibrated blue light lamp (Kessil PR-160L-456nm) was then aimed at the reaction vial in relation to the intensity map (15 W/cm^2^ to 300 W/cm^2^). Compounds **8** and **10** were generated using a blue light intensity of 45 W/cm^2^, while compounds **11**, **12,** and **13** were produced using 20 W/cm^2^. All reactions were stirred and irradiated for 16 h at room temperature. Upon the completion of the reaction, the mixture was preadsorbed to C_18_-bonded silica (~1 g) and then loaded into a guard cartridge that was subsequently attached to a semipreparative C_18_-bonded silica HPLC column. Isocratic conditions of 10% MeOH/90% H_2_O (0.1% TFA) were held for the first 10 min, followed by a linear gradient to 100% MeOH (0.1% TFA) over 40 min, then isocratic conditions of 100% MeOH (0.1% TFA) for an additional 10 min, all at a flow rate of 9 mL/min. Sixty fractions (60 × 1 min) were collected from the start of the HPLC run. Fractions containing UV-active material from each separate HPLC run were analyzed by ^1^H NMR spectroscopy and LCMS, and high purity (>95%) fractions were combined to give total yield.

#### 3.3.1. Compound **8**

*5-Chloro-3-(4-chlorophenyl)-8-methyl-[1,2,4]triazolo[4,3-α]pyrazine* (**8**). Pale pink amorphous solid (3.9 mg, 14%); UV (MeOH) λ_max_ (log ε) 224 (4.44), 241 (4.42), 308 (3.83) nm; ^1^H NMR (500 MHz, DMSO-*d*_6_) δ_H_ 2.86 (3H, s, H-18), 7.63 (2H, m, H-12, H-14), 7.74 (2H, m, H-11, H-15) 7.92 (1H, s, H-6); ^13^C NMR (125 MHz, DMSO-*d*_6_) δ_C_ 20.2 (C-18), 119.8 (C-5), 126.3 (C-10), 127.9 (C-12, C-14), 128.5 (C-6), 133.1 (C-11, C-15), 135.4 (C-13), 146.7 (C-9), 147.2 (C-3), 151.5 (C-8); (+)-LRESIMS *m/z* (rel. int.) 279 (100) [M + H]^+^; (+)-HRESIMS *m/z* 279.0197 [M + H]^+^ (calcd for C_12_H_9_Cl_2_N_4_, 279.0199), 301.0015 [M + Na]^+^ (calcd for C_12_H_8_Cl_2_N_4_Na, 301.0018).

#### 3.3.2. Compound **10**

*4-(5-Chloro-8-methyl-1,2,4-triazolo[4,3-α]pyrazin-3-yl)benzonitrile* (**10**). Pale red amorphous solid (4.6 mg, 17%); UV (MeOH) λ_max_ (log ε) 225 (4.32), 235 (4.29), 251 (4.19), 282 (4.02) nm; ^1^H NMR (500 MHz, DMSO-*d*_6_) δ_H_ 2.87 (3H, s, H-19), 7.93 (2H, m, H-11, H-15), 7.96 (1H, s, H-6), 8.04 (2H, m, H-12, H-14); ^13^C NMR (125 MHz, DMSO-*d*_6_) δ_C_ 20.2 (C-19), 113.0 (C-13), 118.4 (C-16), 119.8 (C-5), 128.7 (C-6), 146.76^#^ (C-3), 146.83^#^ (C-9), 151.5 (C-8), 132.0 (C-10), 132.2 (C-11, C-15), 131.6 (C-12, C-14); (+)-LRESIMS *m/z* (rel. int.) 270 (100) [M + H]^+^; (+)-HRESIMS *m/z* 270.0540 [M + H]^+^ (calcd for C_13_H_9_ClN_5_, 270.0541), 292.0358 [M + Na]^+^ (calcd for C_13_H_8_ClN_5_Na, 292.0360). ^#^ Interchangeable signals

#### 3.3.3. Compound **11**

*5-Chloro-3-[(4-difluoromethoxy)phenyl]-8-methyl-[1,2,4]triazolo[4,3-α]pyrazine* (**11**). Brown amorphous solid (12.3 mg, 40%); UV (MeOH) λ_max_ (log ε) 239 (3.90), 309 (3.29) nm; ^1^H NMR (500 MHz, DMSO-*d*_6_) δ_H_ 2.86 (3H, s, H-21), 7.34 (2H, m, H-12, H-14), 7.40 (1H, t, ^2^*J*_HF_ = 73.7 Hz, H-17), 7.77 (2H, m, H-11, H-15), 7.91 (1H, s, H-6); ^13^C NMR (125 MHz, DMSO-*d*_6_) δ_C_ 20.2 (C-21), 116.2 (t, ^1^*J*_CF_ = 258.5 Hz, C-17), 117.5 (2C, C-12, C-14), 119.8 (C-5), 124.0 (C-10), 128.5 (C-6), 133.3 (C-11, C-15), 146.7 (C-9), 147.4 (C-3), 151.5 (C-8), 152.5 (t, ^3^*J*_CF_ = 3.3 Hz, C-13); (+)-LRESIMS *m/z* (rel. int.) 311 (100) [M + H]^+^; (+)-HRESIMS *m/z* 311.0508 [M + H]^+^ (calcd for C_13_H_10_ClF_2_N_4_O, 311.0506), 333.0327 [M + Na]^+^ (calcd for C_13_H_9_ClF_2_N_4_NaO, 333.0325).

#### 3.3.4. Compound **12**

*3-[4-(Difluoromethoxy)phenyl]-8-methyl-5-(2,2,2-trifluoroethoxy)-[1,2,4]triazolo[4,3-a]pyrazine* (**12**). White amorphous solid (8.5 mg, 22%); UV (MeOH) λ_max_ (log ε) 240 (5.00), 309 (4.45) nm; ^1^H NMR (500 MHz, DMSO-*d*_6_) δ_H_ 2.78 (3H, s, H-26), 4.96 (2H, q, ^2^*J*_HF_ = 8.5 Hz, H-21), 7.28 (2H, m, H-12, H-14), 7.35 (1H, t, ^2^*J*_HF_ = 73.7 Hz, H-17), 7.52 (1H, s, H-6), 7.76 (2H, m, H-11, H-15); ^13^C NMR (125 MHz, DMSO-*d*_6_) δ_C_ 19.8 (C-26), 66.0 (q, ^2^*J*_CF_ = 35.8 Hz, C-21), 108.9 (C-6), 116.1 (t, ^1^*J*_CF_ = 258.5 Hz, C-17), 117.7 (C-12, C-14), 122.5 (q, ^1^*J*_CF_ = 277.3 Hz, C-22), 124.4 (C-10), 132.4 (C-11, C-15), 145.1 (C-8), 141.6 (C-5), 146.8 (C-3), 146.4 (C-9), 152.0 (t, ^3^*J*_CF_ = 3.2 Hz, C-13); (+)-LRESIMS *m/z* (rel. int.) 375 (100) [M + H]^+^; (+)-HRESIMS *m/z* 375.0874 [M + H]^+^ (calcd for C_15_H_12_F_5_N_4_O_2_, 375.0875), 397.0693 [M + Na]^+^ (calcd for C_15_H_11_F_5_N_4_NaO_2_, 397.0694).

#### 3.3.5. Compound **13**

*3-[4-(Difluoromethoxy)phenyl]-8-methyl-5-(2-phenylethoxy)-[1,2,4]triazolo[4,3-a]pyrazine* (**13**). White amorphous solid (17.1 mg, 43%); UV (MeOH) λ_max_ (log ε) 244 (4.34), 312 (3.81) nm; ^1^H NMR (500 MHz, DMSO-*d*_6_) δ_H_ 2.72 (3H, s, H-29), 2.86 (2H, t, *J* = 6.5 Hz, H-22), 4.42 (2H, t, *J* = 6.5 Hz, H-21), 6.91 (2H, m, H-24, H-28), 7.16 (1H, m, H-26), 7.17 (2H, m, H-25, H-27), 7.29 (2H, m, H-12, H-14), 7.36 (1H, t, ^2^*J*_HF_ = 73.7 Hz, H-17), 7.39 (1H, s, H-6), 7.74 (2H, m, H-11, H-15); ^13^C NMR (125 MHz, DMSO-*d*_6_) δ_C_ 19.7 (C-29), 33.9 (C-22), 71.0 (C-21), 107.9 (C-6), 116.2 (t, ^1^*J*_CF_ = 258.2 Hz, C-17), 117.6 (2C, C-12, C-14), 124.8 (C-10), 126.4 (C-26), 128.2 (C-25, C-27), 128.7 (C-24, C-28), 132.6 (C-11, C-15), 137.4 (C-23), 142.8 (C-5), 143.2 (C-8), 146.3 (C-3), 146.8 (C-9), 152.0 (t, ^3^*J*_CF_ = 3.2 Hz, C-13); (+)-LRESIMS *m/z* (rel. int.) 397 (100) [M + H]^+^; (+)-HRESIMS *m/z* 397.1470 [M + H]^+^ (calcd for C_21_H_19_F_2_N_4_O_2_, 397.1471), 419.1290 [M + Na]^+^ (calcd for C_21_H_18_F_2_N_4_NaO_2_, 419.1290).

### 3.4. General Procedure for the Late-Stage Functionalization Using Baran Diversinates^™^ on Heterocyclic Scaffolds

The scaffold (0.1 mmol) was dissolved in DMSO/CH_2_Cl_2_ (1:1, 250 µL: 250 µL) before the addition of Diversinate^™^ (0.2 mmol, 2 eq.), TFA (40 μL, 0.5 mmol, 5 eq.), and filtered H_2_O (100 μL). Reaction mixtures were stirred for 30 min at room temperature, cooled to 4 °C. Then, 70% TBHP (41 μL, 0.3 mmol, 3 eq.) was slowly added over 5 min and left to stir for 16 h. All reactions were monitored by LCMS at 16 h. If a minimal product had formed at 16 h, an additional 0.2 mmol of both Diversinate^™^ and 0.3 mmol of TBHP was added and the reaction stirred for 24 h at room temperature prior to purification. Each crude reaction product was preadsorbed to C_18_-bonded silica (~1 g) and then packed into a guard cartridge that was subsequently attached to a semipreparative C_18_-bonded silica HPLC column. Isocratic conditions of 10% MeOH/90% H_2_O (0.1% TFA) were held for the first 10 min, followed by a linear gradient to 100% MeOH (0.1% TFA) over 40 min, then isocratic conditions of 100% MeOH (0.1% TFA) for an additional 10 min, all at a flow rate of 9 mL/min. Sixty fractions (60 × 1 min) were collected from the start of the HPLC run. Fractions containing UV-active material were analyzed by ^1^H NMR spectroscopy and LCMS, in order to identify products of interest.

#### 3.4.1. Compound **14**

*5-Chloro-3-(4-chlorophenyl)-8-(trifluoromethyl)-[1,2,4]triazolo[4,3-α]pyrazine* (**14**). Yellow amorphous solid (6.4 mg, 19%); UV (MeOH) λ_max_ (log ε) 229 (4.18), 245 (4.14), 330 (3.30) nm; ^1^H NMR (500 MHz, DMSO-*d*_6_) δ_H_ 7.67 (2H, m, H-12, H-14), 7.75 (2H, m, Hz, H-11, H-15), 8.03 (1H, s, H-6); ^13^C NMR (125 MHz, DMSO-*d_6_*) δ_C_ 119.9 (q, ^1^*J*_C-F_ = 275.1 Hz, C-18), 125.6 (C-10), 143.4 (C-9), 127.8 (C-6), 128.0 (C-12, C-14), 133.2 (C-11, C-15), 135.8 (C-13), 137.4 (q, ^2^*J*_CF_ = 37.9 Hz, C-8), 126.3 (C-5), 147.5 (C-3); (+)-LRESIMS *m/z* (rel. int.) 333 (100) [M + H]^+^; (+)-HRESIMS *m/z* 332.9915 [M + H]^+^ (calcd for C_12_H_6_Cl_2_F_3_N_4_, 332.9916), 354.9734 [M + Na]^+^ (calcd for C_12_H_5_Cl_2_F_3_N_4_Na, 354.9736).

#### 3.4.2. Compound **15**

*5-Chloro-3-(4-chlorophenyl)-8-(1,1-difluoroethyl)-[1,2,4]triazolo[4,3-a]pyrazine* (**15**). Yellow amorphous solid (*t*_R:_ 37 min, 4.8 mg, 15%); UV (MeOH) λ_max_ (log ε) 227 (5.00), 240 (4.98), 311 (4.24) nm; ^1^H NMR (500 MHz, DMSO-*d*_6_) δ_H_ 2.25 (3H, t, ^3^*J*_HF_ = 19.3 Hz, H-20), 7.65 (2H, m, H-12, H-14), 7.75 (2H, m, H-11, H-15), 8.17 (1H, s, H-6); ^13^C NMR (125 MHz, DMSO-*d_6_*) δ_C_ 22.8 (t, ^2^*J*_CF_ = 25.5 Hz, C-20), 119.1 (t, ^1^*J*_CF_ = 240.2 Hz, C-19), 124.2 (C-5), 126.0 (C-10), 127.6 (C-6), 127.9 (C-12, C-14), 133.0 (C-11, C-15), 135.7 (C-13), 143.9 (C-9), 144.9 (t, ^2^*J*_C-F_ = 31.3 Hz, C-8), 147.1 (C-3); (+)-LRESIMS *m/z* (rel. int.) 329 (100) [M + H]^+^; (+)-HRESIMS *m/z* 329.0166 [M + H]^+^ (calcd for C_13_H_9_Cl_2_F_2_N_4_, 329.0167), 350.9985 [M + Na]^+^ (calcd for C_13_H_8_Cl_2_F_2_N_4_Na, 350.9986).

#### 3.4.3. Compound **16**

*3-(4-Chlorophenyl)-8-(1,1-difluoroethyl)-[1,2,4]triazolo[4,3-a]pyrazine* (**16**). Yellow amorphous solid (*t*_R:_ 35 min, 5.0 mg, 17%); UV (MeOH) λ_max_ (log ε) 248 (4.89), 279 (4.45) nm; ^1^H NMR (500 MHz, DMSO-*d*_6_) δ_H_ 2.25 (3H, t, ^3^*J*_HF_ = 19.3 Hz, H-18), 7.73 (2H, m, H-12, H-14), 7.98 (2H, m, H-11, H-15), 8.04 (1H, d, *J* = 4.8 Hz, H-6), 8.74 (1H, d, *J* = 4.8 Hz, H-5); ^13^C NMR (125 MHz, DMSO-*d_6_*) δ_C_ 22.7 (t, ^2^*J*_C-F_ = 25.6 Hz, C-18), 119.5 (C-5), 120.0 (t, ^1^*J*_C-F_ = 239.7 Hz, C-17), 124.4 (C-10), 128.5 (C-6), 129.5 (C-12, C-14), 130.3 (C-11, C-15), 135.6 (C-13), 142.6 (C-9), 146.4 (t, ^2^*J*_C-F_ = 30.9 Hz, C-8), 146.6 (C-3); (+)-LRESIMS *m/z* (rel. int.) 295 (100) [M + H]^+^; (+)-HRESIMS *m/z* 295.0557 [M + H]^+^ (calcd for C_13_H_10_ClF_2_N_4_, 295.0557), 317.0378 [M + Na]^+^ (calcd for C_13_H_9_ClF_2_N_4_Na, 317.0376).

#### 3.4.4. Compound **17**

*5-Chloro-3-(4-chlorophenyl)-8-(4,4-difluorocyclohexyl)-[1,2,4]triazolo[4,3-a]pyrazine* (**17**). Yellow amorphous solid (*t*_R:_ 35 min, 8.5 mg, 22%); UV (MeOH) λ_max_ (log ε) 227 (4.78), 244 (4.79), 309 (4.12) nm; ^1^H NMR (500 MHz, DMSO-*d*_6_) δ_H_ 2.00 and 2.14 (4H, m, H-20, H-24), 2.08 and 2.17 (4H, m, H-21, H-23), 3.75 (1H, m, H-19), 7.62 (2H, m, H-12, H-14), 7.74 (2H, m, H-11, H-15), 7.99 (1H, s, H-6); ^13^C NMR (125 MHz, DMSO-*d_6_*) δ_C_ 26.7 (d, ^3^*J*_CF_ = 9.6 Hz, C-20, C-24), 32.6 (2C, dd, ^2^*J*_CF_ = 23.8, 23.8 Hz C-21, C-23), 38.5 (C-19), 120.1 (C-5), 123.9 (dd, ^1^*J*_CF_ = 239.3, 241.6 Hz, C-22), 126.3 (C-10), 127.8 (C-12, C-14), 128.5 (C-6), 133.2 (C-11, C-15), 135.4 (C-13), 145.7 (C-9), 147.1 (C-3), 156.3 (C-8); (+)-LRESIMS *m/z* (rel. int.) 383 (100) [M + H]^+^; (+)-HRESIMS *m/z* 383.0636 [M + H]^+^ (calcd for C_17_H_15_Cl_2_F_2_N_4_, 383.0636), 405.0456 [M + Na]^+^ (calcd for C_17_H_14_Cl_2_F_2_N_4_Na, 405.0456).

#### 3.4.5. Compound **18**

*3-(4-Chlorophenyl)-8-(4,4-difluorocyclohexyl)-[1,2,4]triazolo[4,3-a]pyrazine* (**18**). Yellow crystals (*t*_R:_ 35 min, 7.7 mg, 22%); mp 203–205 °C UV (MeOH) λ_max_ (log ε) 247 (4.91), 279 (4.57) nm; ^1^H NMR (500 MHz, DMSO-*d*_6_) δ_H_ 2.03 and 2.15 (4H, m, H-19, H-23), 2.06 and 2.17 (4H, m, H-20, H-22), 3.75 (1H, m, H-18), 7.71 (2H, m, H-12, H-14), 7.97 (2H, m, H-11, H-15), 7.90 (1H, d, *J* = 4.9 Hz, H-5), 8.49 (1H, d, *J* = 4.9 Hz, H-6); ^13^C NMR (125 MHz, DMSO-*d_6_*) δ_C_ 26.7 (d, ^3^*J*_CF_ = 9.6 Hz, C-18, C-22), 32.6 (dd, ^2^*J*_C-F_ = 23.8, 23.8 Hz, C-19, C-21), 38.9 (C-17), 115.6 (C-5), 123.9 (dd, ^1^*J*_CF_ = 239.5, 241.6 Hz, C-20), 124.7 (C-10), 129.4 (C-12, C-14), 129.6 (C-6), 130.0 (C-11, C-15), 135.3 (C-13), 144.7 (C-9), 146.4 (C-3), 157.6 (C-8); (+)-LRESIMS *m/z* (rel. int.) 349 (100) [M + H]^+^; (+)-HRESIMS *m/z* 349.1027 [M + H]^+^ (calcd for C_17_H_16_ClF_2_N_4_, 349.1026), 371.0846 [M + Na]^+^ (calcd for C_17_H_15_ClF_2_N_4_Na, 371.0846).

### 3.5. X-ray Crystallography Studies on Compounds **3**, **5**, **7,** and **18**

Intensity data for compounds were collected with an Oxford Diffraction Synergy diffractometer using either Cu-K*α* radiation or Mo-K*α*. The temperature during data collection was maintained at 100.0(1) K using an Oxford Cryosystems cooling device. The structure of each compound was solved by direct methods and difference Fourier Synthesis [25]. Hydrogen atoms bound to the carbon atom were placed at their idealized positions and included in subsequent refinement cycles. The hydrogen atoms attached to heteroatoms were located from different Fourier maps and refined freely with isotropic displacement parameters. Thermal ellipsoid plots were generated using the program Mercury [26] integrated within the WINGX suite of programs [27]. Crystallographic data for compounds **3**, **5**, **7,** and **18** were deposited with the Cambridge Crystallographic Data Centre and assigned CCDC deposit codes 2068891–2068894, respectively.

#### 3.5.1. Crystal Data for Compound **3**

C_14_H_9_N_4_O_2_F_5_, *M* = 360.25, mp 169–170 °C, *T* = 100.0(10) K, λ = 1.54184 Å, Orthorhombic, space group *P bca a =* 7.2017(1), *b =* 14.1975(1), *c =* 27.9870(3) Å*, V =* 2861.56(5) Å^3^, *Z* = 8, *D_c_* = 1.672 Mg M^−3^ μ (Cu-K*α*) = 1.413 mm^−1^, *F(000)* = 1456, crystal size 0.298 × 0.222 × 0.084 mm^3^ θ_max_ = 77.25°, 35469 reflections measured, 3013 independent reflections (R_int_ = 0.0446); the final R = 0.0360 [I > 2σ (I), 2809 data] and *w*R(F^2^) = 0.0949 (all data); GOOF = 1.045.

#### 3.5.2. Crystal Data for Compound **5**

C_17_H_20_F_2_N_4_O_2_Si, *M* = 378.46, mp 135 °C, *T* = 100.0(10) K, λ = 0.71073 Å, Triclinic, space group *P-1 a =* 7.6807(2), *b =* 15.5577(4), *c =* 15.8460(5) Å*,* α = 76.888(2)° β = 77.685(2)° γ = 89.799(2)° *V =* 1799.70(9) Å^3^, *Z* = 4, Z’=2, *D_c_* = 1.397 Mg M^-3^ μ (Mo-K*α*) = 0.170 mm^−1^, *F(000)* = 792, crystal size 0.568 × 0.305 × 0.230 mm^3^ θ_max_ = 37.28°, 30807 reflections measured, 13451 independent reflections; (R_int_ = 0.0548) the final R = 0.0685 [I > σ2(I), 9692 data] and *w*R(F^2^) = 0.2131 (all data); GOOF = 1.049.

#### 3.5.3. Crystal Data for Compound **7**

C_11_H_6_N_4_Cl_2_, *M* = 265.10, mp 175–176 °C, *T* = 100.0(10) K, λ = 1.54184 Å, Orthorhombic, space group *P 2_1_2_1_2_1_ a =* 3.8654(1), *b =* 6.6836(2), *c =* 41.0638(11) Å*, V =* 1060.87(5) Å^3^, *Z* = 4, *D_c_* = 1.660 Mg M^-3^ μ (Cu-K*α*) = 5.341 mm^−1^, *F(000)* = 1456, crystal size 0.258 × 0.066 × 0.024 mm^3^ θ_max_ = 77.14°, 3732 reflections measured, 1975 independent reflections (R_int_ = 0.0464); the final R = 0.0488 [I > 2σ (I), 1835 data] and *w*R(F^2^) = 0.1236 (all data); GOOF = 1.015.

#### 3.5.4. Crystal Data for Compound **18**

C_17_H_15_F_2_N_4_Cl, *M* = 348.78, mp 203–205 °C, *T* = 100.0(10) K, λ = 0.71073 Å, Triclinic, space group *P-1 a =* 8.9632(2), *b =* 11.2309(4), *c =* 16.0212(6) Å*,* α= 103.560(3)° β = 94.906(2)° γ = 97.188(3)° *V =* 1544.42(9) Å^3^, *Z* = 4, Z’ = 2, *D_c_* = 1.500 Mg M^−3^ μ (Mo-K*α*) = 0.276 mm^−1^, *F(000)* = 720, crystal size 0.570 × 0.297 × 0.031 mm^3^ θ_max_ = 37.19°, 33358 reflections measured, 14415 independent reflections (R_int_ = 0.0453); the final R = 0.0473 [I > 2σ (I), 10306 data] and *w*R(F^2^) = 0.1293 (all data); GOOF = 1.073.

### 3.6. In Vitro Anti-Plasmodial Image-Based Asexual Assay

*Plsamodium falciparum* 3D7 and Dd2 parasites were cultured in RPMI1640 (Life Technologies, Camarillo, CA, USA) supplemented with 2.5 mg/mL Albumax II, 5% AB human serum, 25 mM HEPES, and 0.37mM hypoxanthine. Ring stage parasites were treated with compounds following two rounds of sorbitol synchronization, as previously described [23]. Artesunate and pyrimethamine were incorporated as controls. Following incubation of assay plates for 72 h at 37 °C, and 5% CO_2_ and 5% O_2_, parasites were stained with 2-(4-amidinophenyl)-1H-indole-6-carboxamidine (DAPI) and imaged using an Opera QEHS confocal imaging system (PerkinElmer, Waltham, MA, USA). Images were analyzed, as previously described, using Acapella spot detection software (PerkinElmer, Waltham, MA, USA) [23].

### 3.7. In Vitro Cytotoxicity Assay

Human embryonic kidney cells (HEK293) were maintained in DMEM (Life Technologies, Camarillo, CA, USA) containing 10% FBS (Hyclone™ ThermoFisher, Melbourne, Australia). Cytotoxicity testing was undertaken, as previously described [24]. In brief, 5 μL of test compound was added/well of black/clear tissue culture-treated, 384-well plates containing 3000 adherent HEK 293 cells/well in 45 μL and incubated 72 h at 37 °C in 5% CO_2_. After incubation, the supernatant was removed and replaced with 40 μL of 10% Alamar Blue per well. Plates were incubated for 5−6 h and measured for fluorescence at 530-nm excitation and 595-nm emission. The % inhibition was calculated using 0.4% DMSO (no inhibition) and 5 μM puromycin (100% inhibition) data. IC_50_ values were obtained by plotting % inhibition against log dose using GraphPad Prism v.6 (San Diego, CA, USA) nonlinear regression with a variable slope plot.

### 3.8. Biological Data Analysis

Normalizing of raw data used the in-plate positive (5 μM puromycin 100% inhibition) and negative (0.4% DMSO–no inhibition) controls to obtain percent inhibition and then used to calculate IC_50_ values through a 4 parameter logistic curve fitting in GraphPad Prism v.6.

## 4. Conclusions

In summary, LSF using both photoredox and Diversinate^™^ chemistry on the OSM triazolopyrazine Series 4 scaffold resulted in the generation of 12 new analogues, which were fully characterized using spectrometric and spectroscopic data analysis. A number of minor and unexpected side products were generated during these studies, including two molecules resulting from a proposed disproportionation reaction. In vitro screening using both 3D7 and Dd2 strains of *Plasmodium falciparum* identified five compounds with moderate antimalarial activity (IC_50_ < 9 µM), all of which showed encouraging selectivity profiles as they displayed no toxicity against HEK293 cells at 80 µM. Collectively, these data indicate that this particular OSM series warrant further medicinal chemistry studies and that LSF is one strategy for generating new and lead-like antimalarial compounds.

## Figures and Tables

**Table 1:** NMR data for compound **5** in DMSO-*d_6_* ^a^

Position	δ_H_, mult., J in Hz, int.	δ_C_, mult., J in Hz	COSY	HMBC	ROESY
3		145.6, s			
5		143.9, s			
6	7.59, s, 1 H	109.1, s		5, 8	21
8	9.01, s, 1 H	134.7, s		6, 9	
9		147.4, s			
10		124.8, s			
11	7.81, m, 1 H	132.8, s	12	3, 13,15	12
12	7.31, m, 1 H	117.6, s	11	10,14	11
13		151.9, t, 3.3			
14	7.31, m, 1 H	117.6, s	15	10, 12	15
15	7.81, m, 1 H	132.8, s	14	3, 11, 13	14
17	7.36, t, 73.6, 1 H	116.1, t, 258.5		13	
21	4.33, m, 2 H	69.4, s	22	5, 22	6, 22
22	0.91, m, 2 H	16.5, s	21	21, 24, 25, 26	21, 24, 25, 26
24	0.07, s, 3 H	−1.7, s		22, 25, 26	22
25	0.07, s, 3 H	−1.7, s		22, 24, 26	22
26	0.07, s, 3 H	−1.7, s		22, 24, 25	22

^a^ Recorded at 500 MHz (^1^H NMR) and 125 MHz (^13^C NMR) at 25 °C.

**Table 2:** NMR data for compound **13** in DMSO-*d_6_* ^a.^

Position	^1^_H_, mult., *J* in Hz, int	^13^_C_, mult., *J* in Hz	COSY	HMBC	ROESY
3		146.3, s			
5		142.8, s			
6	7.39, s, 1 H	107.9, s		5, 8	
8		143.2, s			
9		146.8, s			
10		124.8, s			
11	7.74, m, 1 H	132.6, s	12	3, 13,15	12
12	7.29, m, 1 H	117.6, s	11	10,14	11
13		152.0, t, 3.2			
14	7.29, m, 1 H	117.6, s	15	10, 12	15
15	7.74, m, 1 H	132.6, s	14	3, 11, 13	14
17	7.36, t, 73.7, 1 H	116.2, t, 258.2		13	
21	4.42, t, 6.5, 2 H	71.0, s	22	5, 22, 23	6, 22
22	2.86, t, 6.5, 2 H	33.9, s	21	21, 23, 24, 28	21, 24, 28
23		137.4, s			
24	6.90, m, 1 H	128.7, s	25	22, 26, 28	25
25 26 27 28 29	7.17, m, 1 H 7.16, m, 1 H 7.17, m, 1 H 6.90, m, 1 H 2.72, s, 3 H	128.2, s 126.4, s 128.2, s 128.7, s 19.7, s	24, 26 25, 27 26, 28 27	23, 27 24, 28 23, 25 22, 24, 26 6, 8, 9	24, 26 25, 27 26, 28 27

^a^ Recorded at 500 MHz (^1^H NMR) and 125 MHz (^13^C NMR) at 25 °C.

**Table 3:** Biological data for triazolopyrazines **1**–**18**.

Compounds	*Pf* 3D7 ^a^ IC_50_ ± SD µM	*Pf* Dd2 ^b^ IC_50_ ± SD µM	SI for 3D7 ^c^	SI for Dd2 ^c^
1	16.81 ± 1.25 ^d^	22.81 ± 13.73	>4	>3
2	0.31 ± 0.01	0.54 ± 0.02	>258	>148
3	16.65 ± 1.02	21.24 ± 5.41	>4	>3
4	^e^	NA	-	-
5	13.86 ± 0.87	NA	>5	-
6	^e^	NA	-	-
7	12.62 ± 1.90	14.32 ± 0.62	>6	>5
8	19.18 ± 0.28 ^d^	32.39 ± 1.44	>4	>2
9	18.89 ± 0.66 ^d^	25.87 ± 0.74	>4	>3
10	27.33 ± 0.82 ^d^	38.25 ± 0.61	>2	>2
11	12.49 ± 0.58	31.22 ± 0.82	>6	>2
12	27.33 ± 0.82 ^d^	^e^	>2	
13	11.01 ± 0.56 ^d^	8.20 ± 0.60	>7	>9
14	4.84 ± 0.13	6.18 ± 0.09	>16	>12
15	1.72 ± 0.02	2.58 ± 0.07	>46	>31
16	6.40 ± 0.10	24.70 ± 0.15	>12	>3
17	20.19 ± 2.86 ^d^	^e^	>3	
18	^e^	NA	-	-
**Controls**	***Pf* 3D7** **^a^** **IC_50_ ± SD nM**	***Pf* Dd2** **^b^** **IC_50_ ± SD nM**	**SI for 3D7** **^c^**	**SI for Dd2** **^c^**
Pyrimethamine	12.08 ± 0.04	^e^	>331	-
Artesunate	3.48 ± 0.14	2.00 ± 0.10	>2874	>5000
Puromycin	95.28 ± 1.45	101.00 ± 2.80	12	11

^a^ 3D7 = *P. falciparum* (chloroquine-sensitive strain). ^b^ Dd2 = *P. falciparum* (chloroquine, pyrimethamine, and mefloquine drug-resistant strain). ^c^ All compounds **1**–**18** and controls were tested for cytotoxicity against human embryonic kidney cells (HEK293) in order to determine selectivity index (SI) using the formula: SI = HEK293 IC_50_/parasite IC_50_. All compounds were inactive at the top dose of 80 µM. ^d^ Estimated IC_50_ as only ~90% inhibition reached at the top dose of 80 *µ*M. ^e^ There was <41% inhibition observed at the top dose of 80 µM. NA = not active (at the top dose of 80 µM). SD = standard deviation.

## Data Availability

The data presented in this study are contained within the article and Supplementary Material.

## Supplementary Materials

The following are available: 1D/2D NMR and HRMS spectra for compounds **2**–**6** and **8**–**18** and NMR data tables for **2**–**6** and **8**–**18**.

## Sample Availability

Samples of the compounds are available from the authors.
