# Supplementary material for: Synthesis of New Triazolopyrazine Antimalarial Compounds
Source: Molecules. 2021 Apr 21;26(9):2421. doi: 10.3390/molecules26092421 (PMC8122397; doi:10.3390/molecules26092421)
Supplement: Supplementary file 1 [file molecules-26-02421-s001.zip › molecules-1186402-supplementary.pdf]

Synthesis of New Triazolopyrazine Antimalarial Compounds

Daniel J. G. Johnson [1], Ian D. Jenkins [1], Cohan Huxley [1], Mark J. Coster [1], Kah Yean Lum [1,2], Jonathan M. White [3], Vicky M. Avery [1,4], and Rohan A. Davis [1,2,*]

[1]Griffith Institute for Drug Discovery, School of Environment and Science, Griffith University, Brisbane, QLD 4111, Australia; d.johnson@griffithuni.edu.au (D. J. G. J.); i.jenkins@griffith.edu.au (I.D.J.); c.huxley@griffithuni.edu.au (C.H.); m.coster@griffith.edu.au (M.J.C.); k.lum@griffith.edu.au (K.Y.L.); v.avery@griffith.edu.au (V.M.A.)

[2]NatureBank, Griffith University, Brisbane, QLD 4111, Australia

[3]School of Chemistry and Bio21 Institute, The University of Melbourne, Melbourne, VIC 3010, Australia; whitejm@unimelb.edu.au (J.M.W.)

[4]Discovery Biology, Griffith University, Brisbane, QLD 4111, Australia

*Correspondence: r.davis@griffith.edu.au; Tel: +61-7-3735-6043

Contents

S1: NMR data of compound **2** in DMSO-d_6

S2: ^{1}H NMR spectrum of compound **2** in DMSO-d_6

S3: ^{13}C NMR spectrum of compound **2** in DMSO-d_6

S4: COSY spectrum of compound **2** in DMSO-d_6

S5: HSQC spectrum of compound **2** in DMSO-d_6

S6: HMBC spectrum of compound **2** in DMSO-d_6

S7: ROESY spectrum of compound **2** in DMSO-d_6

S8: HRMS of compound **2**

S9: NMR data of compound **3** in DMSO-d_6

S10: ^{1}H NMR spectrum of compound **3** in DMSO-d_6

S11: ^{13}C NMR spectrum of compound **3** in DMSO-d_6

S12: COSY spectrum of compound **3** in DMSO-d_6

S13: HSQC spectrum of compound **3** in DMSO-d_6

S14: HMBC spectrum of compound **3** in DMSO-d_6

S15: ROESY spectrum of compound **3** in DMSO-d_6

S16: HRMS of compound **3**

S17: NMR data of compound **4** in DMSO-d_6

S18: ^{1}H NMR spectrum of compound **4** in DMSO-d_6

S19: ^{13}C NMR spectrum of compound **4** in DMSO-d_6

S20: COSY spectrum of compound **4** in DMSO-d_6

S21: HSQC spectrum of compound **4** in DMSO-d_6

S22: HMBC spectrum of compound **4** in DMSO-d_6

S23: HRMS of compound **4**

S24: NMR data of compound **5** in DMSO-d_6

S25: ^{1}H NMR spectrum of compound **5** DMSO-d_6

S26: ^{13}C NMR spectrum of compound **5** in DMSO-d_6

S27: COSY spectrum of compound **5** in DMSO-d_6

S28: HSQC spectrum of compound **5** in DMSO-d_6

S29: HMBC spectrum of compound **5** in DMSO-d_6

S30: ROESY spectrum of compound **5** in DMSO-d_6

S31: HRMS of compound **5**

S32: NMR data of compound **6** in DMSO-d_6

S33: ^{1}H NMR spectrum of compound **6** in DMSO-d_6

S34: ^{13}C NMR spectrum of compound **6** in DMSO-d_6

S35: COSY spectrum of compound **6** in DMSO-d_6

S36: HSQC spectrum of compound **6** in DMSO-d_6

S37: HMBC spectrum of compound **6** in DMSO-d_6

S38: HRMS of compound **6**

S39: NMR data of compound **8** in DMSO-d_6

S40: ^{1}H NMR spectrum of compound **8** in DMSO-d_6

S41: ^{13}C NMR spectrum of compound **8** in DMSO-d_6

S42: COSY spectrum of compound **8** in DMSO-d_6

S43: HSQC spectrum of compound **8** in DMSO-d_6

S44: HMBC spectrum of compound **8** in DMSO-d_6

S45: HRMS of compound **8**

S46: NMR data of compound **10** in DMSO-d_6

S47: ^{1}H NMR spectrum of compound **10** in DMSO-d_6

S48: ^{13}C NMR spectrum of compound **10** in DMSO-d_6

S49: COSY spectrum of compound **10** in DMSO-d_6

S50: HSQC spectrum of compound **10** in DMSO-d_6

S51: HMBC spectrum of compound **10** in DMSO-d_6

S52: HRMS of compound **10**

S53: NMR data of compound **11** in DMSO-d_6

S54: ^{1}H NMR spectrum of compound **11** in DMSO-d_6

S55: ^{13}C NMR spectrum of compound **11** in DMSO-d_6

S56: COSY spectrum of compound **11** in DMSO-d_6

S57: HSQC spectrum of compound **11** in DMSO-d_6

S58: HMBC spectrum of compound **11** in DMSO-d_6

S59: HRMS of compound **11**

S60: NMR data of compound **12** in DMSO-d_6

S61: ^{1}H NMR spectrum of compound **12** in DMSO-d_6

S62: ^{13}C NMR spectrum of compound **12** in DMSO-d_6

S63: COSY spectrum of compound **12** in DMSO-d_6

S64: HSQC spectrum of compound **12** in DMSO-d_6

S65: HMBC spectrum of compound **12** in DMSO-d_6

S66: ROESY spectrum of compound **12** in DMSO-d_6

S67: HRMS of compound **12**

S68: NMR data for compound **13** in DMSO-d_6

S69: ^{1}H NMR spectrum of compound **13** in DMSO-d_6

S70: ^{13}C NMR spectrum of compound **13** in DMSO-d_6

S71: COSY spectrum of compound **13** in DMSO-d_6

S72: HSQC spectrum of compound **13** in DMSO-d_6

S73: HMBC spectrum of compound **13** in DMSO-d_6

S74: ROESY spectrum of compound **13** in DMSO-d_6

S75: HRMS of compound **13**

S76: NMR data of compound **14** in DMSO-d_6
S77: ^{1}H NMR Spectrum of compound **14** in DMSO-d_6
S78: ^{13}C NMR spectrum of compound **14** in DMSO-d_6
S79: COSY spectrum of compound **14** in DMSO-d_6
S80: HSQC spectrum of compound **14** in DMSO-d_6
S81: HMBC spectrum of compound **14** in DMSO-d_6
S82: HRMS of compound **14**
S83: NMR data of compound **15** in DMSO-d_6
S84: ^{1}H NMR spectrum of compound **15** in DMSO-d_6
S85: ^{13}C NMR spectrum of compound **15** in DMSO-d_6
S86: COSY spectrum of compound **15** in DMSO-d_6
S87: HSQC spectrum of compound **15** in DMSO-d_6
S88: HMBC spectrum of compound **15** in DMSO-d_6
S89: HRMS of compound **15**
S90: NMR data of compound **16** in DMSO-d_6
S91: ^{1}H NMR Spectrum of compound **16** in DMSO-d_6
S92: ^{13}C NMR spectrum of compound **16** in DMSO-d_6
S93: COSY spectrum of compound **16** in DMSO-d_6
S94: HSQC spectrum of compound **16** in DMSO-d_6
S95: HMBC spectrum of compound **16** in DMSO-d_6
S96: HRMS of compound **16**
S97: NMR data of compound **17** in DMSO-d_6
S98: ^{1}H NMR Spectrum of compound **17** in DMSO-d_6
S99: ^{13}C NMR spectrum of compound **17** in DMSO-d_6
S100: COSY spectrum of compound **17** in DMSO-d_6
S101: HSQC spectrum of compound **17** in DMSO-d_6
S102: HMBC spectrum of compound **17** in DMSO-d_6
S103: HRMS of compound **17**
S104: NMR data of compound **18** in DMSO-d_6
S105: ^{1}H NMR spectrum of compound **18** in DMSO-d_6
S106: ^{13}C NMR spectrum of compound **18** in DMSO-d_6
S107: COSY spectrum of compound **18** in DMSO-d_6
S108: HSQC spectrum of compound **18** in DMSO-d_6
S109: HMBC spectrum of compound **18** in DMSO-d_6
S110: HRMS of compound **18**

S1: NMR data of compound **2** in DMSO-d_6[a]

Pos.	δH, mult. (*J* in Hz), int.	δC, mult. (*J* in Hz)	COSY	HMBC	ROESY
3		145.5, s			
5		143.8, s			
6	7.60, s, 1H	108.8, s		5, 8	21
8	9.04, s, 1H	135.0, s		6, 9	
9		147.4, s			
10		124.7, s			
11	7.77, m, 1H	132.6, s	12	3, 13, 15	12
12	7.30, m, 1H	117.6, s	11	10, 14	11
13		152.0, t (3.2)			
14	7.30, m, 1H	117.6, s	15	10, 12	15
15	7.77, m, 1H	132.6, s	14	3, 11, 13	14
17	7.36, t (73.7), 1H	116.2, t (258.8)		13	
21	4.50, t (6.5), 2H	71.2, s		5, 23	6, 22
22	2.90, t (6.5), 2H	33.8, s		24, 28	21, 24, 28
23		137.3, s			
24	6.91, m, 1H	128.3, s	25	22, 26, 28	22, 25
25	7.18, m, 1H	128.6, s	24	23, 27	24
26	7.16, m, 1H	126.3, s		24, 28	
27	7.18, m, 1H	128.6, s	28	23, 25	28
28	6.91, m, 1H	128.3, s	27	22, 24, 26	22, 27

[a] Recorded at 500 MHz (^{1}H NMR) and 125 MHz (^{13}C NMR) at 25 °C.

S2: ^{1}H NMR spectrum of compound **2** in DMSO-d_6

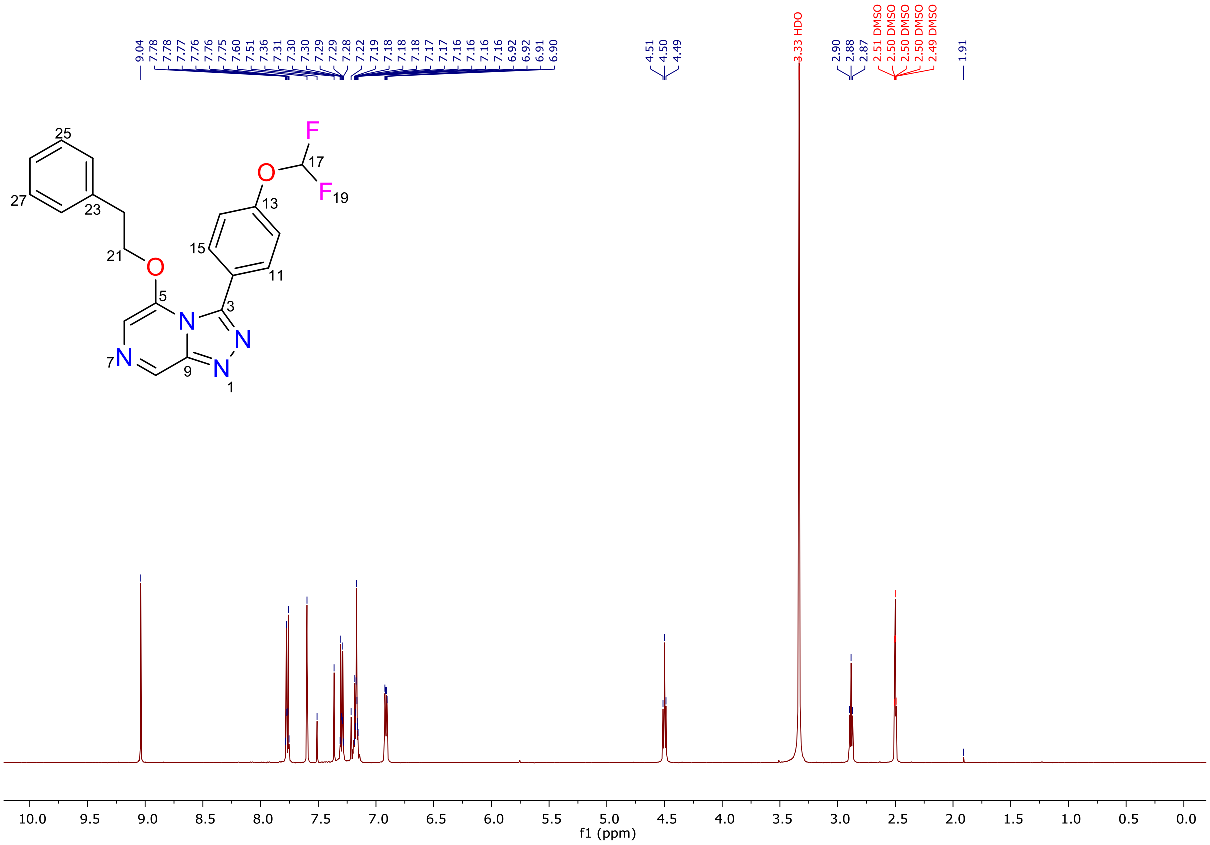

S3: ^{13}C NMR spectrum of compound **2** in DMSO-d_6

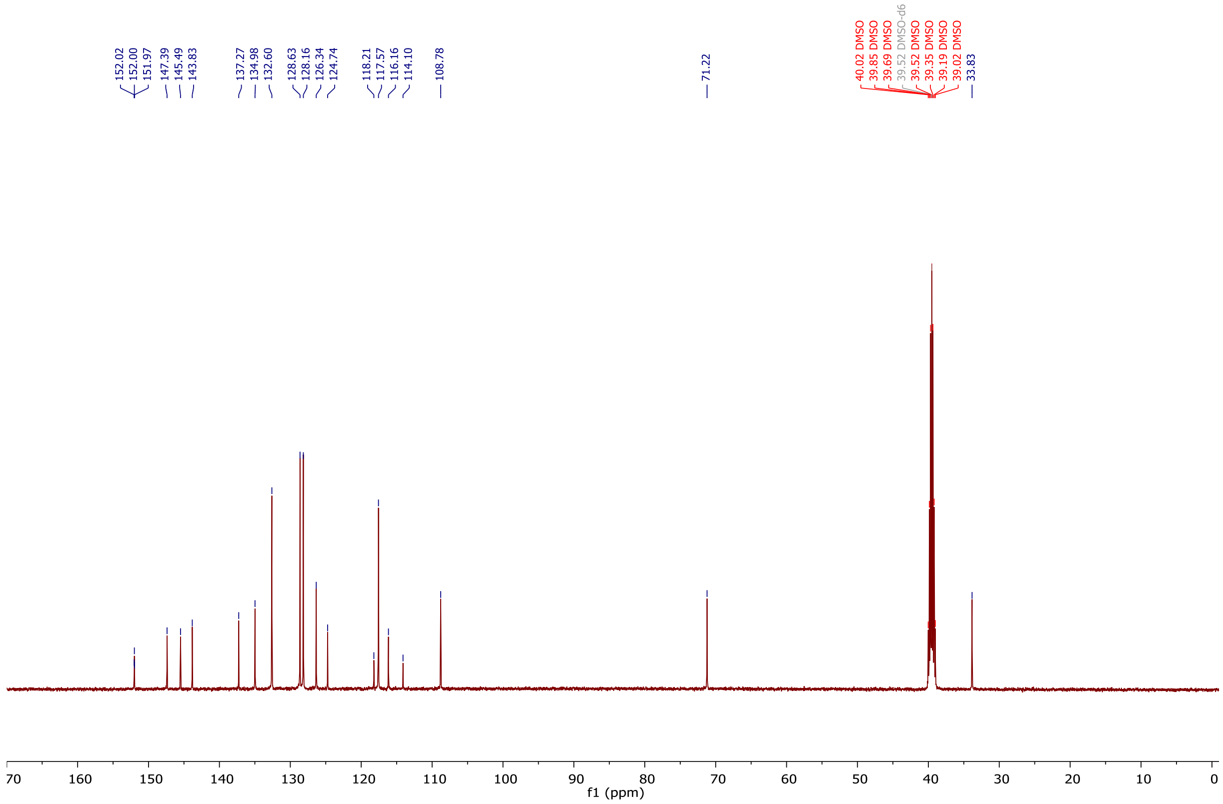

S4: COSY spectrum of compound **2** in DMSO-d_6

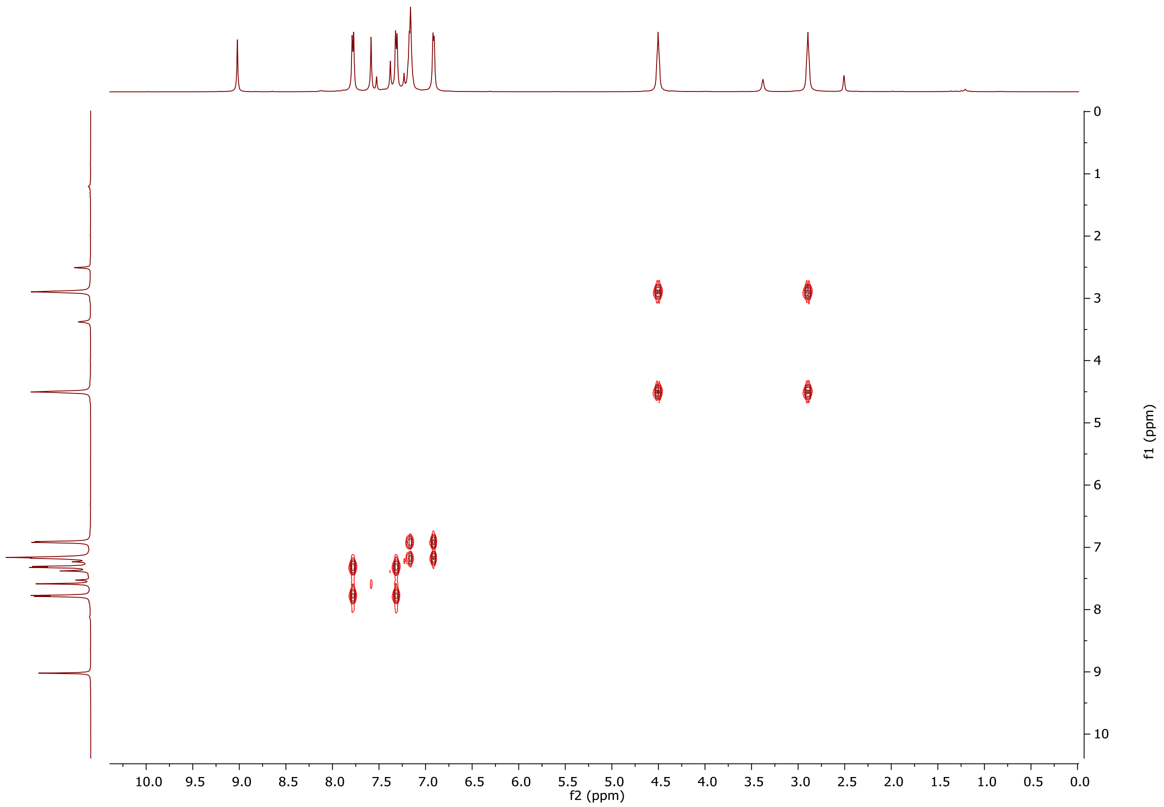

S5: HSQC spectrum of compound **2** in DMSO-d_6

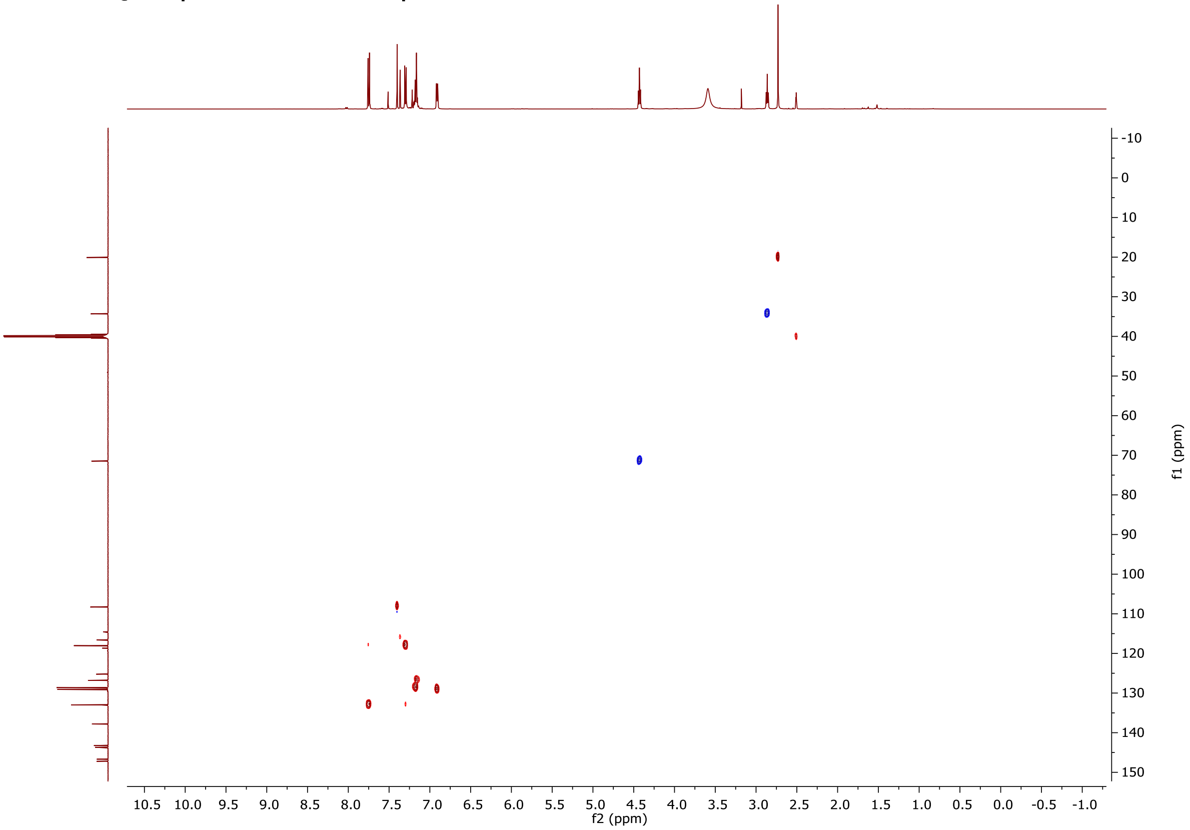

S6: HMBC spectrum of compound **2** in DMSO-d_6

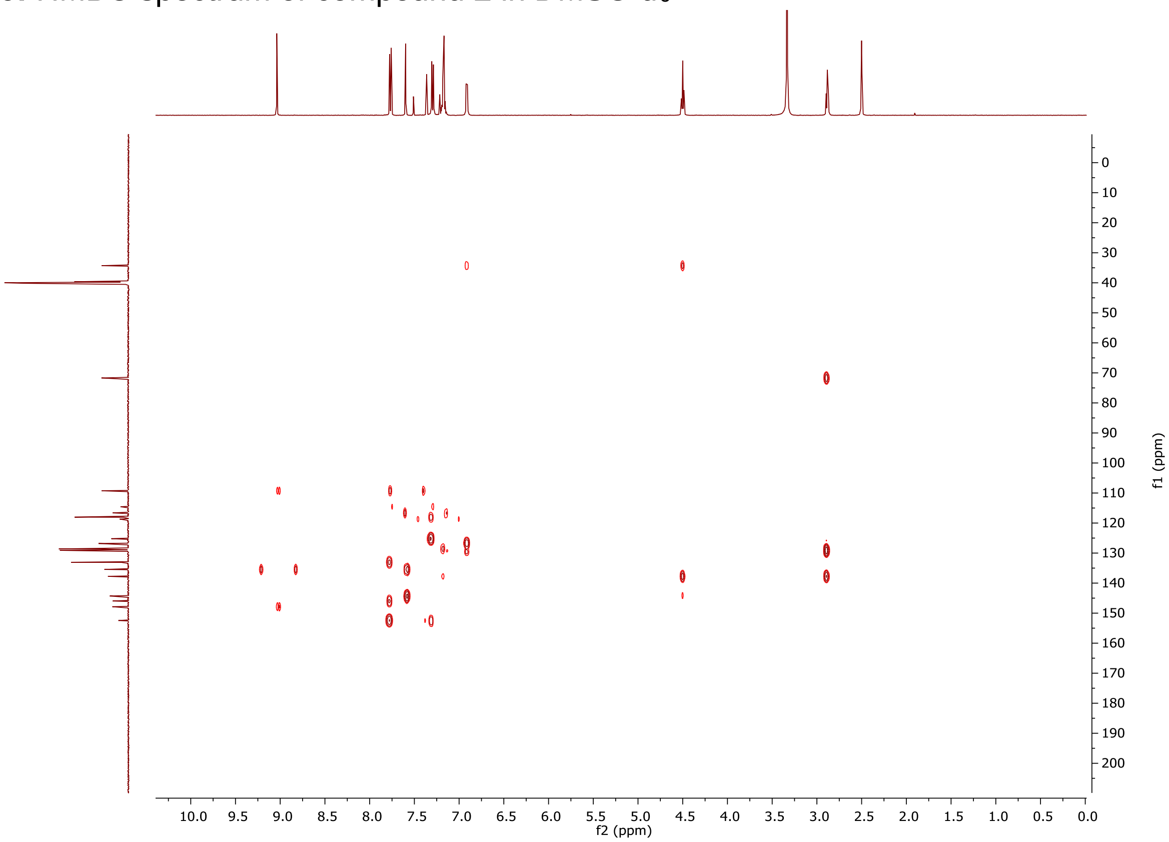

S7: ROESY spectrum of compound **2** in DMSO-d_6

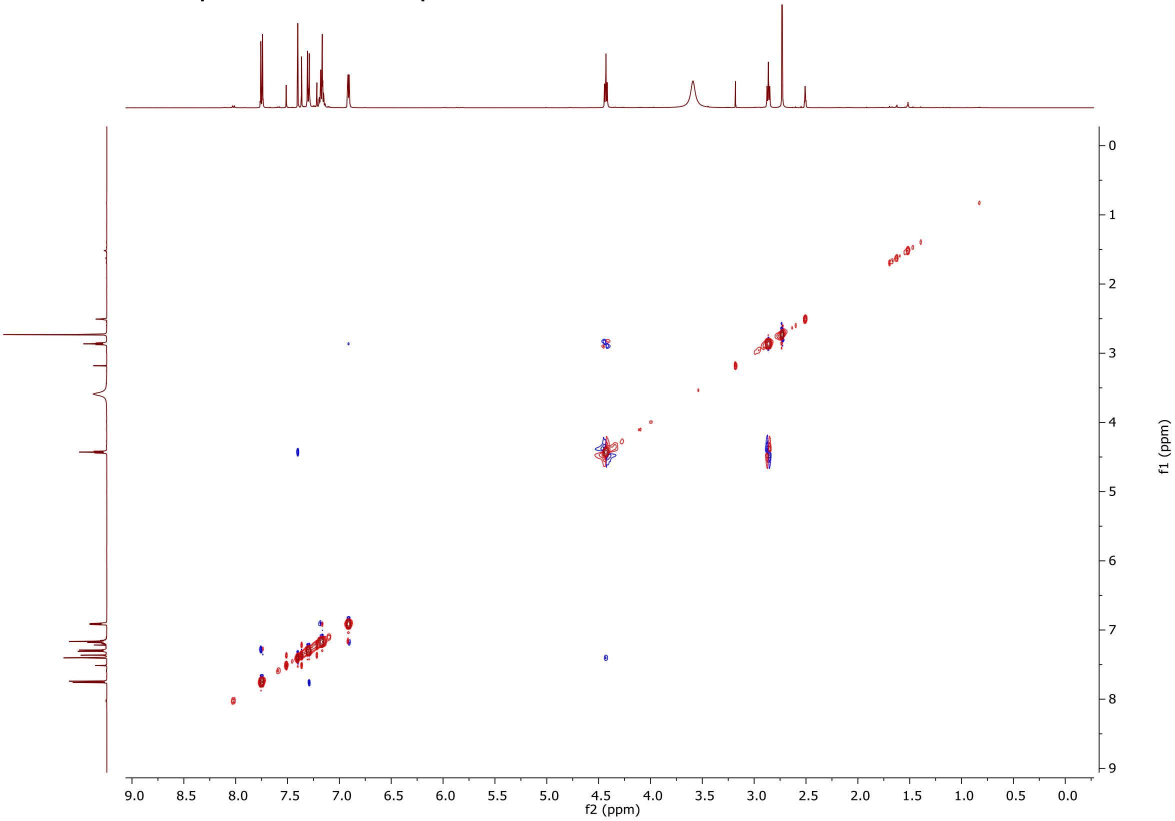

S8: HRMS of compound **2**

Griffith UNIVERSITY

High Resolution Mass Spectrum

Analysis Info

Analysis Name	D:\Data\KahYean\20200904\RAD815000001.d		
Method	DirectInfusion_2018_pos.m	Acquisition Date	9/18/2020 11:07:52 AM
Sample Name	RAD815	Instrument	maXis II ETD 1823391.22321
Comment			

+MS, 0.4-0.4min #21-22

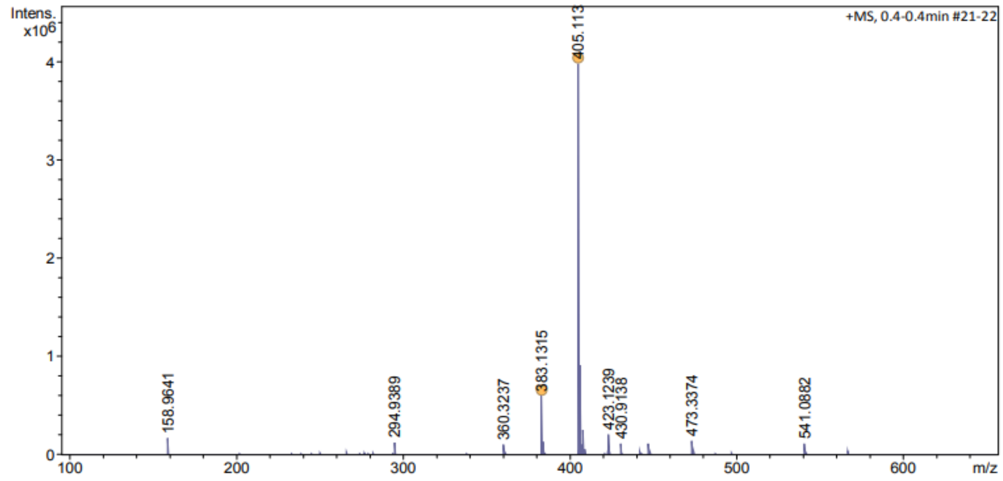

Meas. m/z	#	Ion Formula	m/z	err [ppm]	mSigma	# mSigma	Score	rdb	e^- Conf	N-Rule
383.1315	1	C20H17F2N4O2	383.1314	-0.1	9.5	1	100.00	13.5	even	ok
405.1133	1	C20H16F2N4NaO2	405.1134	0.1	3.2	1	100.00	13.5	even	ok

S9: NMR data of compound **3** in DMSO-d_6[a]

Pos.	δ_H, mult. (*J* in Hz), int.	δ_C, mult. (*J* in Hz)	COSY	HMBC	ROESY
3		145.7, s			
5		142.6, s			
6	7.69, s, 1H	109.7, s		5, 8	21
8	9.15, s, 1H	136.8, s			
9		147.4, s			
10		124.4, s			
11	7.79, m, 1H	132.4, s	12	3, 13,15	12
12	7.29, m, 1H	117.7, s	11	10,14	11
13		152.0, t (3.2)			
14	7.29, m, 1H	117.7, s	15	10, 12	15
15	7.79, m, 1H	132.4, s	14	3, 11, 13	14
17	7.35, t (73.8), 1H	116.1, t (258.8)		13	
21	5.05, q (8.5), 2H	66.1, q (36.2)		5, 22	6
22		122.6, q (277.2)			

[a] Recorded at 500 MHz (^{1}H NMR) and 125 MHz (^{13}C NMR) at 25 °C.

S10: ^{1}H NMR spectrum of compound **3** in DMSO-d_6

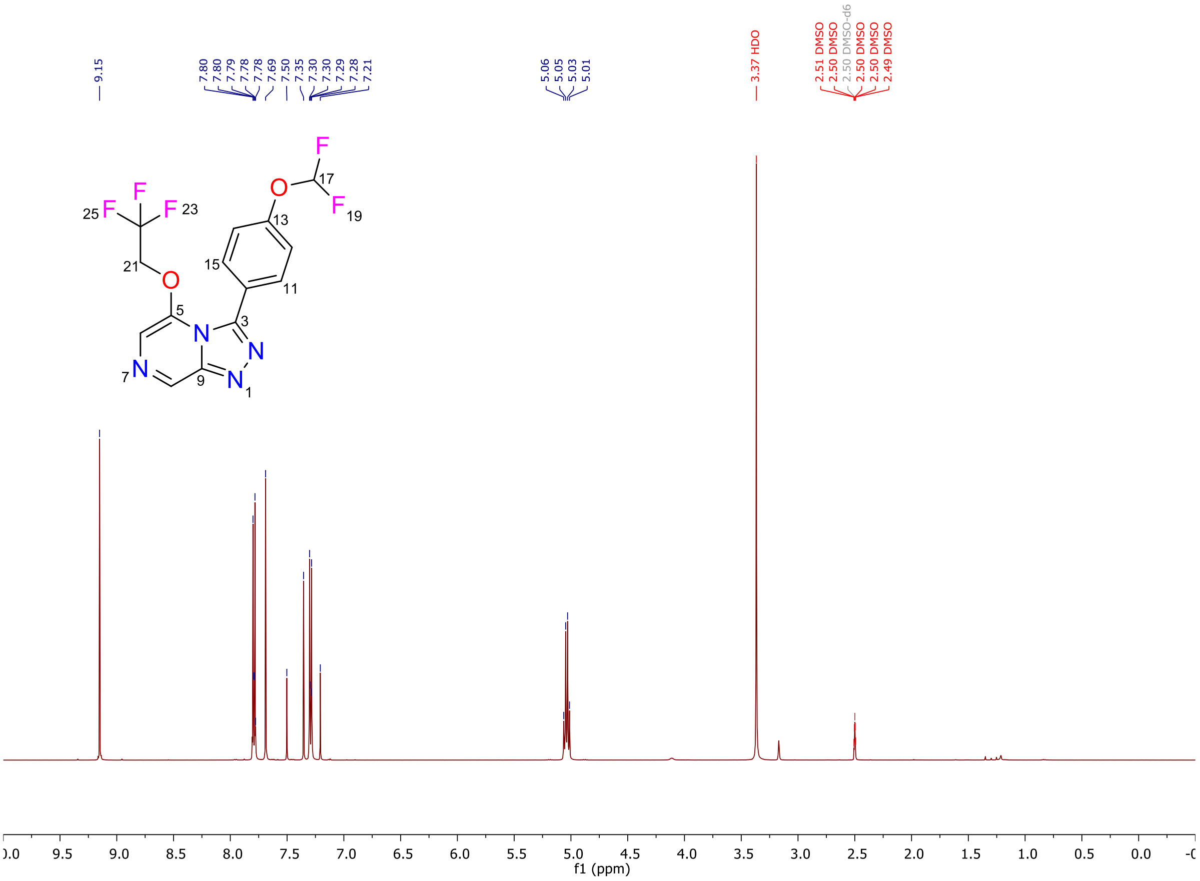

S11: ^{13}C NMR spectrum of compound **3** in DMSO-d_6

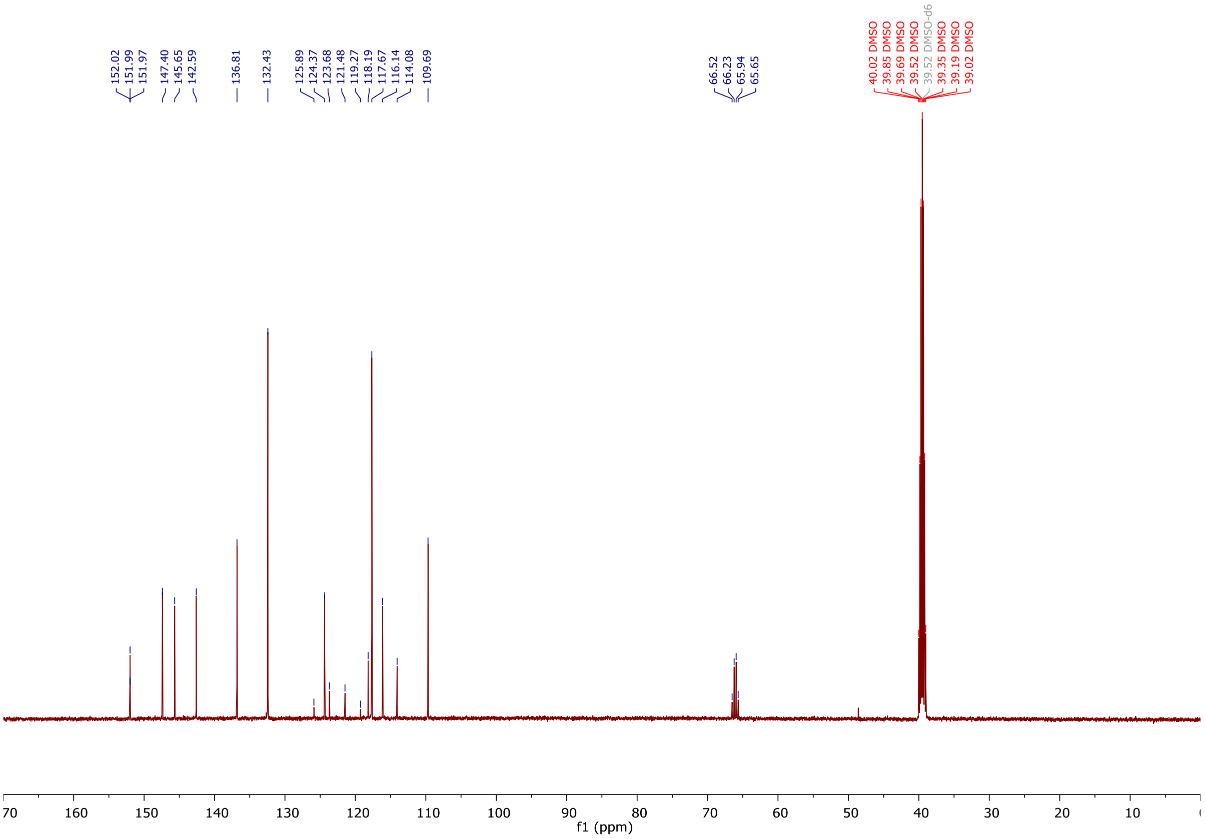

S12: COSY spectrum of compound **3** in DMSO-d_6

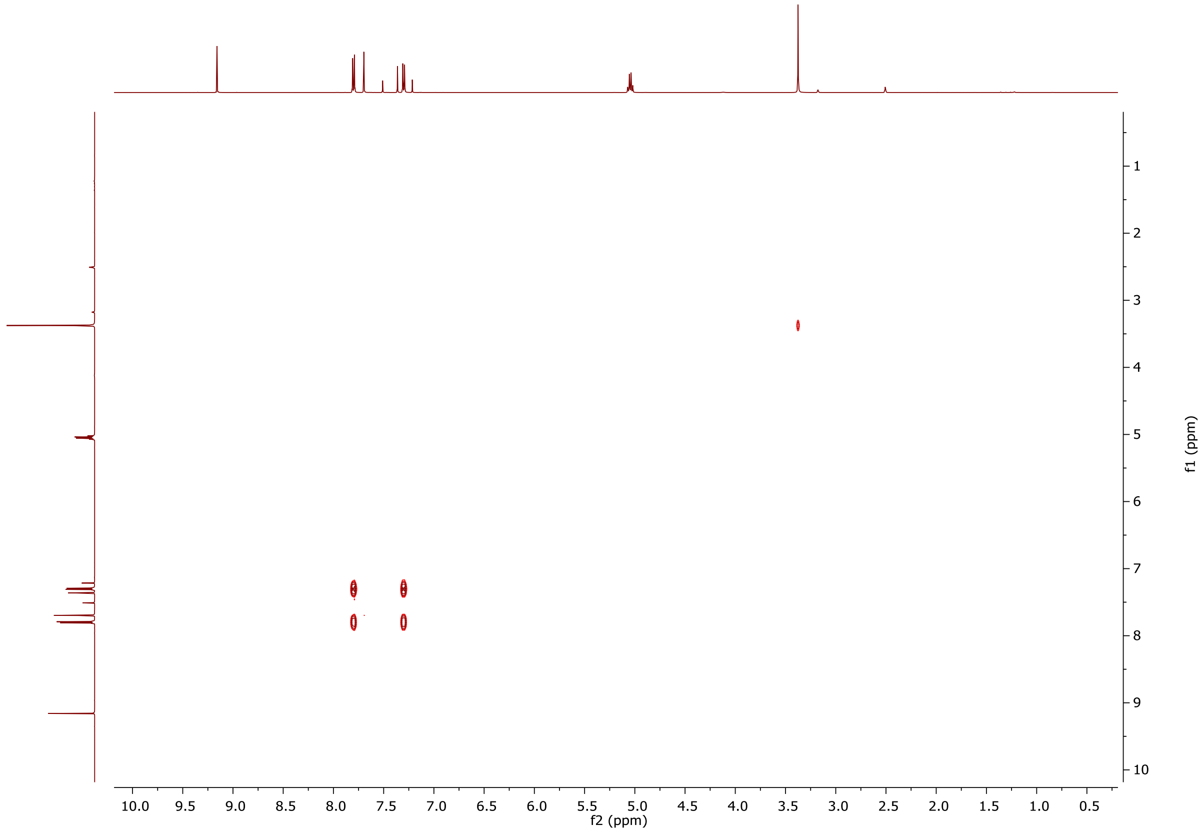

S13: HSQC spectrum of compound **3** in DMSO-d_6

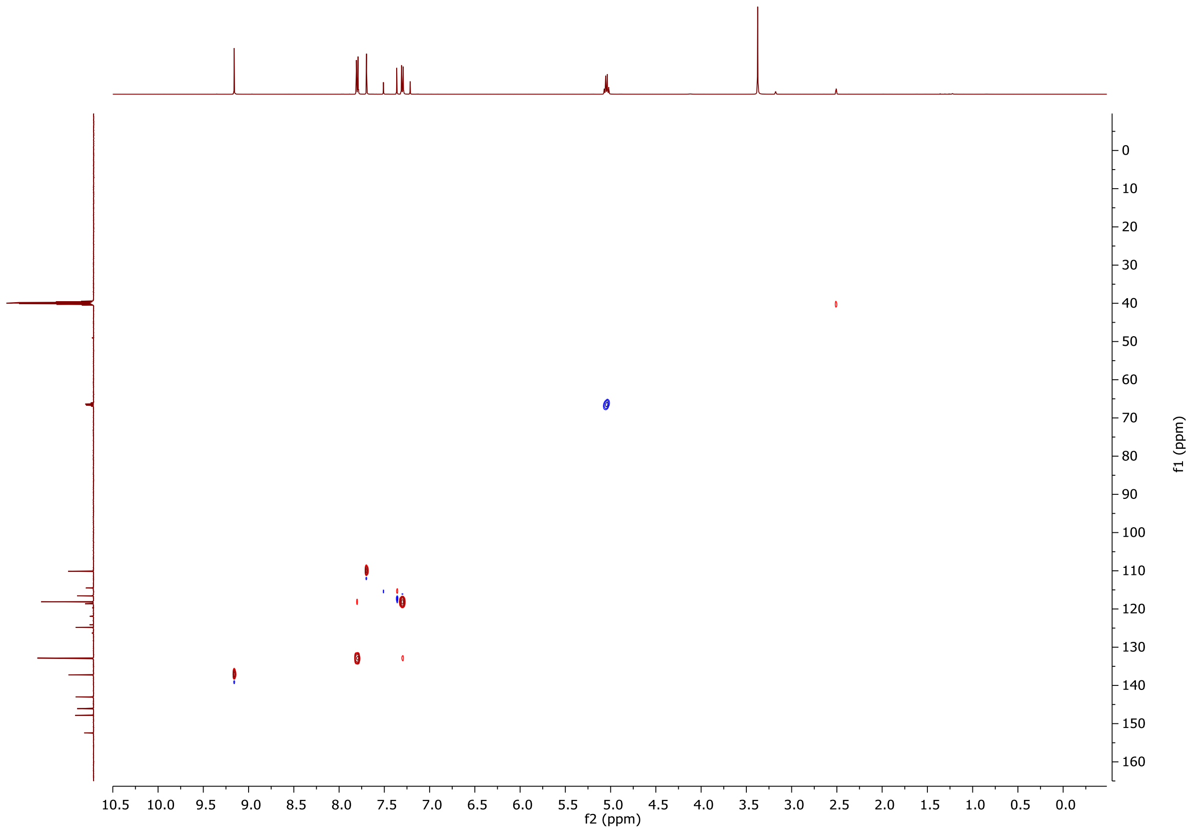

S14: HMBC spectrum of compound **3** in DMSO-d_6

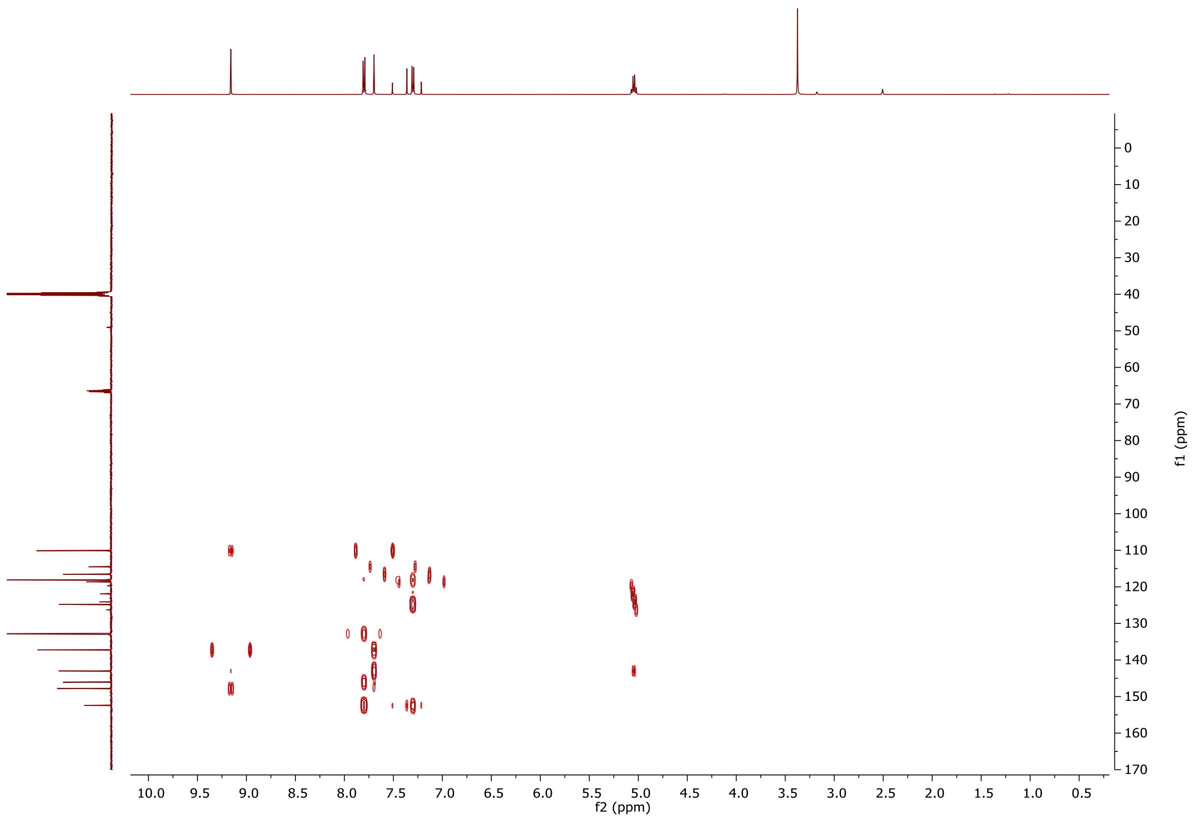

S15: ROESY spectrum of compound **3** in DMSO-d_6

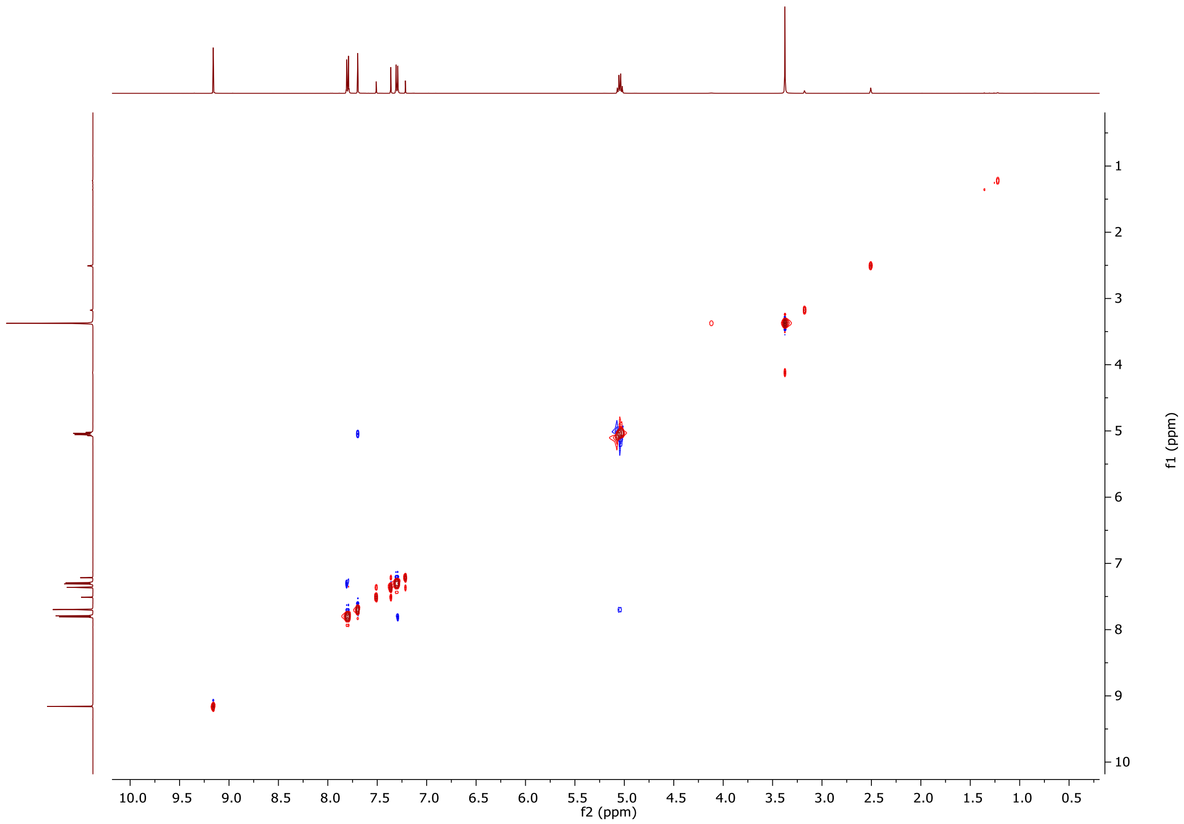

S16: HRMS of compound **3**

Griffith UNIVERSITY

High Resolution Mass Spectrum

Analysis Info

Analysis Name	D:\Data\KahYean\20200508\RAD863000002.d		
Method	DirectInfusion_2018_pos.m	Acquisition Date	5/12/2020 12:03:48 PM
Sample Name	RAD863	Instrument	maXis II ETD 1823391.22321
Comment			

+MS, 0.1min #8

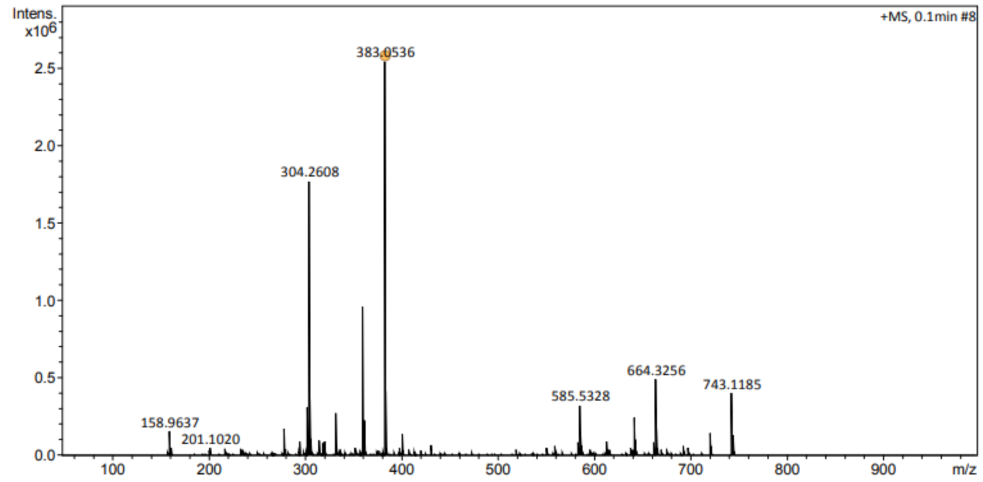

Meas. m/z	#	Ion Formula	m/z	err [ppm]	mSigma	# mSigma	Score	rdb	e⁻ Conf	N-Rule
383.0536	1	C14H9F5N4NaO2	383.0538	0.4	2.2	1	100.00	9.5	even	ok

S17: NMR data of compound **4** in DMSO-d_6[a]

Pos	δ_H, mult. (*J* in Hz), int.	δ_C, mult. (*J* in Hz)	COSY	HMBC
3		148.6, s		
5	7.43, d (5.8), 1H	103.9, s	6	6, 9
6	6.92, d (5.8), 1H	118.7, s	5	5, 8
8		153.0, s		
9		145.3, s		
10		122.6, s		
11	7.91, m, 1H	130.4, s	12	3, 13,15
12	7.42, m, 1H	119.2, s	11	10,14
13		152.5, t (3.3)		
14	7.42, m, 1H	119.2, s	15	10, 12
15	7.91, m, 1H	130.4, s	14	3, 11, 13
17	7.40, t (73.6), 1H	116.1, t (258.7)		13
20-OH	11.48, br s, 1H			

[a] Recorded at 500 MHz (^1H NMR) and 125 MHz (^{13}C NMR) at 25 °C.

S18: ^1H NMR spectrum of compound **4** in DMSO-d_6

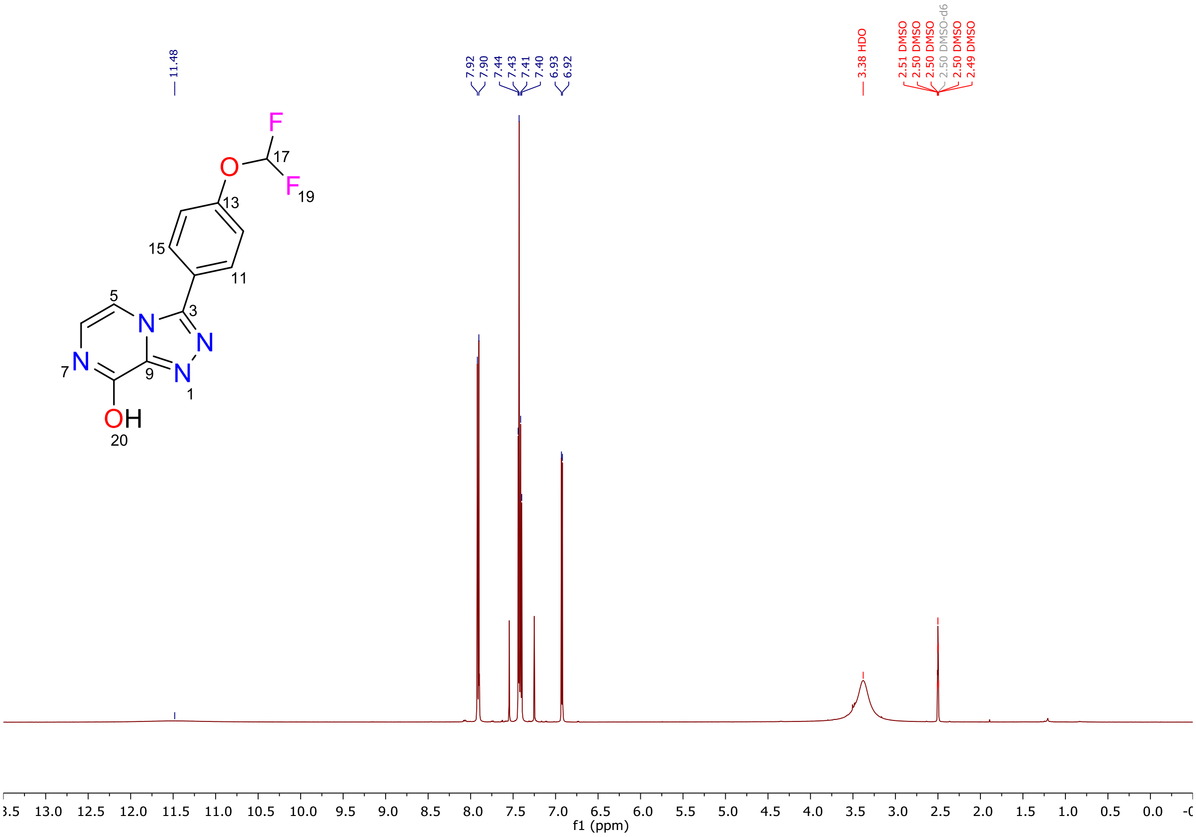

S19: ^{13}C NMR spectrum of compound **4** in DMSO-d_6

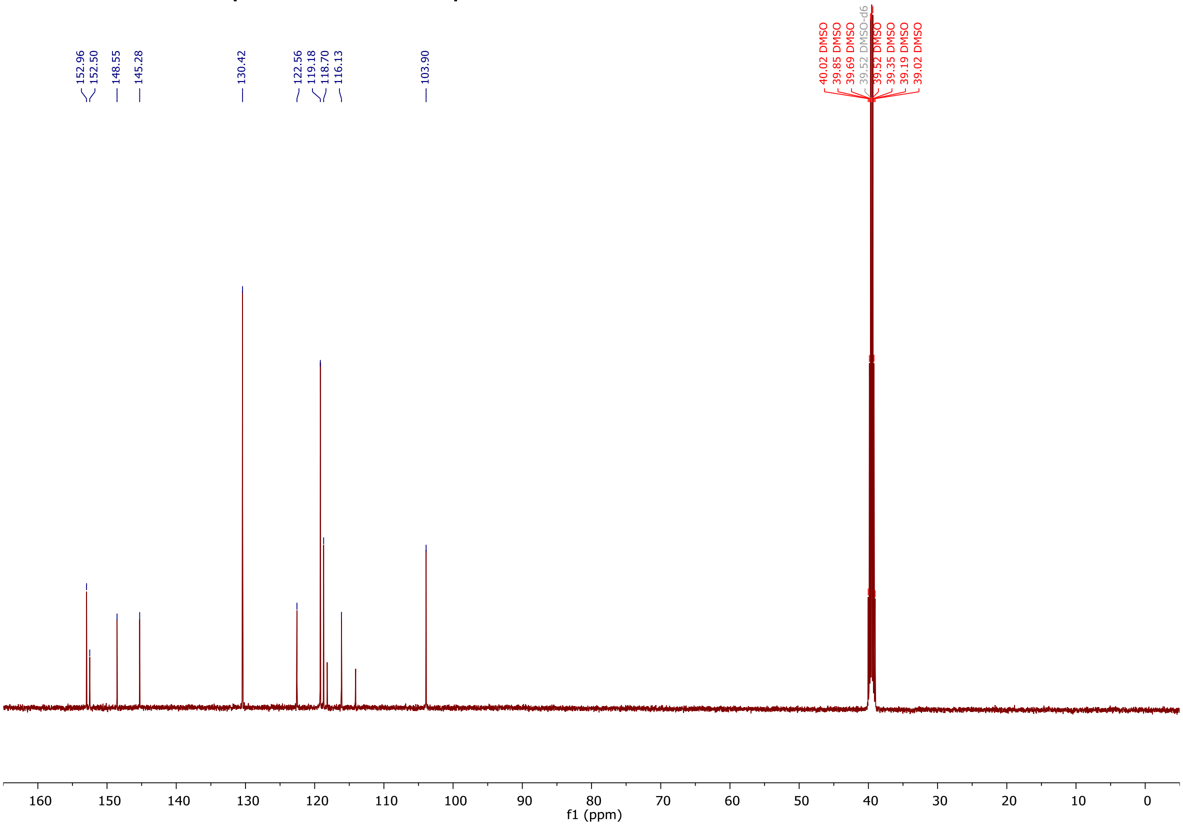

S20: COSY spectrum of compound **4** in DMSO-d_6

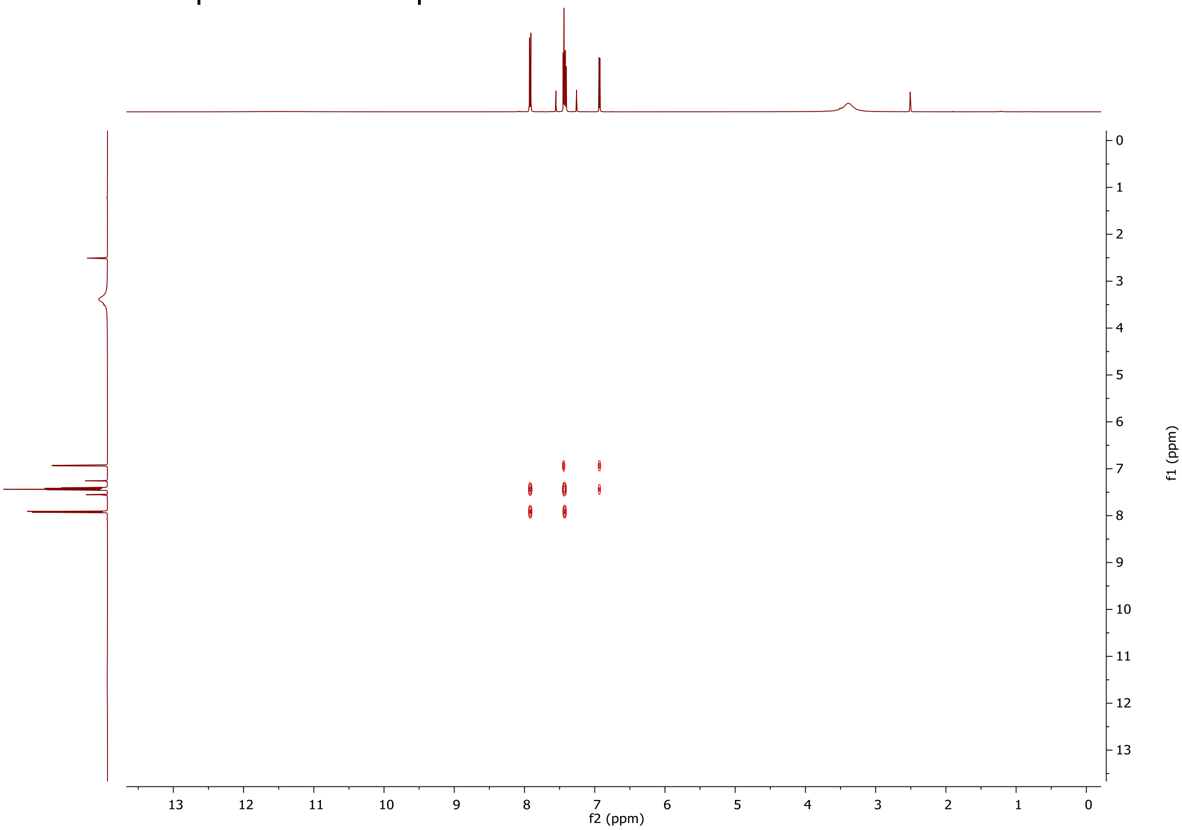

S21: HSQC spectrum of compound **4** in DMSO-d_6

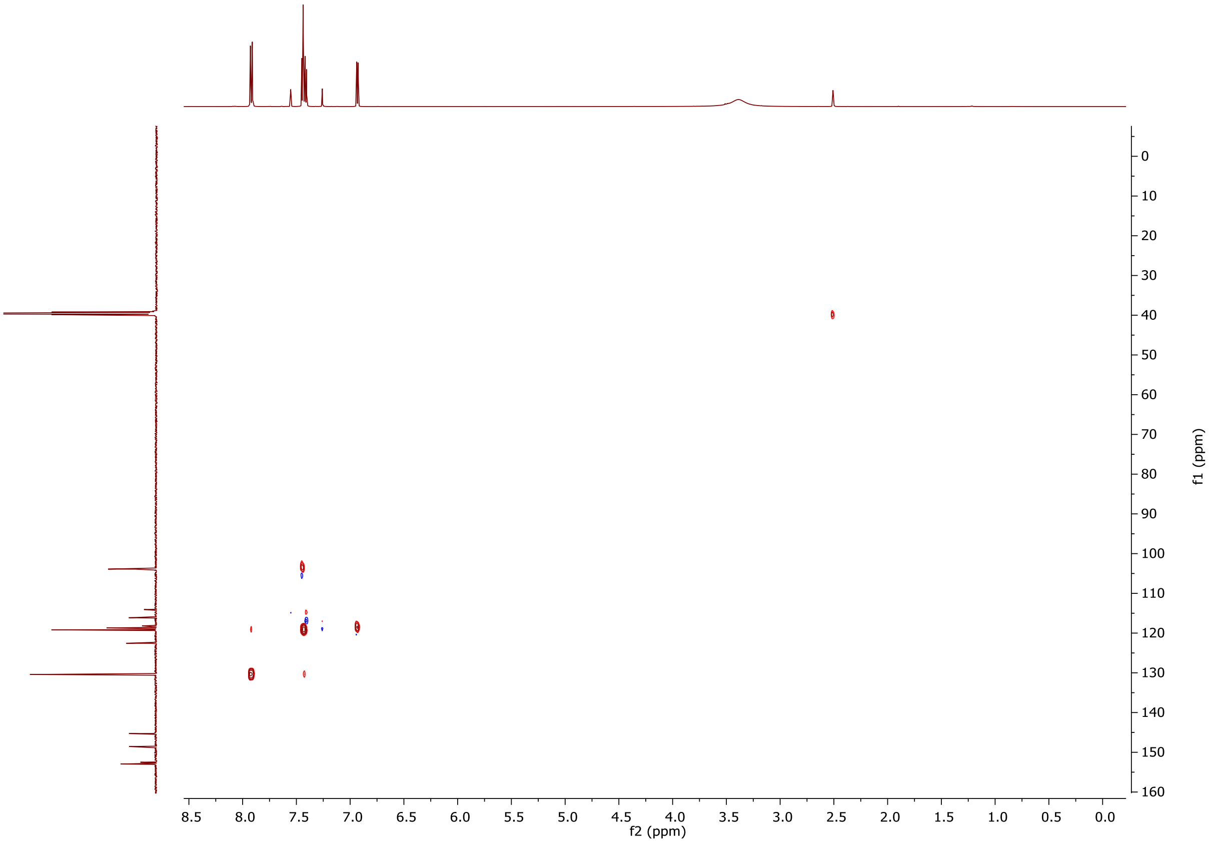

S22: HMBC spectrum of compound **4** in DMSO-d_6

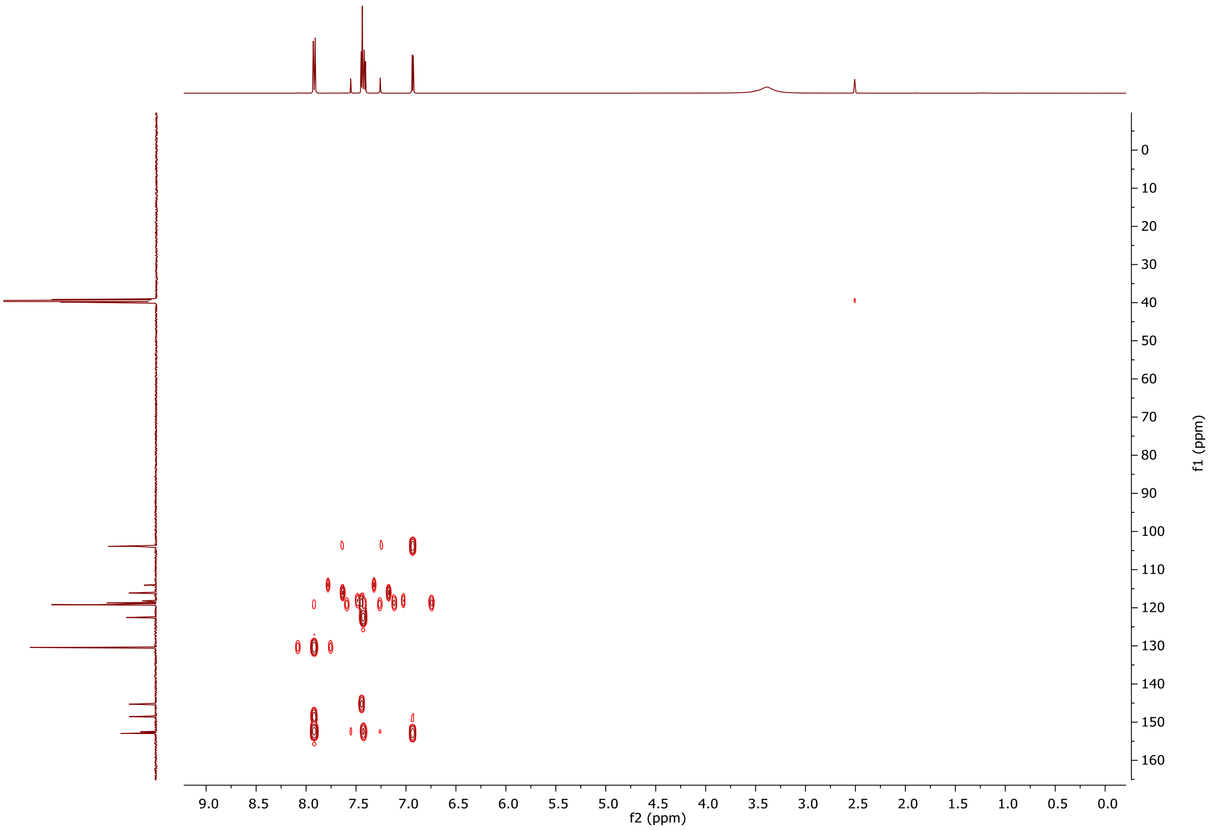

S23: HRMS of compound **4**

High Resolution Mass Spectrum

Analysis Info

Analysis Name	D:\Data\KahYean\20200508\RAD862000002.d		
Method	DirectInfusion_2018_pos.m	Acquisition Date	5/12/2020 11:36:43 AM
Sample Name	RAD862	Instrument	maXis II ETD 1823391.22321
Comment			

+MS, 0.1-0.1min #7-8

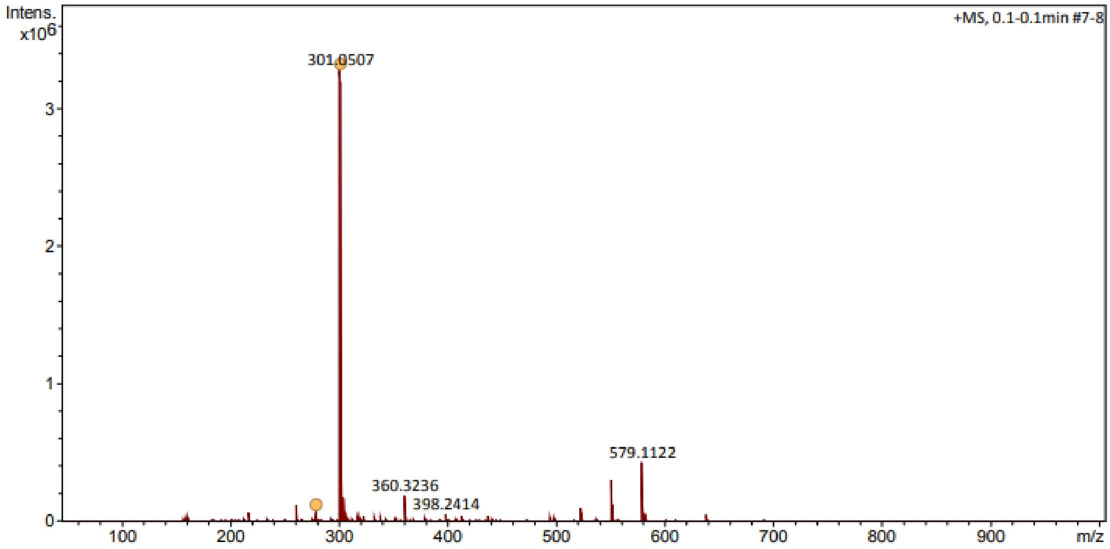

Meas. m/z	#	Ion Formula	m/z	err [ppm]	mSigma	# mSigma	Score	rdb	e⁻ Conf	N-Rule
279.0688	1	C12H9F2N4O2	279.0688	0.2	10.8	1	100.00	9.5	even	ok
301.0507	1	C12H8F2N4NaO2	301.0508	0.3	5.9	1	100.00	9.5	even	ok

S24: NMR data of compound **5** in DMSO-d_6[a]

Pos.	δ_H, mult. (*J* in Hz), int.	δ_C, mult. (*J* in Hz)	COSY	HMBC	ROESY
3		145.6, s			
5		143.9, s			
6	7.59, s, 1H	109.1, s		5, 8	21
8	9.01, s, 1H	134.7, s		6, 9	
9		147.5, s			
10		124.8, s			
11	7.81, m, 1H	132.8, s	12	3, 13,15	12
12	7.31, m, 1H	117.6, s	11	10,14	11
13		151.9, t (3.3)			
14	7.31, m, 1H	117.6, s	15	10, 12	15
15	7.81, m, 1H	132.8, s	14	3, 11, 13	14
17	7.36, t (73.6), 1H	116.1, t, (258.5)		13	
21	4.33, m, 2H	69.4, s	22	5, 22	6, 22
22	0.91, m, 2H	16.5, s	21	21, 24, 25, 26	21, 24, 25, 26
24	0.07, s, 3H	-1.7, s		22, 25, 26	22
25	0.07, s, 3H	-1.7, s		22, 24, 26	22
26	0.07, s, 3H	-1.7, s		22, 24, 25	22

[a] Recorded at 500 MHz (^{1}H NMR) and 125 MHz (^{13}C NMR) at 25 °C.

S25: ^{1}H NMR spectrum of compound **5** DMSO-d_6

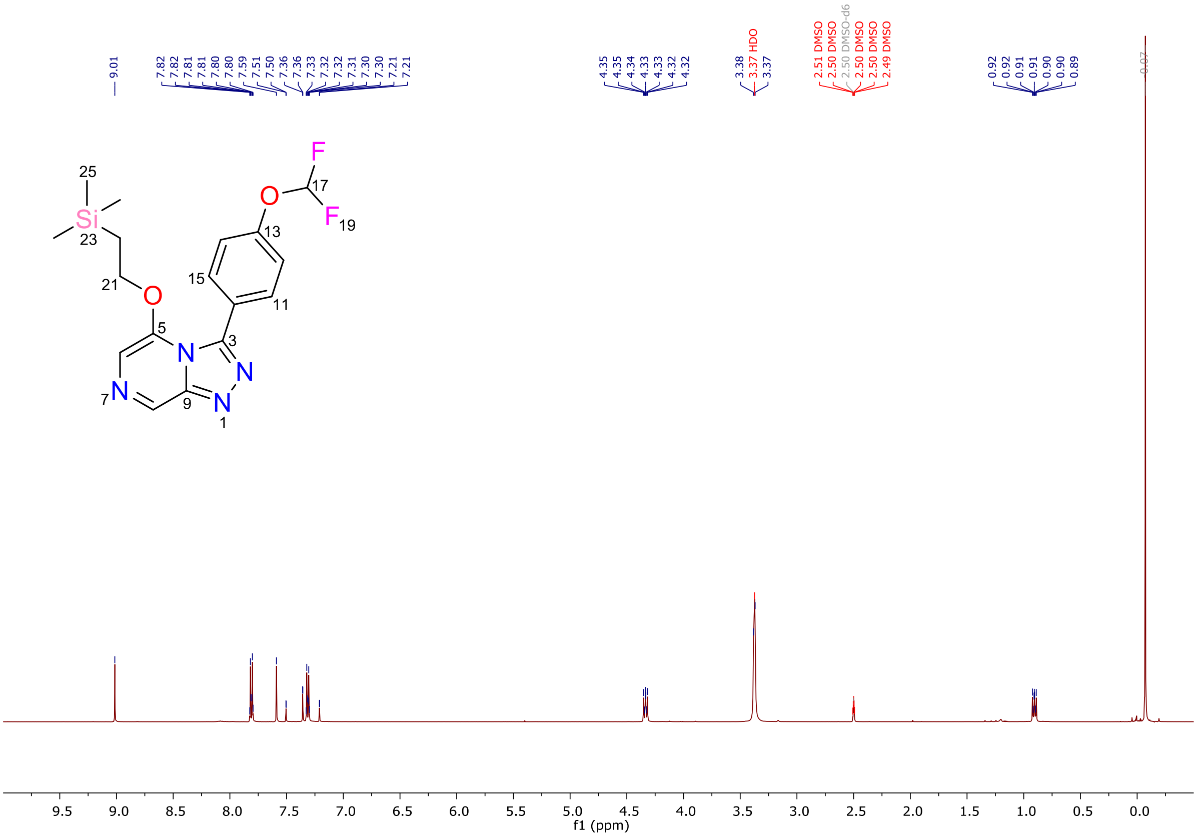

S26: ^{13}C NMR spectrum of compound **5** in DMSO-d_6

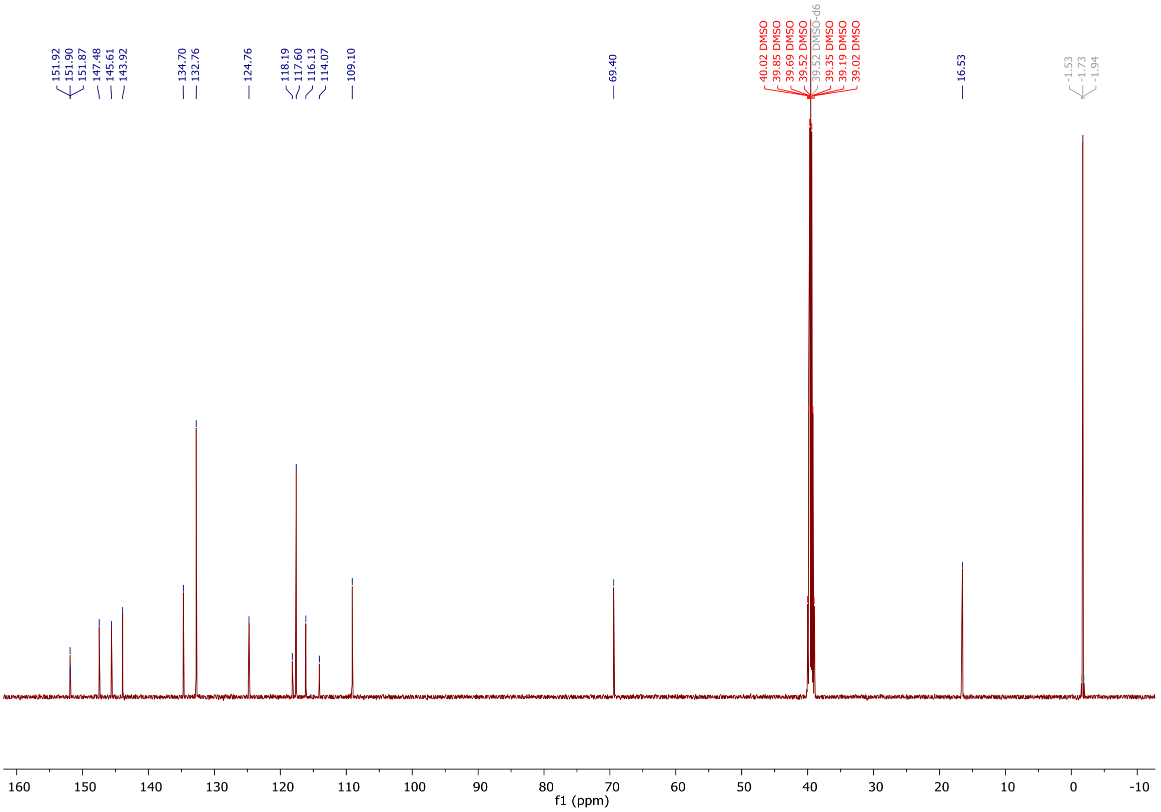

S27: COSY spectrum of compound **5** in DMSO-d_6

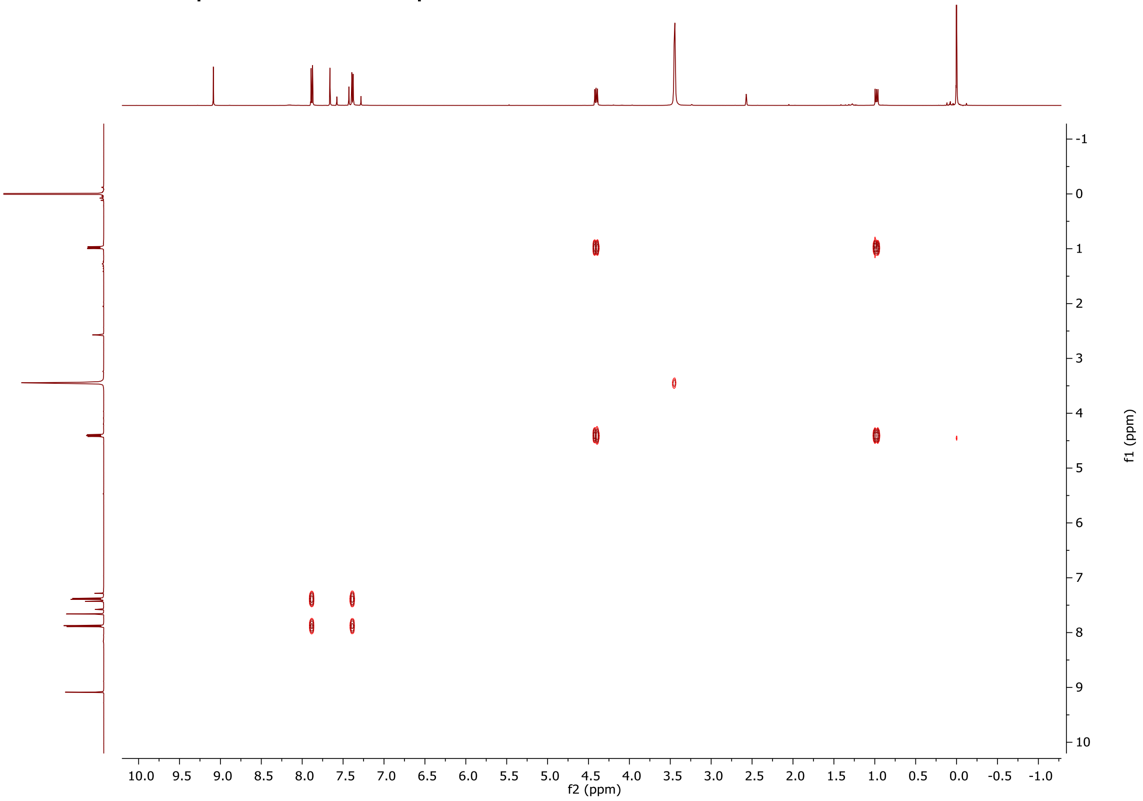

S28: HSQC spectrum of compound **5** in DMSO-d_6

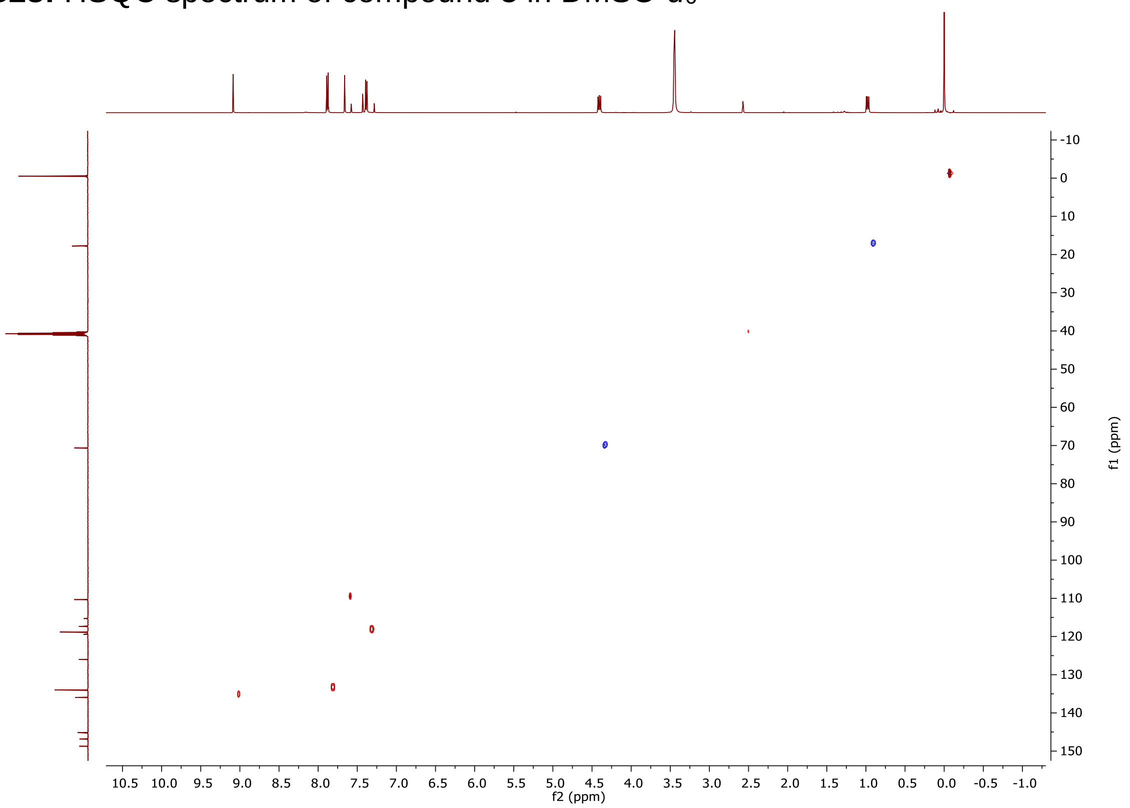

S29: HMBC spectrum of compound **5** in DMSO-d_6

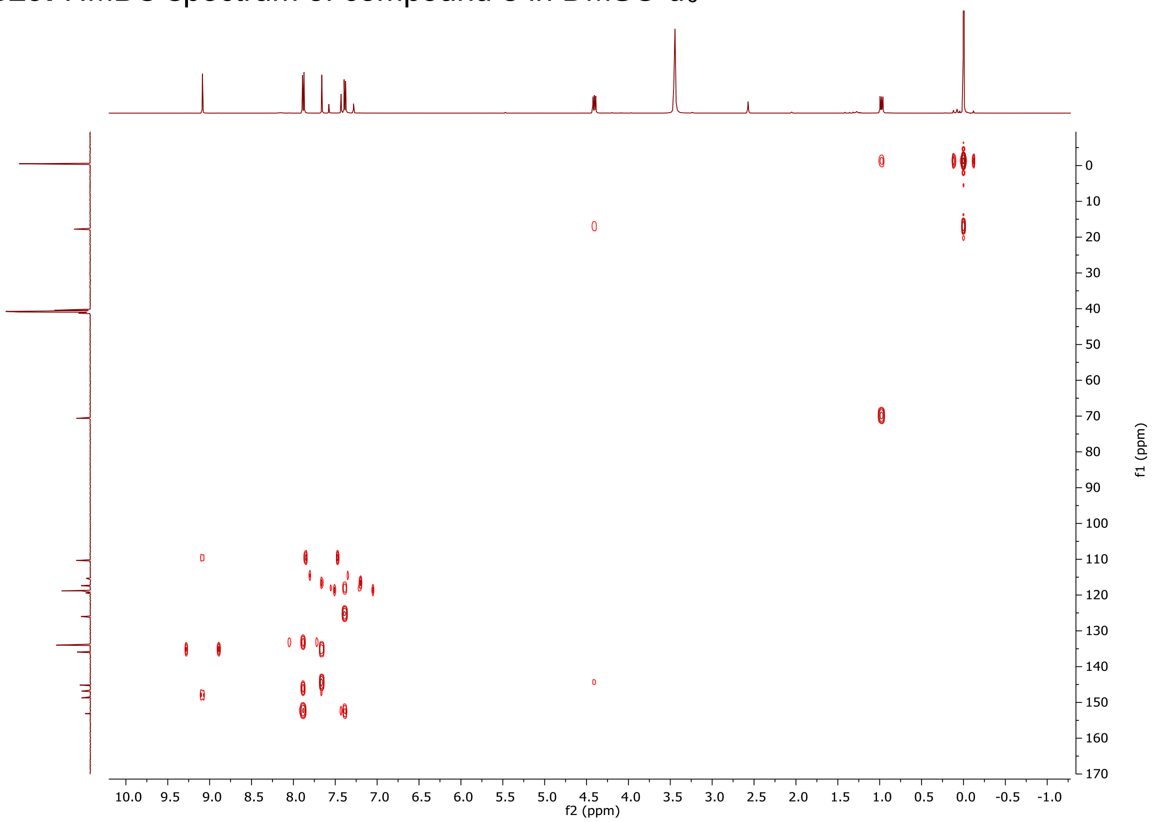

S30: ROESY spectrum of compound **5** in DMSO-d_6

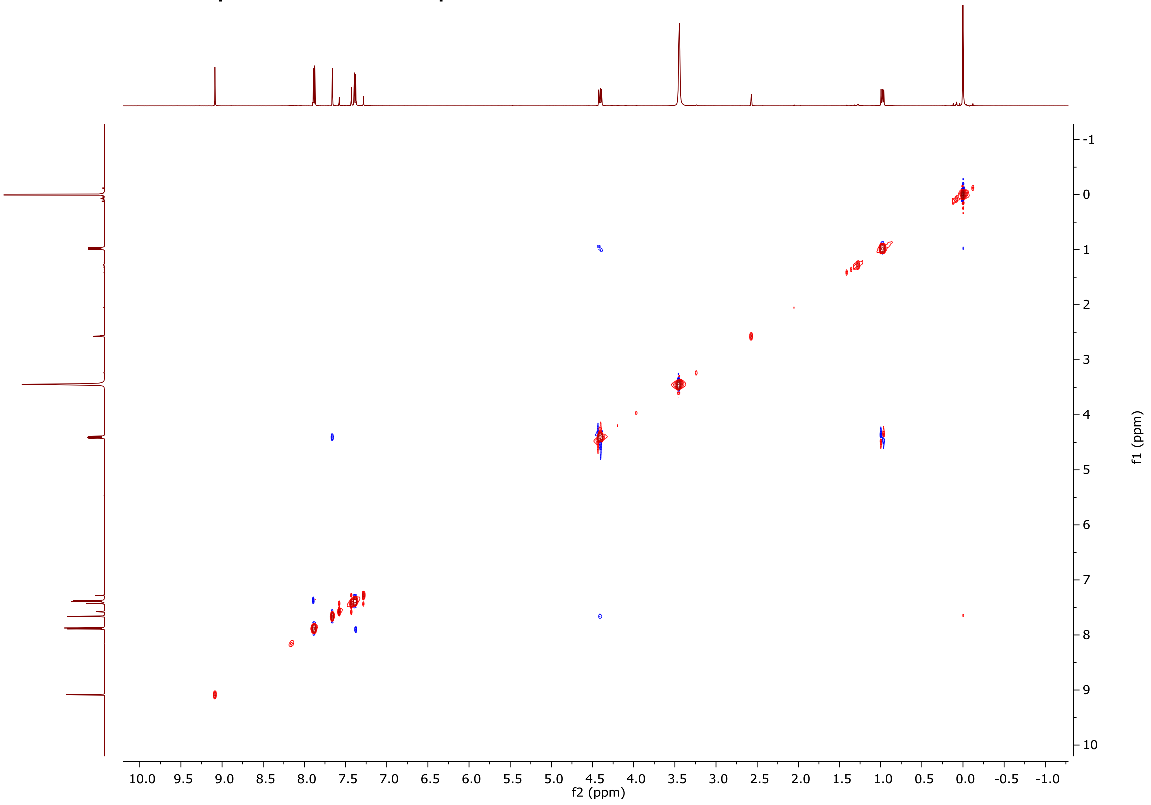

S31: HRMS of compound **5**

Griffith UNIVERSITY

High Resolution Mass Spectrum

Analysis Info

Analysis Name	D:\Data\KahYean\20200508\RAD860000001.d	Acquisition Date	5/12/2020 12:13:02 PM
Method	DirectInfusion_2018_pos.m	Instrument	maXis II ETD 1823391.22321
Sample Name	RAD860		
Comment			

+MS, 0.1-0.1min #5-6

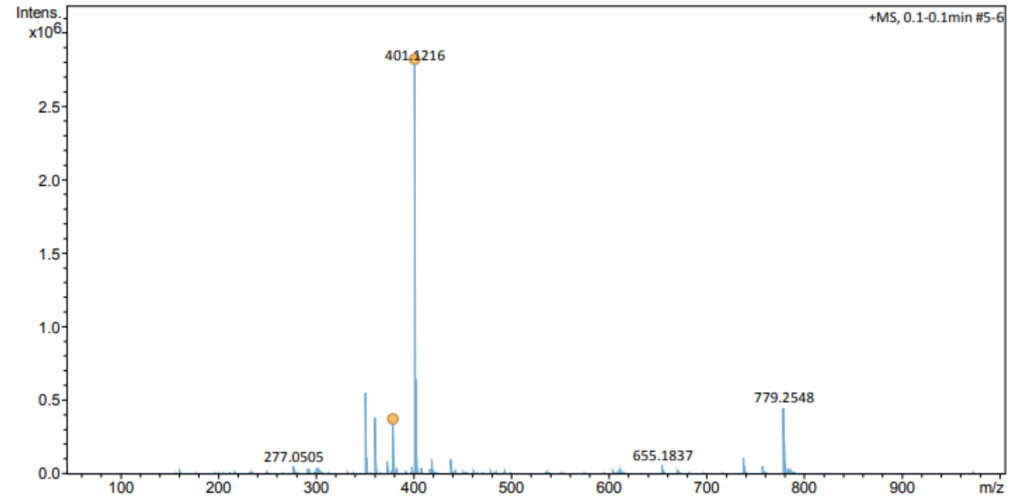

Meas. m/z	#	Ion Formula	m/z	err [ppm]	mSigma	# mSigma	Score	rdb	e^- Conf	N-Rule
379.1396	1	C17H21F2N4O2Si	379.1396	0.1	20.5	1	100.00	9.5	even	ok
401.1216	1	C17H20F2N4NaO2Si	401.1216	0.0	15.5	1	100.00	9.5	even	ok

S32: NMR data of compound **6** in DMSO-d_6[a]

Pos.	δ_H, mult. (*J* in Hz), int.	δ_C, mult. (*J* in Hz)	COSY	HMBC
3		147.2, s		
5	8.17, d (4.9), 1H	111.9, s	6	6, 9
6	7.47, d (4.9), 1H	127.6, s	5	5, 8
8		153.3, s		
9		139.9, s		
10		122.7, s		
11	7.98, m, 1H	130.2, s	12	3, 13,15
12	7.43, m, 1H	119.2, s	11	10,14
13		152.4, t (3.3)		
14	7.43, m, 1H	119.2, s	15	10, 12
15	7.98, m, 1H	130.2, s	14	3, 11, 13
17	7.41, t (73.7), 1H	116.1, t (258.4)		13
21	4.65, m, 2H	65.2, s	22	22, 8
22	1.24, m, 2H	16.8, s	21	21, 24, 25, 26
24	0.11, s, 3H	-1.3, s		22, 25, 26
25	0.11, s, 3H	-1.3, s		22, 24, 26
26	0.11, s, 3H	-1.3, s		22, 24, 25

[a] Recorded at 500 MHz (^{1}H NMR) and 125 MHz (^{13}C NMR) at 25 °C.

S33: ^{1}H NMR spectrum of compound **6** in DMSO-d_6

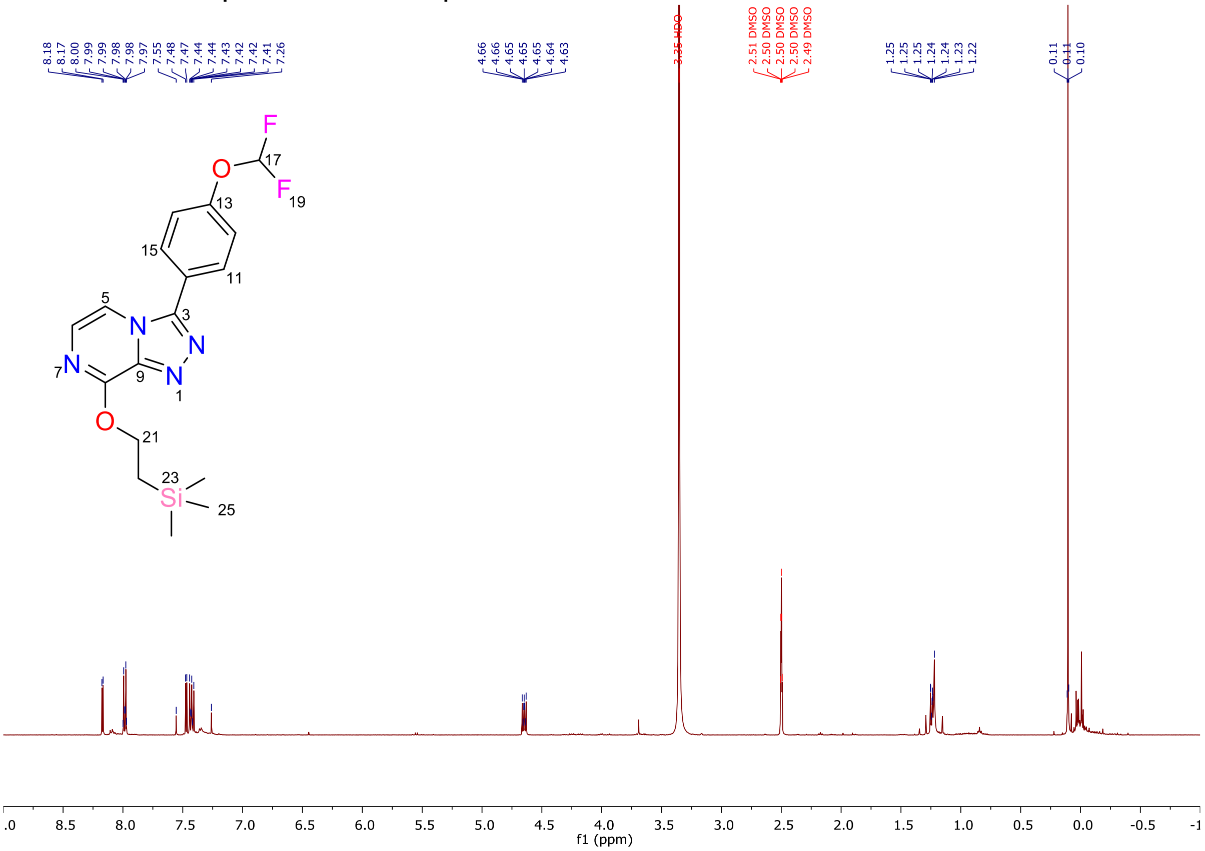

S34: ^{13}C NMR spectrum of compound **6** in DMSO-d_6

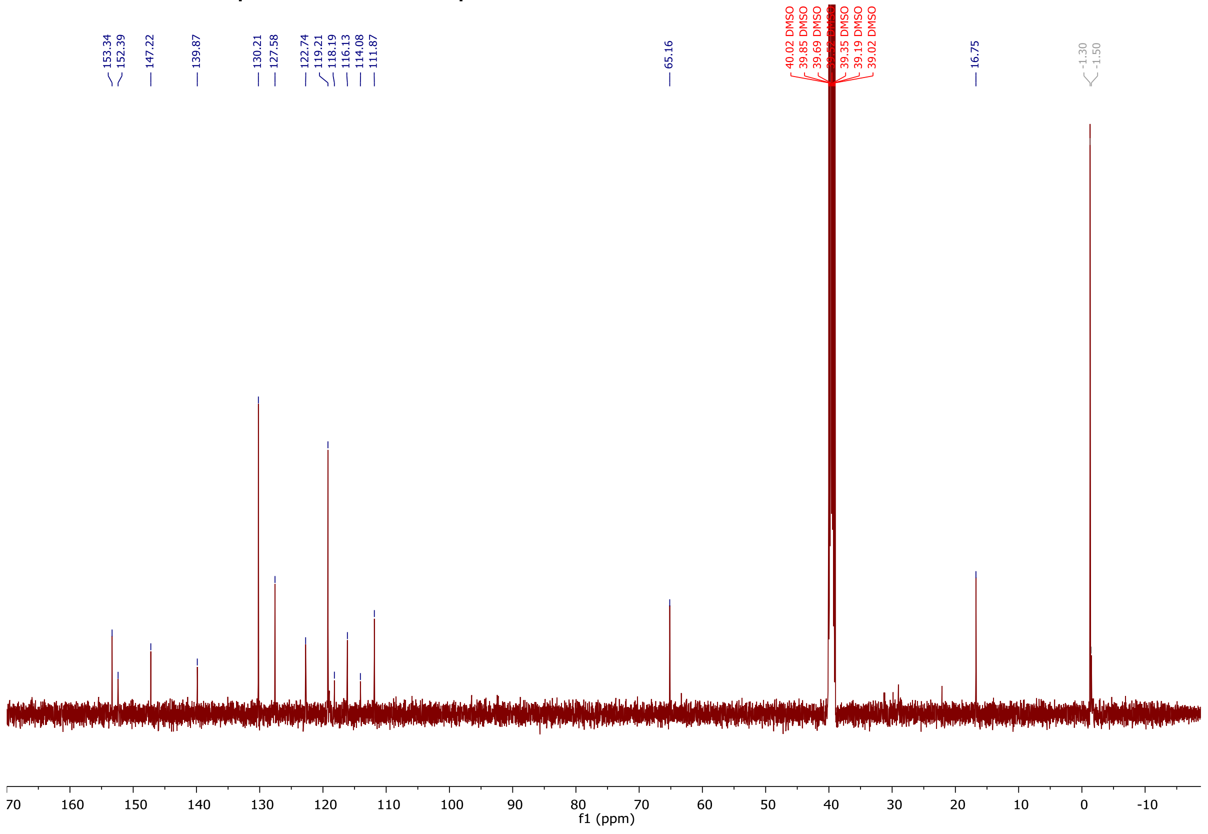

S35: COSY spectrum of compound **6** in DMSO-d_6

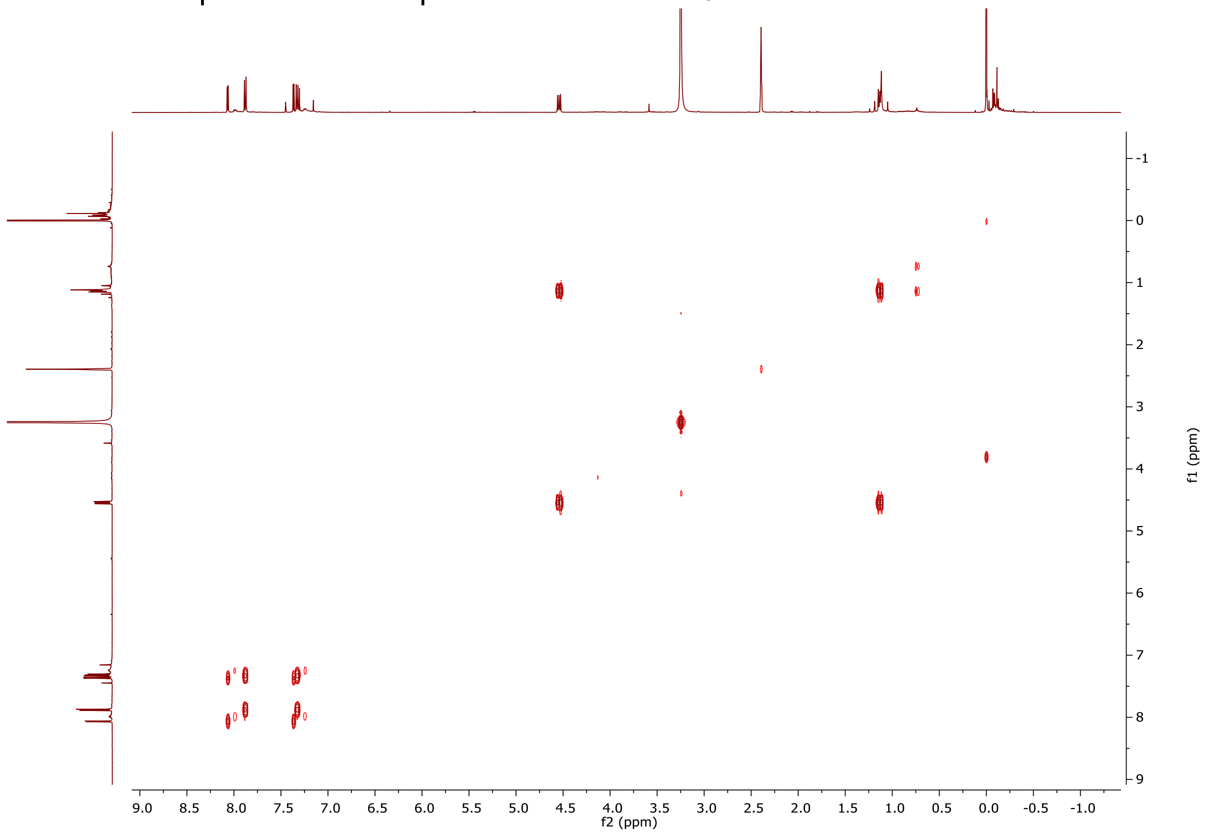

S36: HSQC spectrum of compound **6** in DMSO-d_6

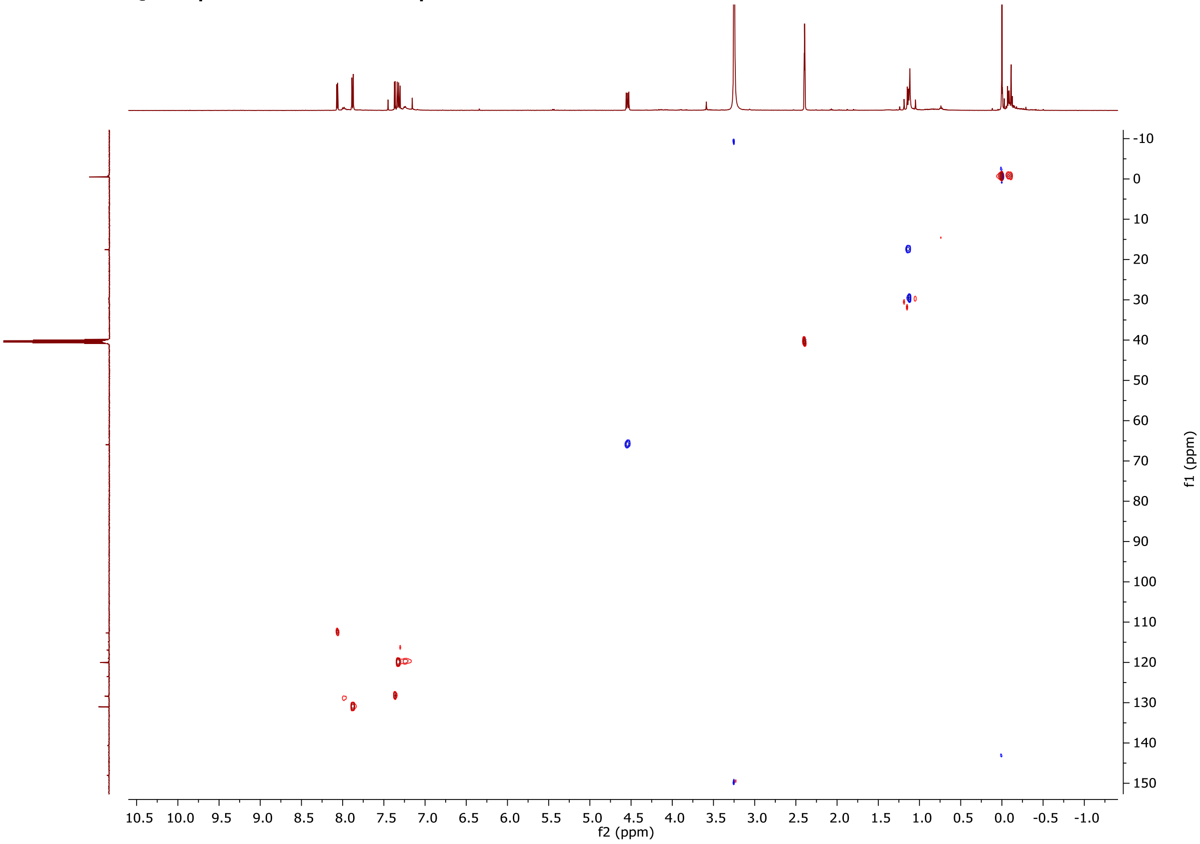

S37: HMBC spectrum of compound **6** in DMSO-d_6

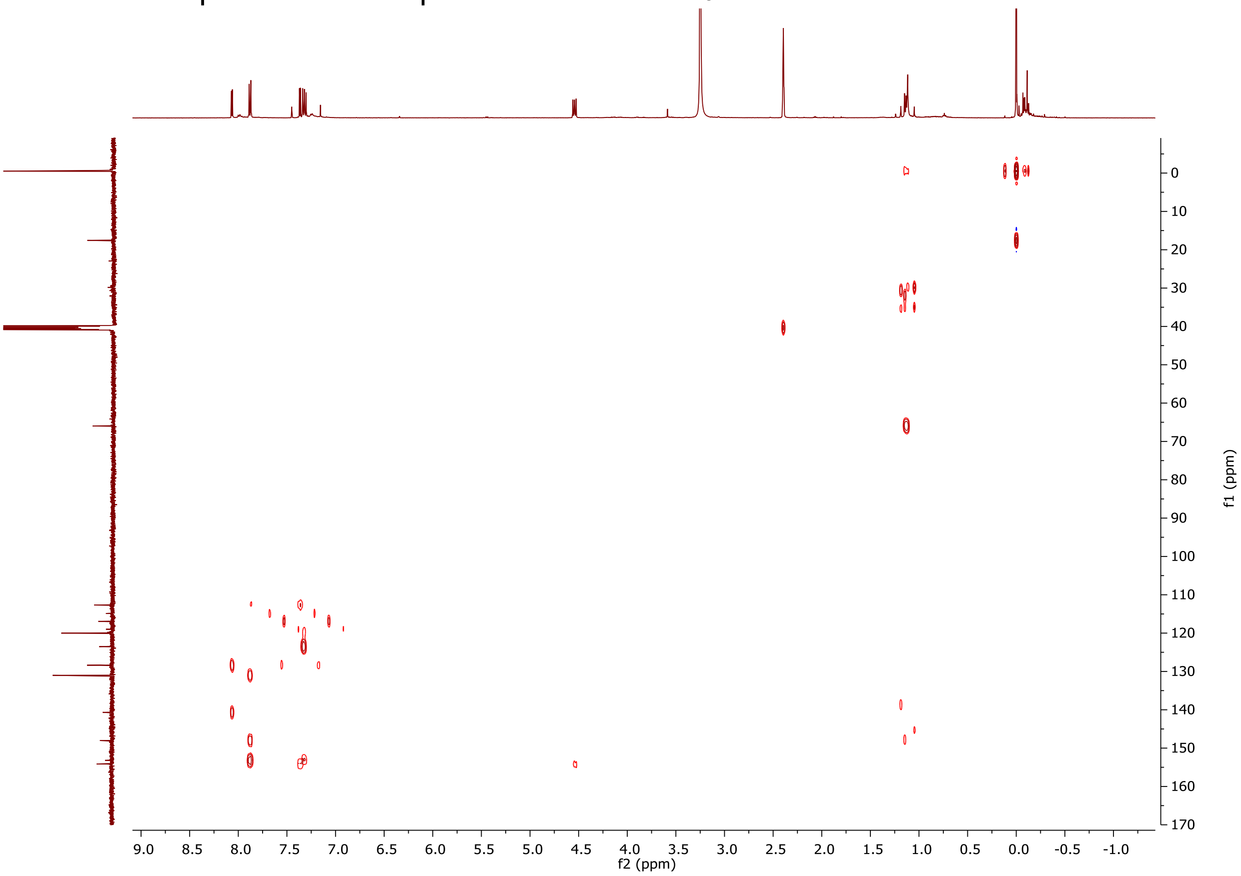

S38: HRMS of compound **6**

High Resolution Mass Spectrum

Analysis Info

Analysis Name	D:\Data\KahYean\20200508\RAD861000002.d		
Method	DirectInfusion_2018_pos.m	Acquisition Date	5/12/2020 12:26:20 PM
Sample Name	RAD861	Instrument	maXis II ETD 1823391.22321
Comment			

+MS, 0.7-0.7min #38-39

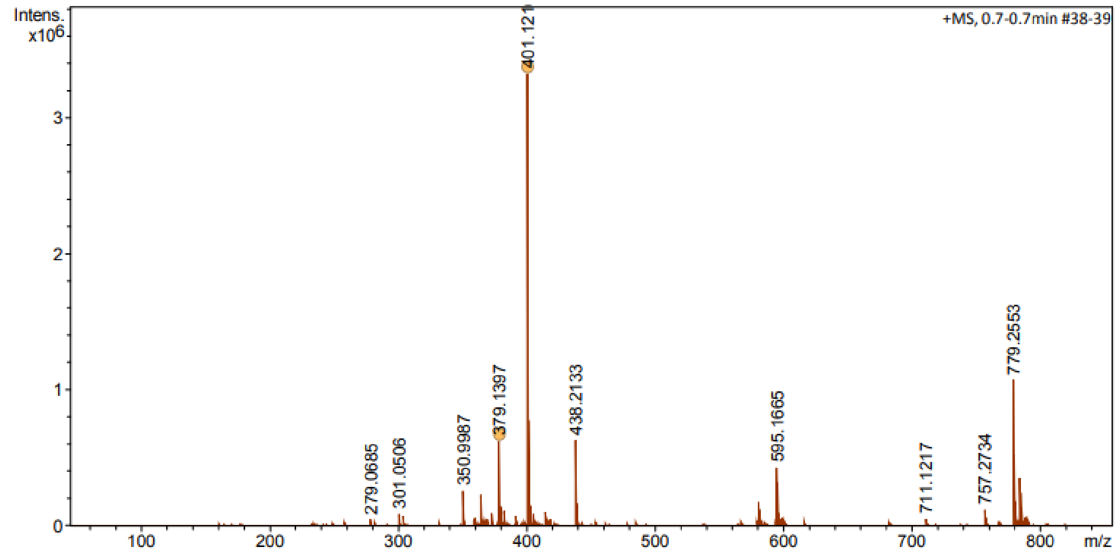

Meas. m/z	#	Ion Formula	m/z	err [ppm]	mSigma	# mSigma	Score	rdb	e⁻ Conf	N-Rule
379.1397	1	C17H21F2N4O2Si	379.1396	-0.1	17.1	1	100.00	9.5	even	ok
401.1216	1	C17H20F2N4NaO2Si	401.1216	-0.1	14.3	1	100.00	9.5	even	ok

S39: NMR data of compound **8** in DMSO-d_6[a]

Position	δ_H, mult. (*J* in Hz)	δ_C	COSY	HMBC
3		147.2		
5		119.8		
6	7.92, s, 1H	128.5		5, 8
8		151.5		
9		146.7		
10		126.3		
11	7.74, m, 1H	133.1	12	3, 13,15
12	7.63, m, 1H	127.9	11	10,14
13		135.4		
14	7.63, m, 1H	127.9	15	10, 12
15	7.74, m, 1H	133.1	14	3, 11, 13
18	2.86, s, 3H	20.2		5, 6, 8, 9

[a] Recorded at 500 MHz (^{1}H NMR) and 125 MHz (^{13}C NMR) at 25 °C.

S40: ^{1}H NMR spectrum of compound **8** in DMSO-d_6

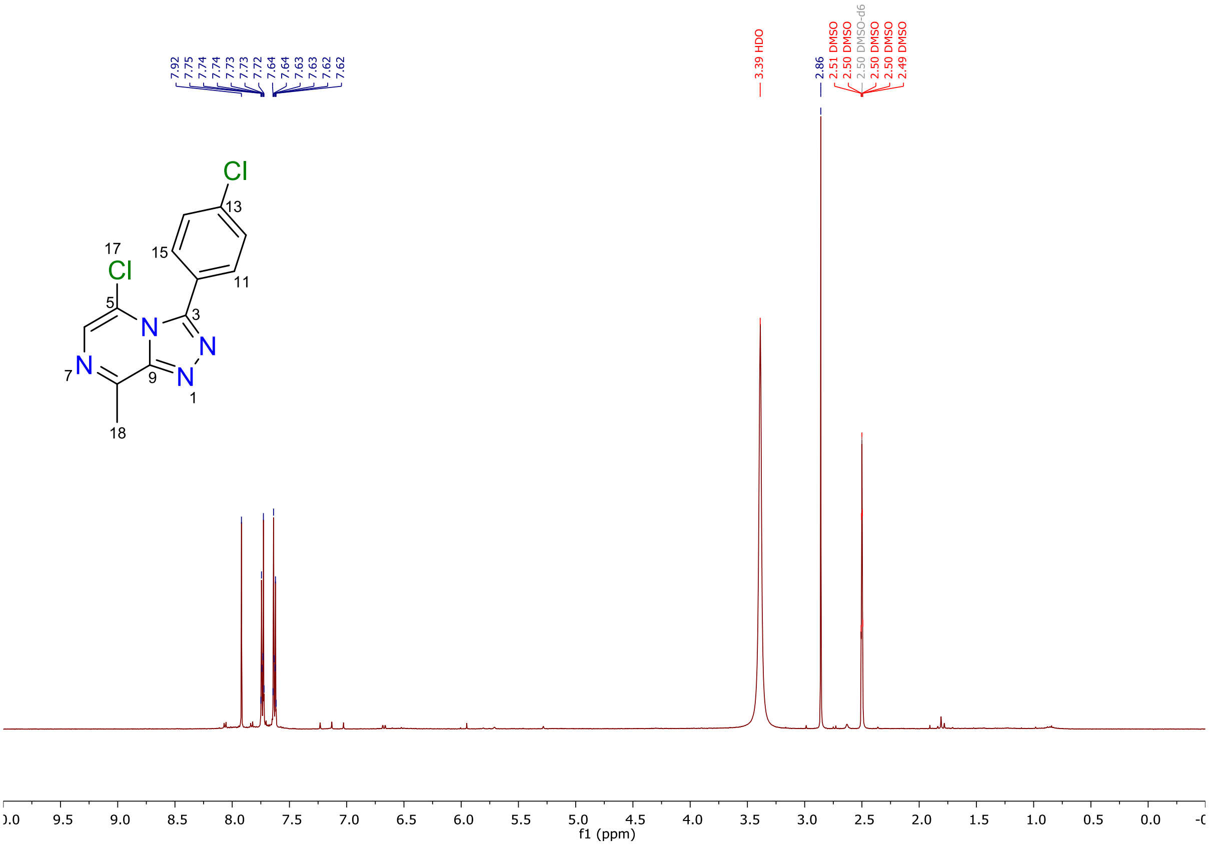

S41: ^{13}C NMR spectrum of compound **8** in DMSO-d_6

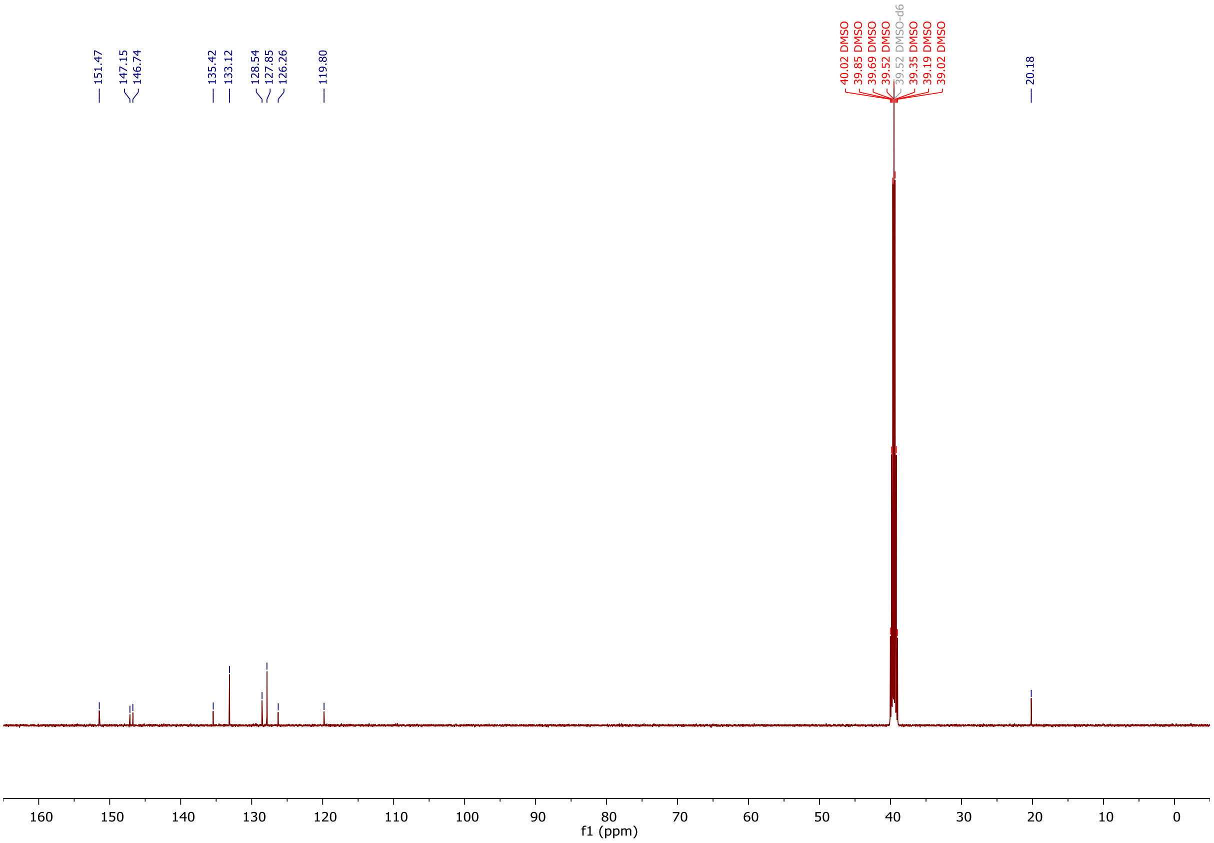

S42: COSY spectrum of compound **8** in DMSO-d_6

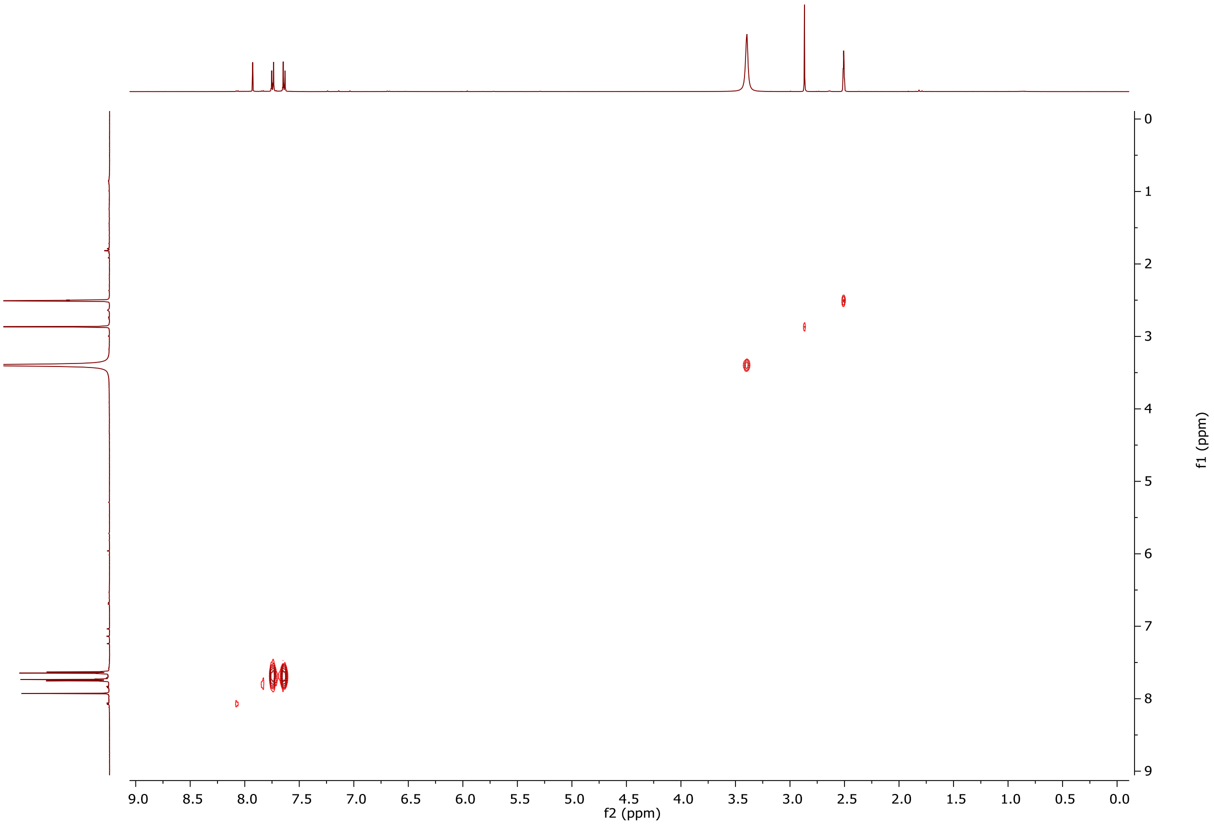

S43: HSQC spectrum of compound **8** in DMSO-d_6

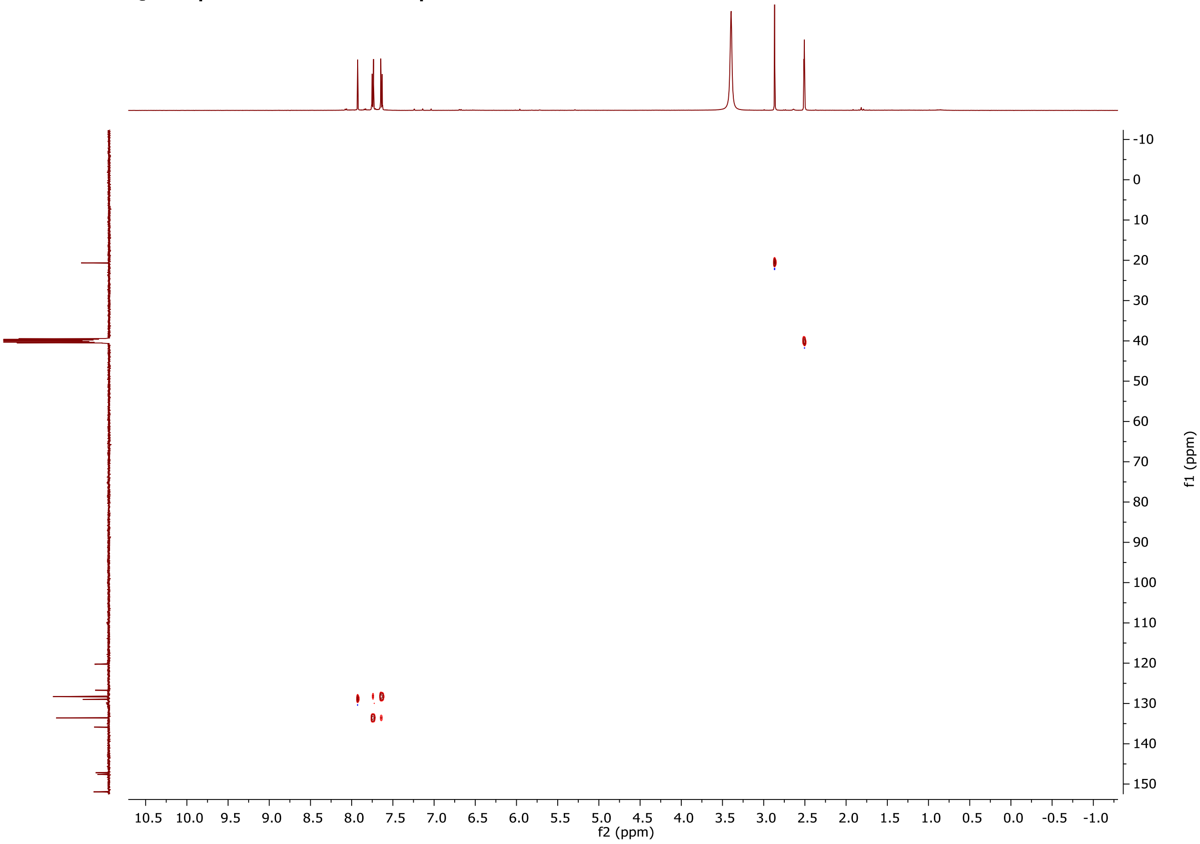

S44: HMBC spectrum of compound **8** in DMSO-d_6

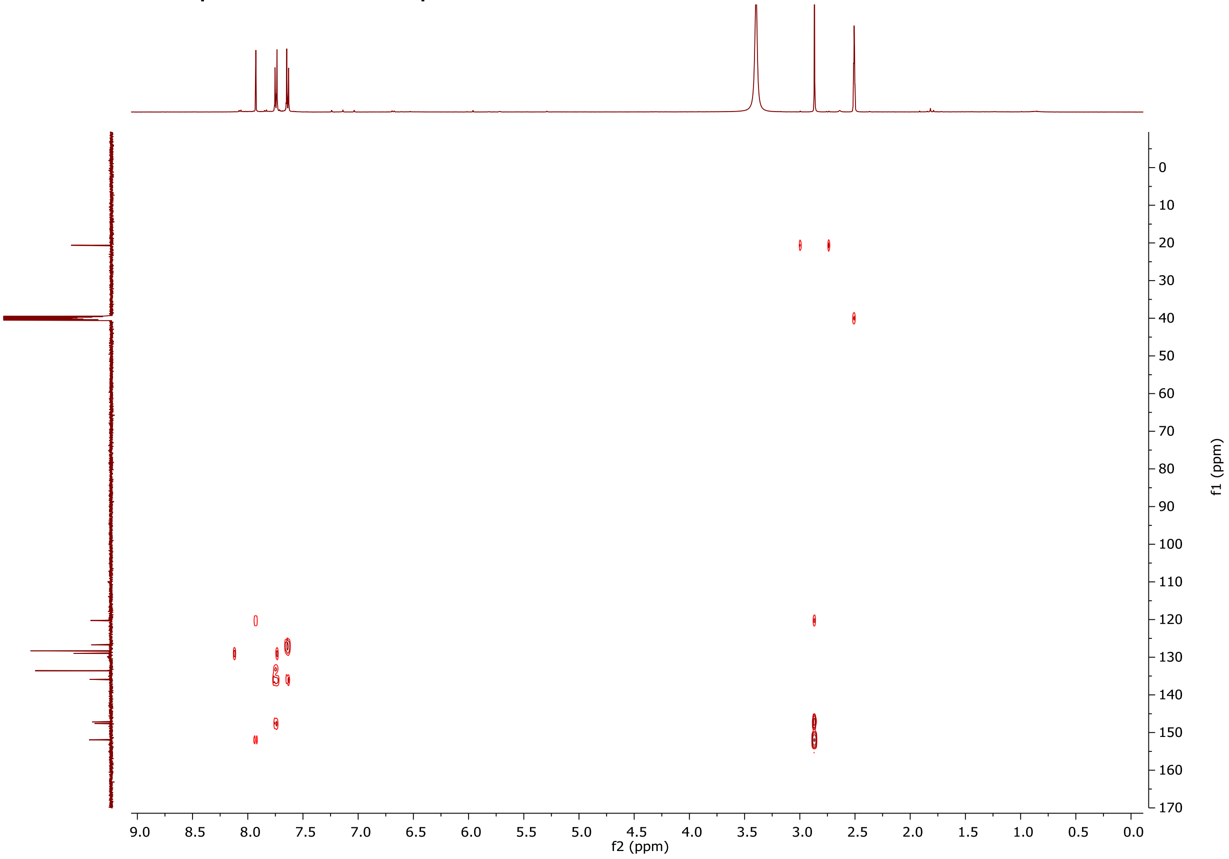

S45: HRMS of compound **8**

Griffith UNIVERSITY

High Resolution Mass Spectrum

Analysis Info

Analysis Name	D:\Data\KahYean\20200508\RAD857000003.d		
Method	DirectInfusion_2018_pos.m	Acquisition Date	5/12/2020 11:00:21 AM
Sample Name	RAD857	Instrument	maXis II ETD 1823391.22321
Comment			

+MS, 0.1min #6

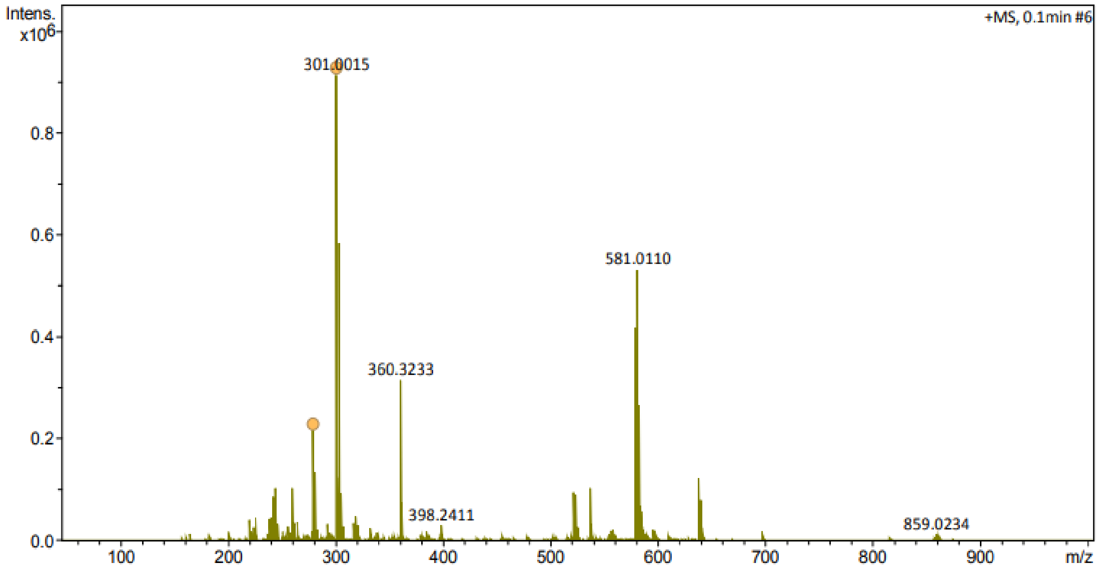

Meas. m/z	#	Ion Formula	m/z	err [ppm]	mSigma	# mSigma	Score	rdb	e⁻ Conf	N-Rule
279.0197	1	C12H9Cl2N4	279.0199	0.8	9.6	1	100.00	9.5	even	ok
301.0015	1	C12H8Cl2N4Na	301.0018	0.9	6.9	1	100.00	9.5	even	ok

S46: NMR data of compound **10** in DMSO-d_6[a]

Position	δ_H, mult. (J in Hz)	δ_C	COSY	HMBC
3		146.76#		
5		119.8		
6	7.96, s, 1H	128.7		5, 8
8		151.5		
9		146.83#		
10		132.0		
11	7.93, m, 1H	132.2	12	3, 13,15
12	8.04, m, 1H	131.6	11	10, 14, 16
13		113.0		
14	8.04, m, 1H	131.6	15	10, 12, 16
15	7.93, m, 1H	132.2	14	3, 11, 13
16		118.4		
19	2.87, s, 3H	20.2		5, 6, 8, 9

[a] Recorded at 500 MHz (^{1}H NMR) and 125 MHz (^{13}C NMR) at 25 °C. # Interchangeable signals.

S47: ^{1}H NMR spectrum of compound **10** in DMSO-d_6

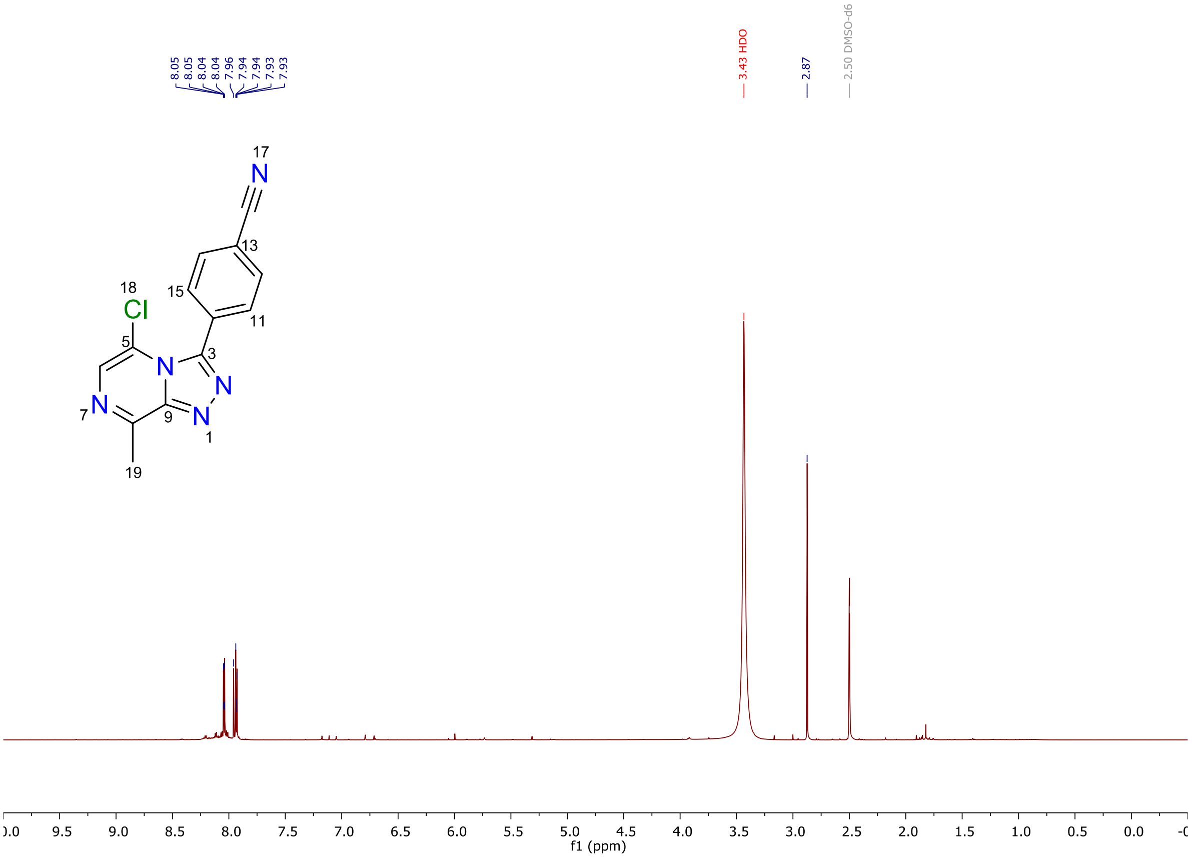

S48: ^{13}C NMR spectrum of compound **10** in DMSO-d_6

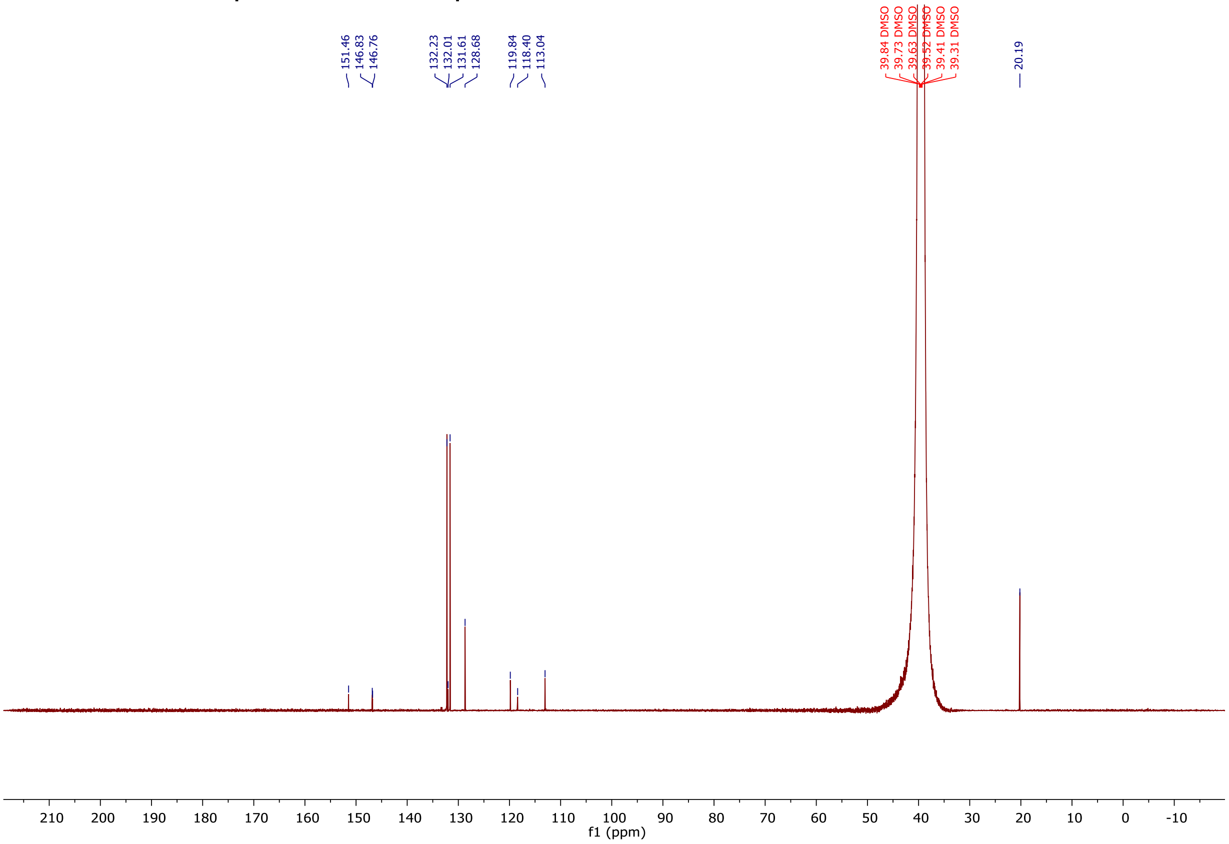

S49: COSY spectrum of compound **10** in DMSO-d_6

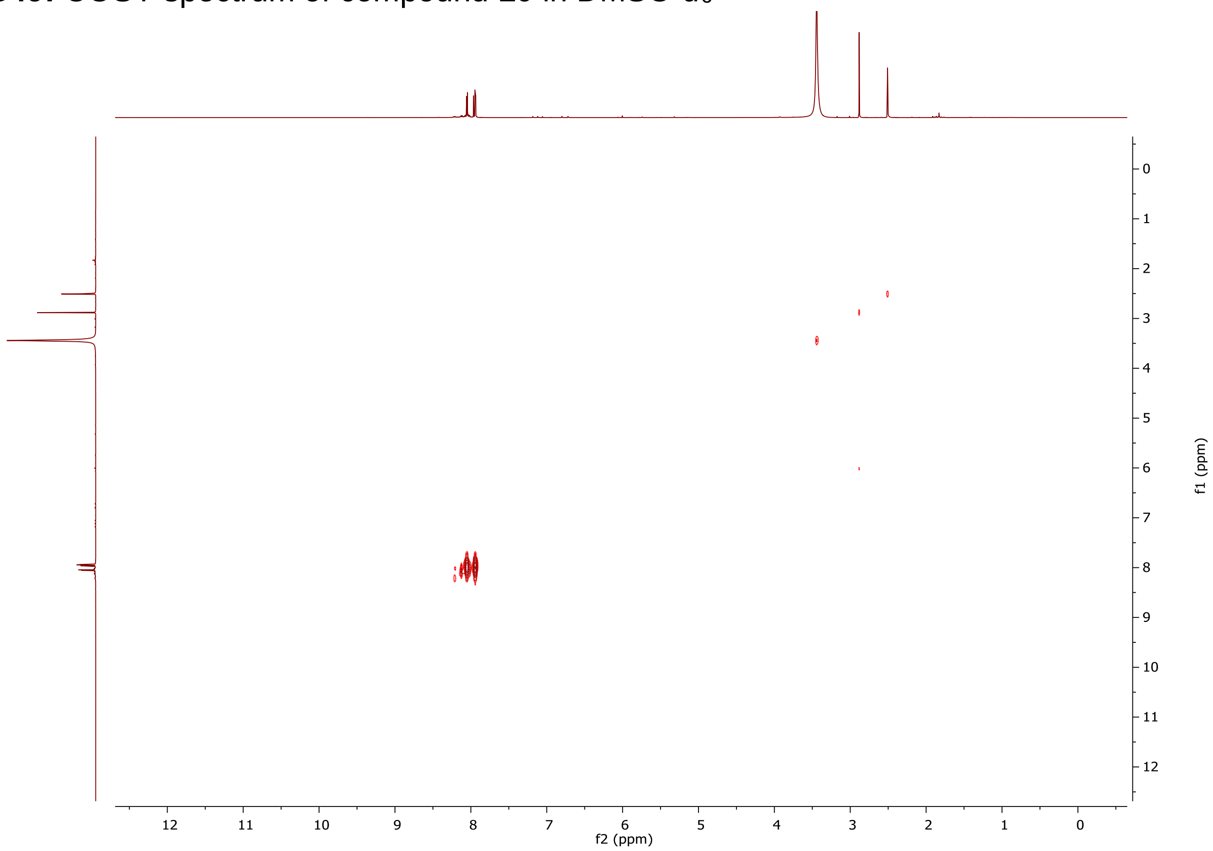

S50: HSQC spectrum of compound **10** in DMSO-d_6

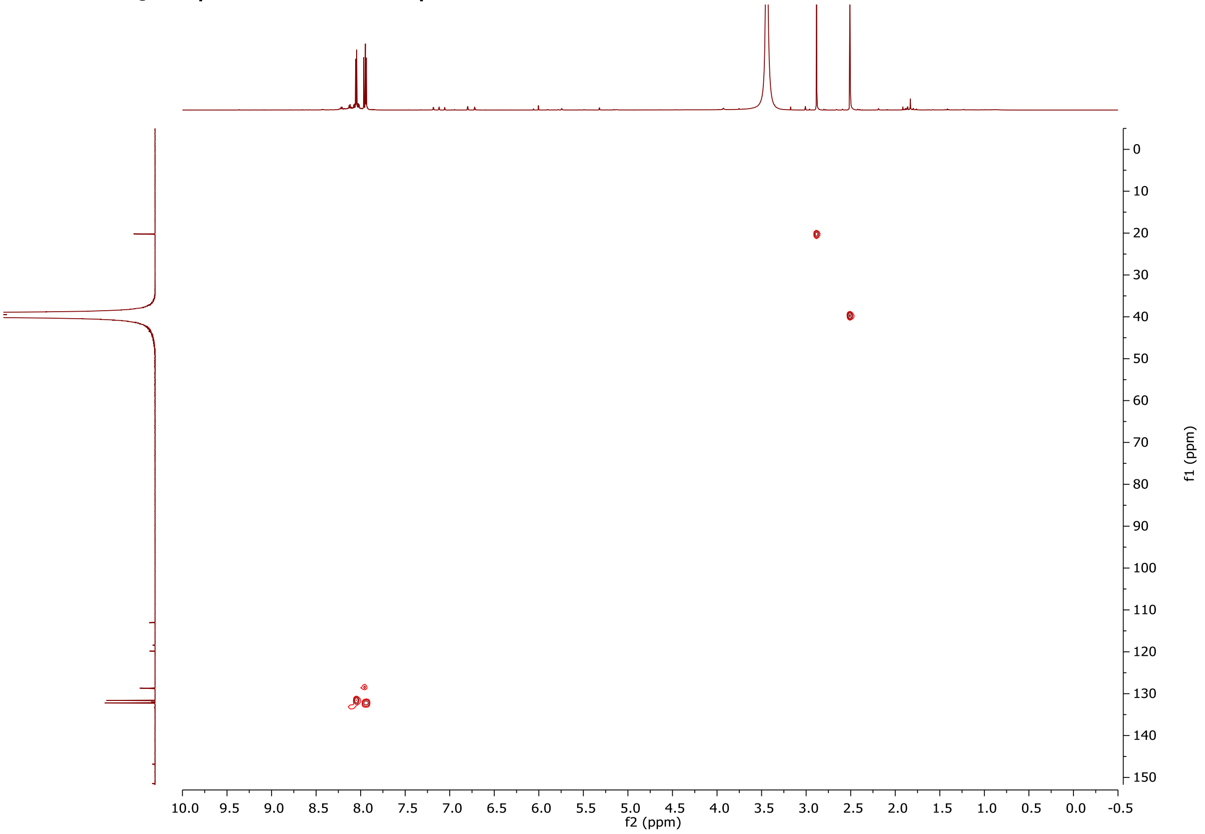

S51: HMBC spectrum of compound **10** in DMSO-d_6

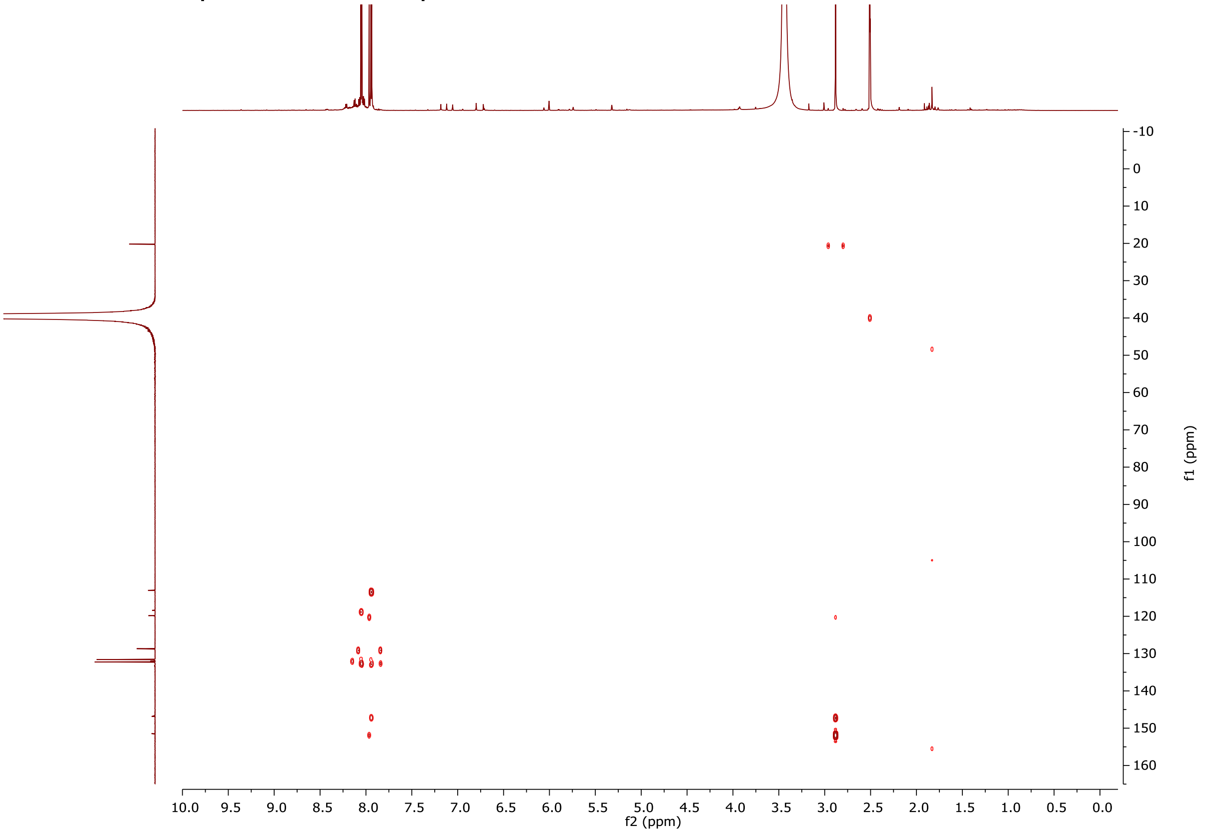

S52: HRMS of compound **10**

High Resolution Mass Spectrum

Analysis Info

Analysis Name D:\Data\KahYean\20200508\RAD858000001.d
Method DirectInfusion_2018_pos.m
Sample Name RAD858
Comment

Acquisition Date 5/12/2020 11:09:33 AM
Instrument maXis II ETD 1823391.22321

+MS, 0.1-0.1min #5-6

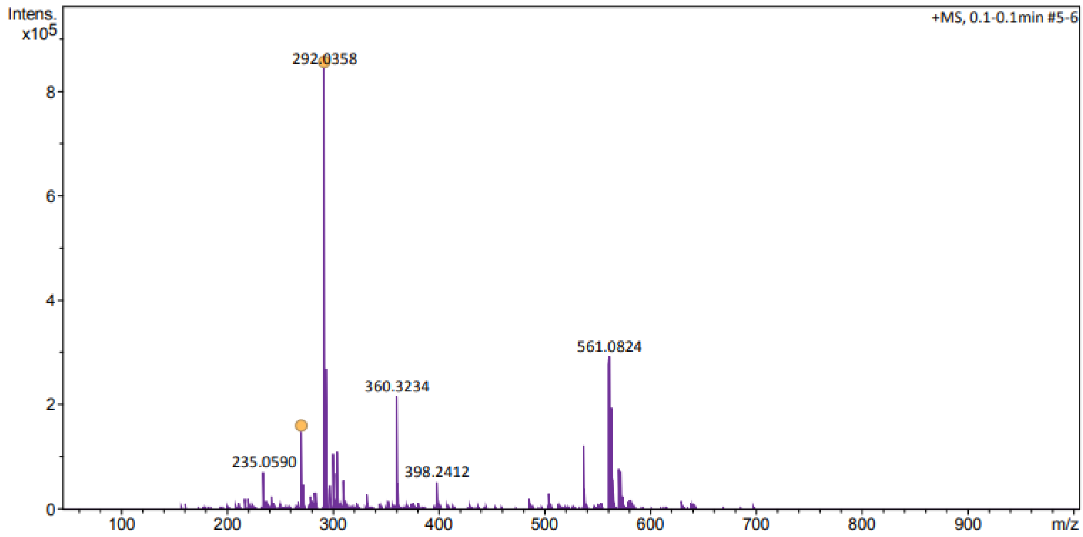

Meas. m/z	#	Ion Formula	m/z	err [ppm]	mSigma	# mSigma	Score	rdb	e⁻ Conf	N-Rule
270.0540	1	C13H9ClN5	270.0541	0.4	10.8	1	100.00	11.5	even	ok
292.0358	1	C13H8ClN5Na	292.0360	0.7	8.2	1	100.00	11.5	even	ok

S53: NMR data of compound **11** in DMSO-d_6[a]

Pos	δ_H, mult. (*J* in Hz), int.	δ_C, mult. (*J* in Hz)	COSY	HMBC
3		147.4, s		
5		119.8, s		
6	7.91, s, 1H	128.5, s		5, 8
8		151.5, s		
9		146.7, s		
10		124.0, s		
11	7.77, m, 1H	133.3, s	12	3, 13,15
12	7.34, m, 1H	117.5, s	11	10, 14
13		152.5, t (3.3)		
14	7.34, m, 1H	117.5, s	15	10, 12
15	7.77, m, 1H	133.3, s	14	3, 11, 13
17	7.40, t (73.7), 1H	116.2, t (258.5)		13
21	2.86, s, 3H	20.2, s		5, 6, 8, 9

[a] Recorded at 500 MHz (^{1}H NMR) and 125 MHz (^{13}C NMR) at 25 °C.

S54: ^{1}H NMR spectrum of compound **11** in DMSO-d_6

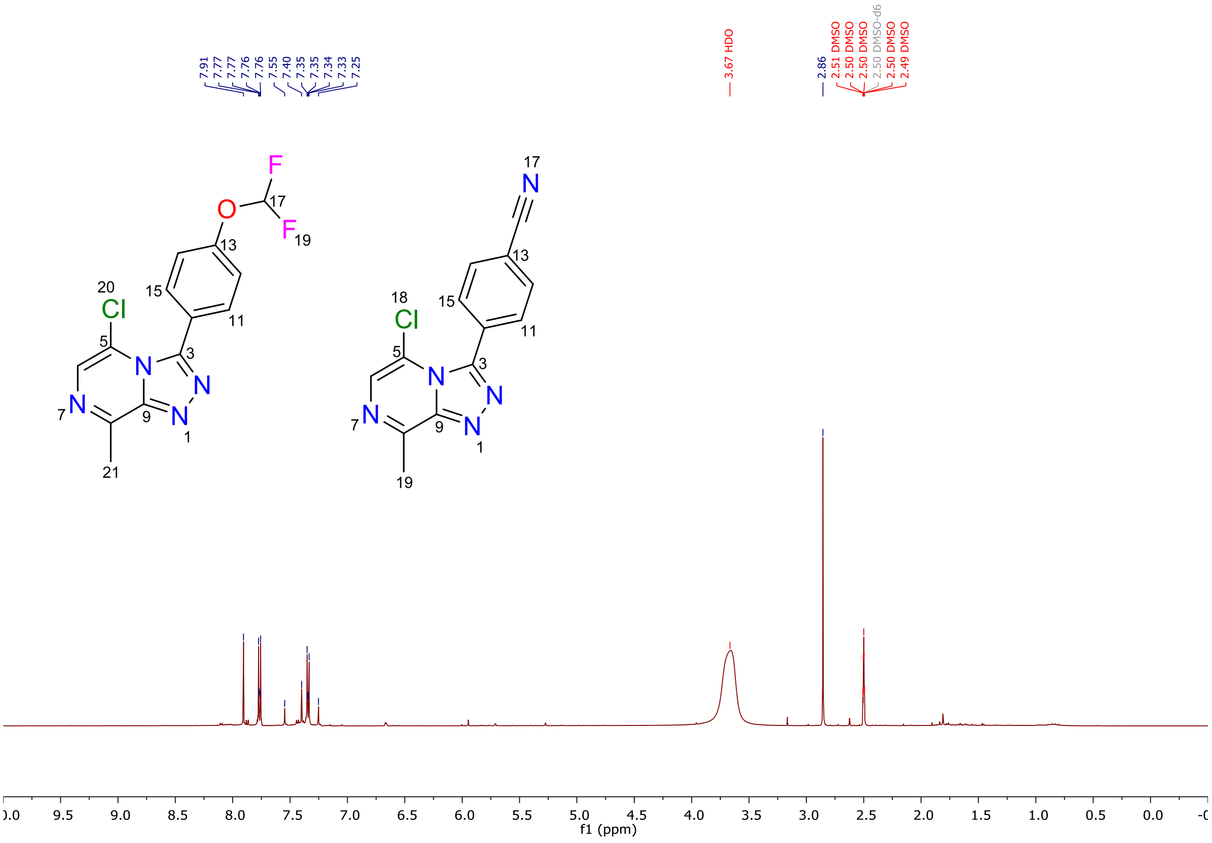

S55: ^{13}C NMR spectrum of compound **11** in DMSO-d_6

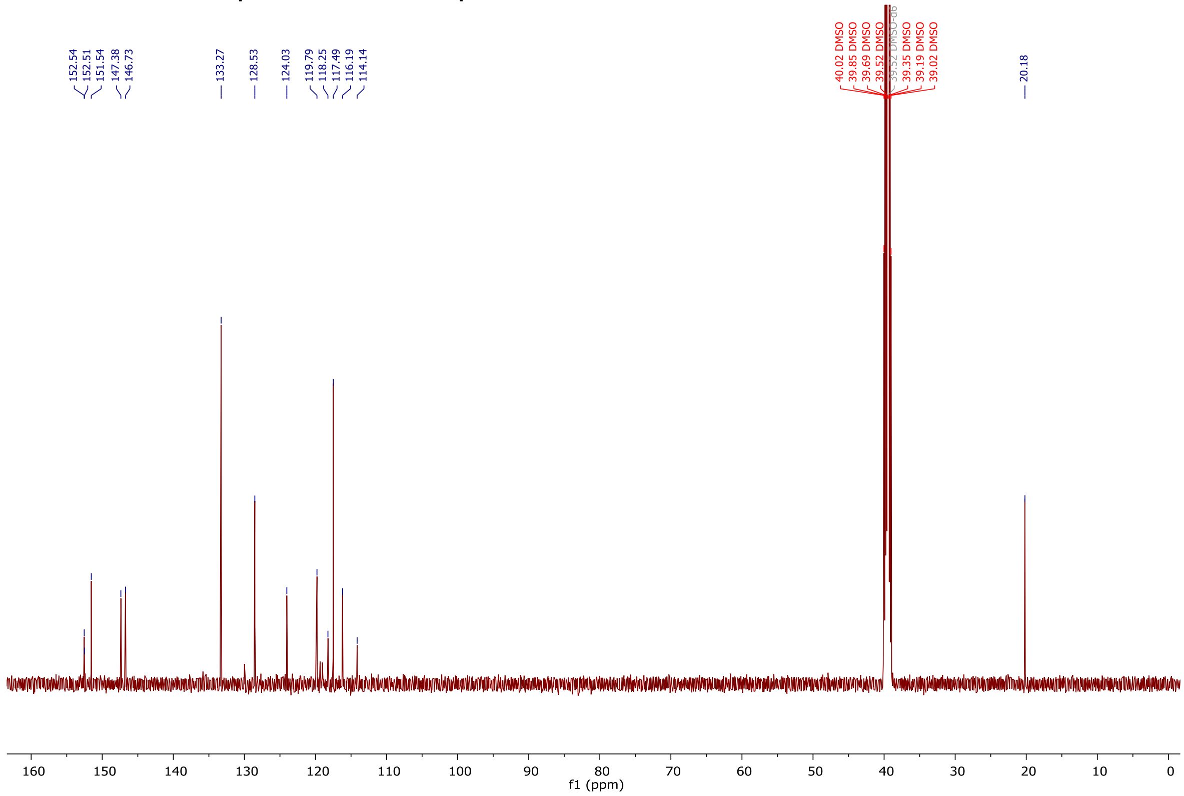

S56: COSY spectrum of compound **11** in DMSO-d_6

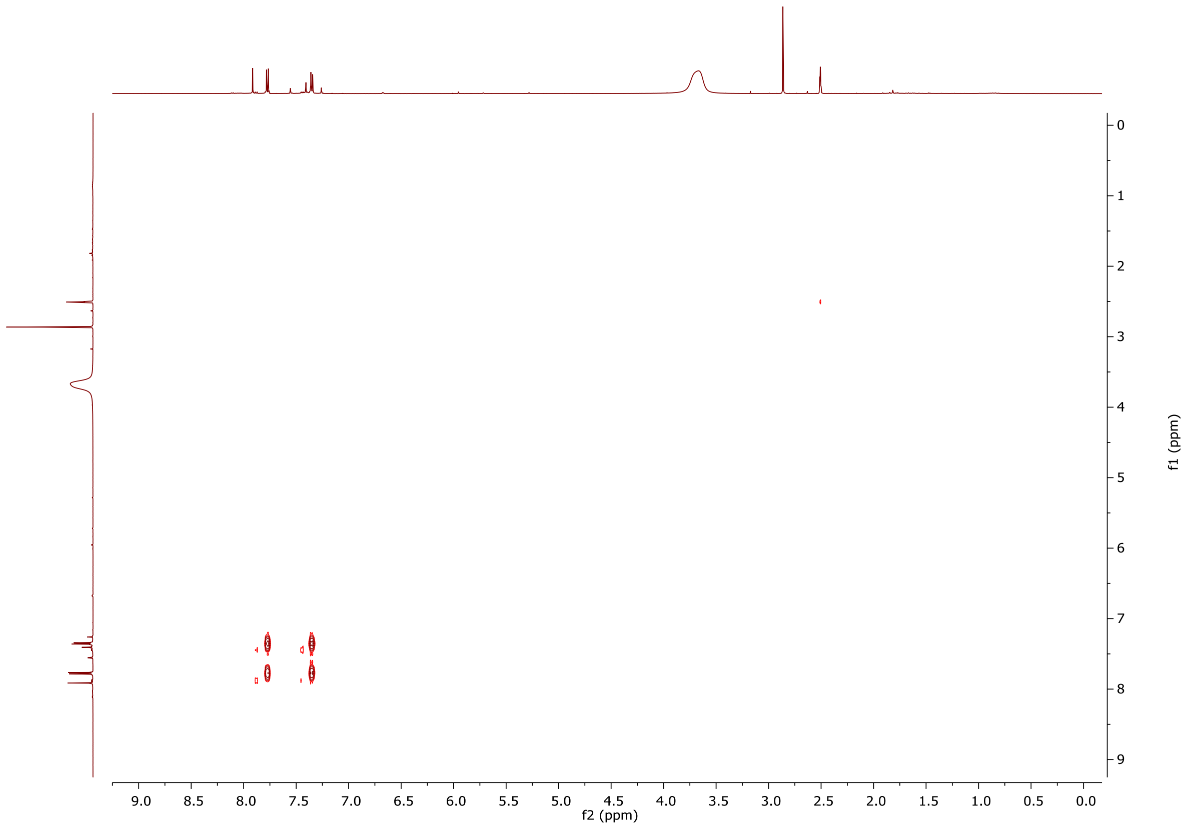

S57: HSQC spectrum of compound **11** in DMSO-d_6

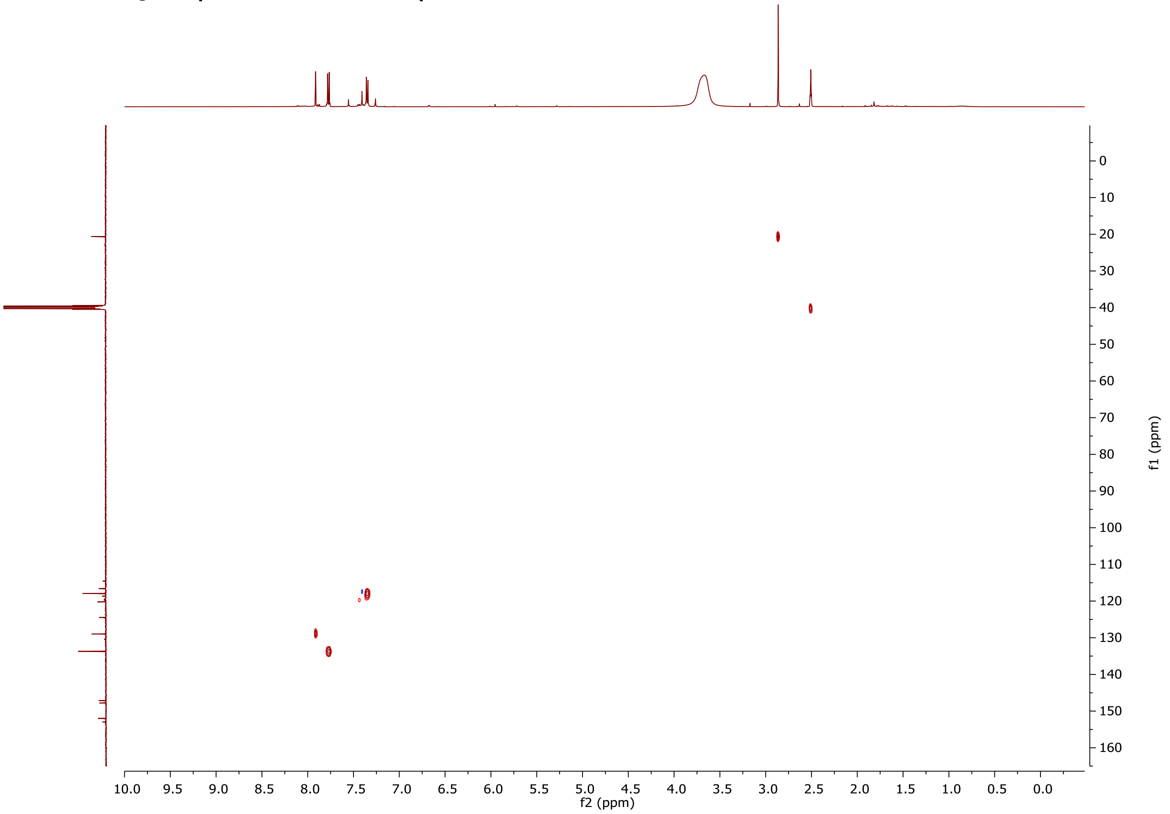

S58: HMBC spectrum of compound **11** in DMSO-d_6

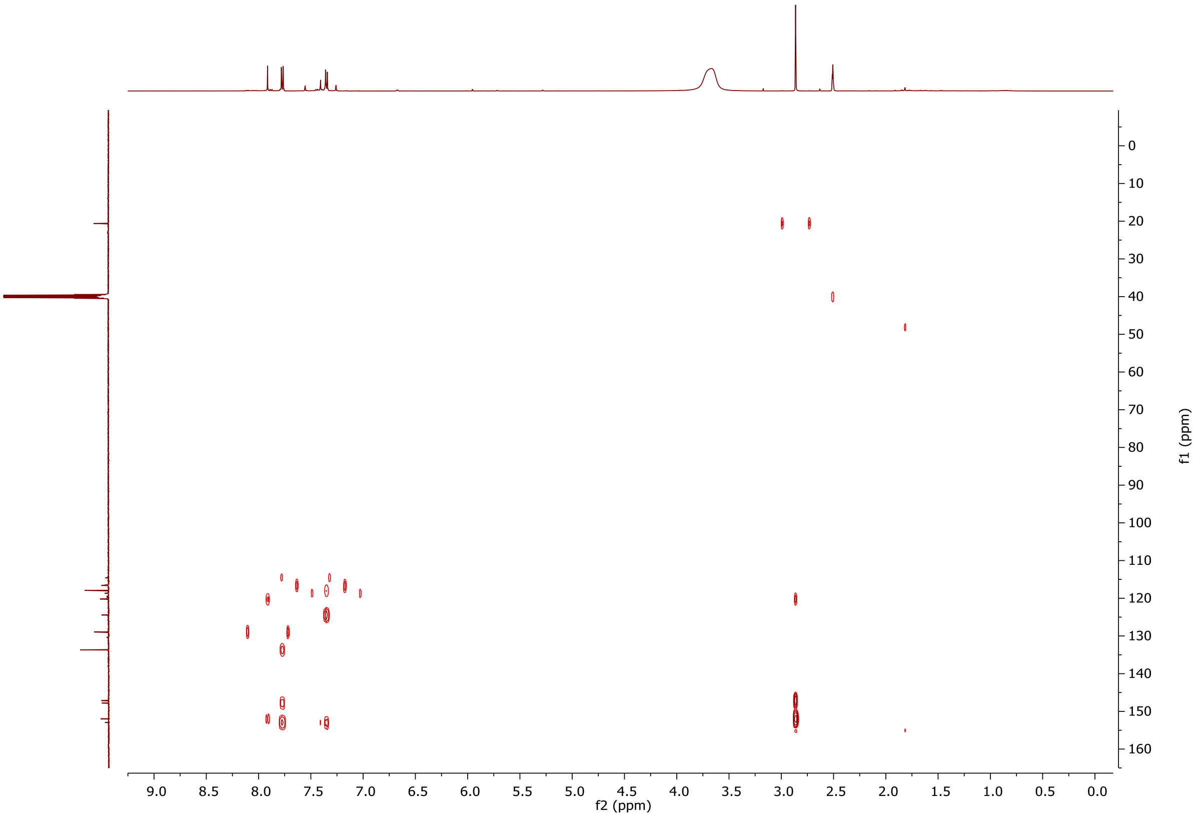

S59: HRMS of compound **11**

Griffith UNIVERSITY

High Resolution Mass Spectrum

Analysis Info

Analysis Name	D:\Data\KahYean\20200508\RAD859000001.d
Method	DirectInfusion_2018_pos.m
Sample Name	RAD859
Comment	

Acquisition Date 5/12/2020 11:16:52 AM

Instrument maXis II ETD 1823391.22321

+MS, 0.1min #4

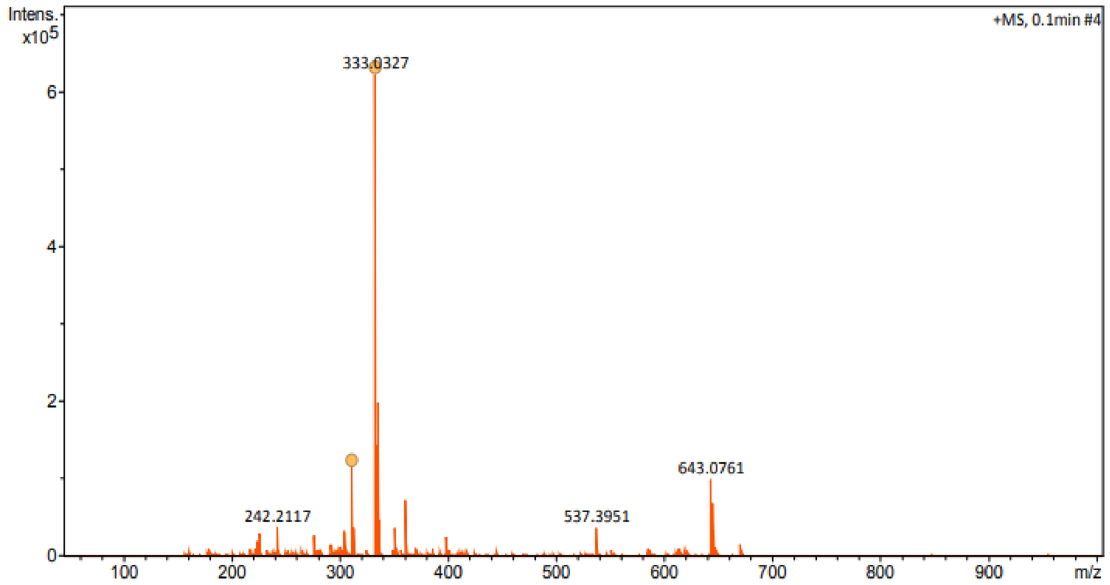

Meas. m/z	#	Ion Formula	m/z	err [ppm]	mSigma	# mSigma	Score	rdb	e^- Conf	N-Rule
311.0508	1	C13H10ClF2N4O	311.0506	-0.7	40.9	1	100.00	9.5	even	ok
333.0327	1	C13H9ClF2N4NaO	333.0325	-0.7	39.6	1	100.00	9.5	even	ok

S60: NMR data of compound **12** in DMSO-d_6[a]

Pos.	δ_H, mult. (J in Hz), int.	δ_C, mult. (J in Hz)	COSY	HMBC	ROESY
3		146.8, s			
5		141.6, s			
6	7.52, s, 1H	108.9, s		5, 8	21
8		145.1, s			
9		146.4, s			
10		124.4, s			
11	7.76, m, 1H	132.4, s	12	3, 13,15	12
12	7.28, m, 1H	117.7, s	11	10,14	11
13		152.0, t (3.2)			
14	7.28, m, 1H	117.7, s	15	10, 12	15
15	7.76, m, 1H	132.4, s	14	3, 11, 13	14
17	7.35, t (73.7), 1H	116.1, t (258.5)		13	
21	4.96, q (8.5), 2H	66.0, q (35.8)		5, 22	6
22		122.5, q (277.3)			
26	2.78, s	19.8, s		8, 9	

[a] Recorded at 500 MHz (^{1}H NMR) and 125 MHz (^{13}C NMR) at 25 °C.

S61: ^{1}H NMR spectrum of compound **12** in DMSO-d_6

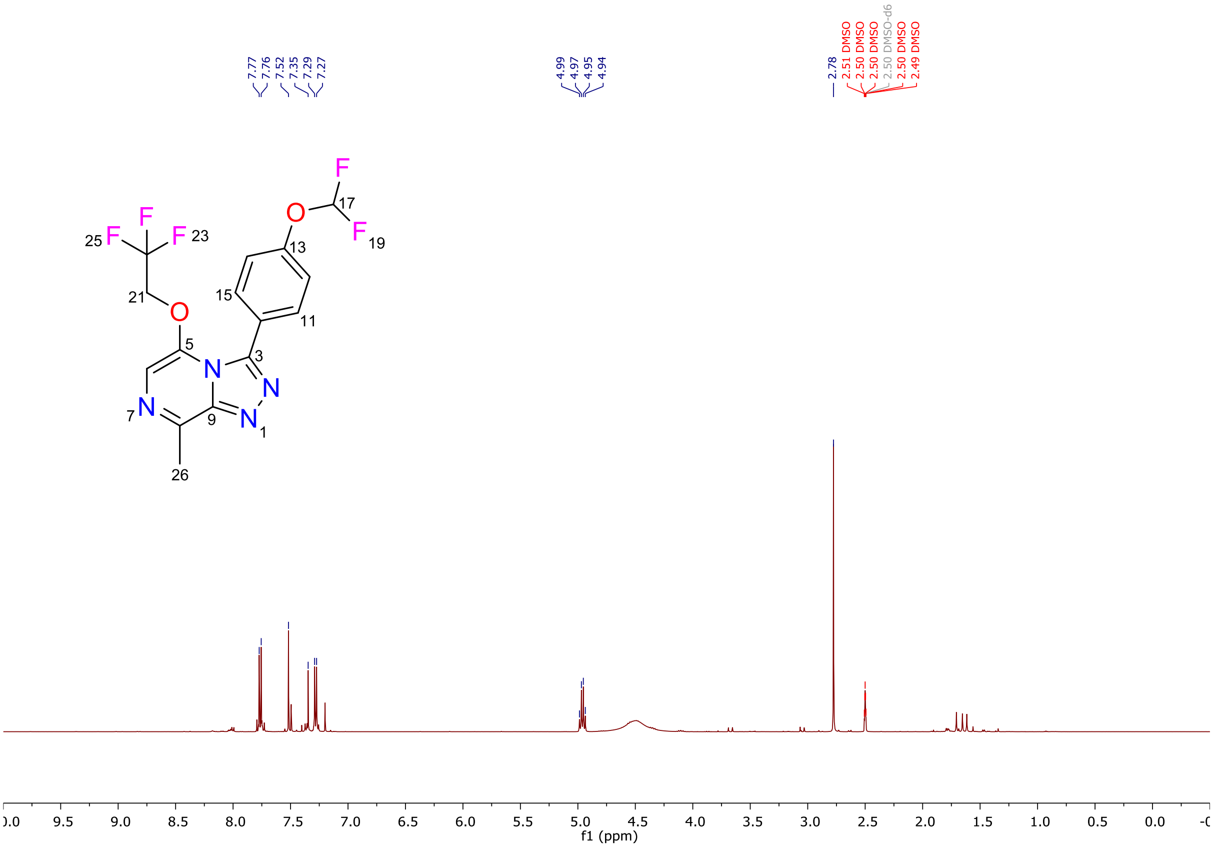

S62: ^{13}C NMR spectrum of compound **12** in DMSO-d_6

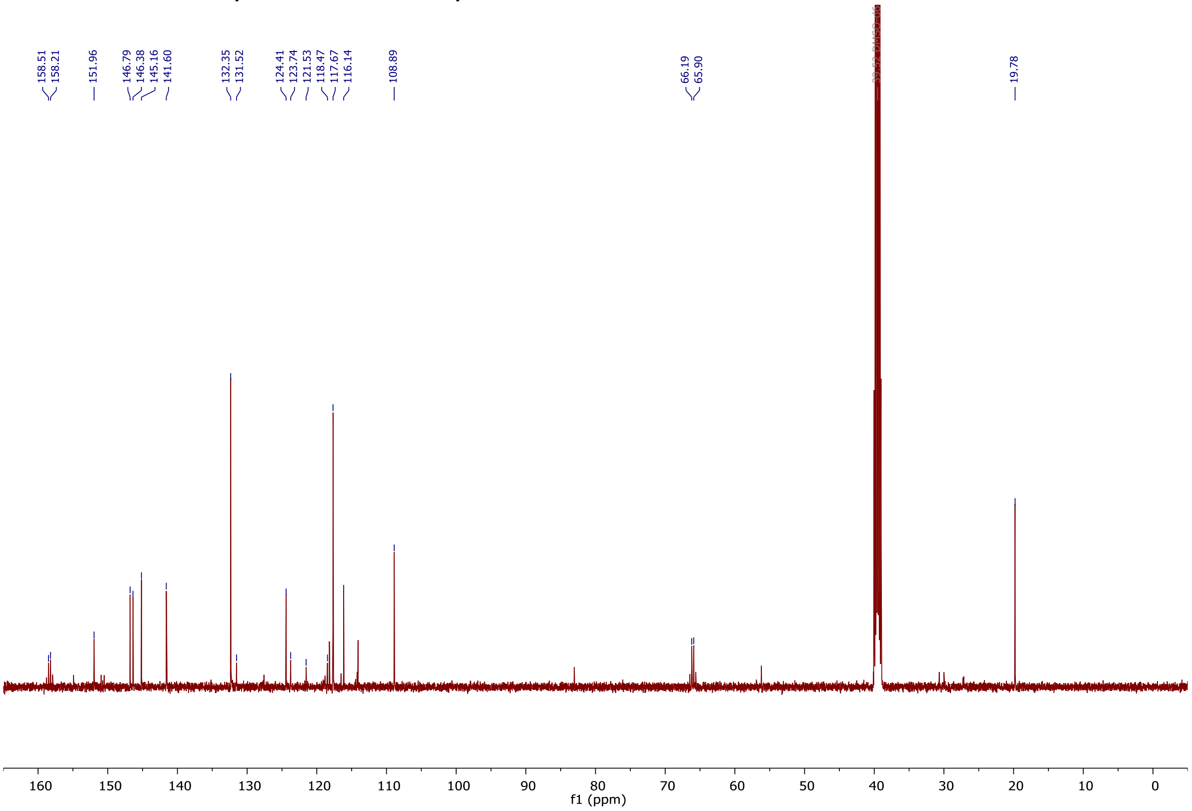

S63: COSY spectrum of compound **12** in DMSO-d_6

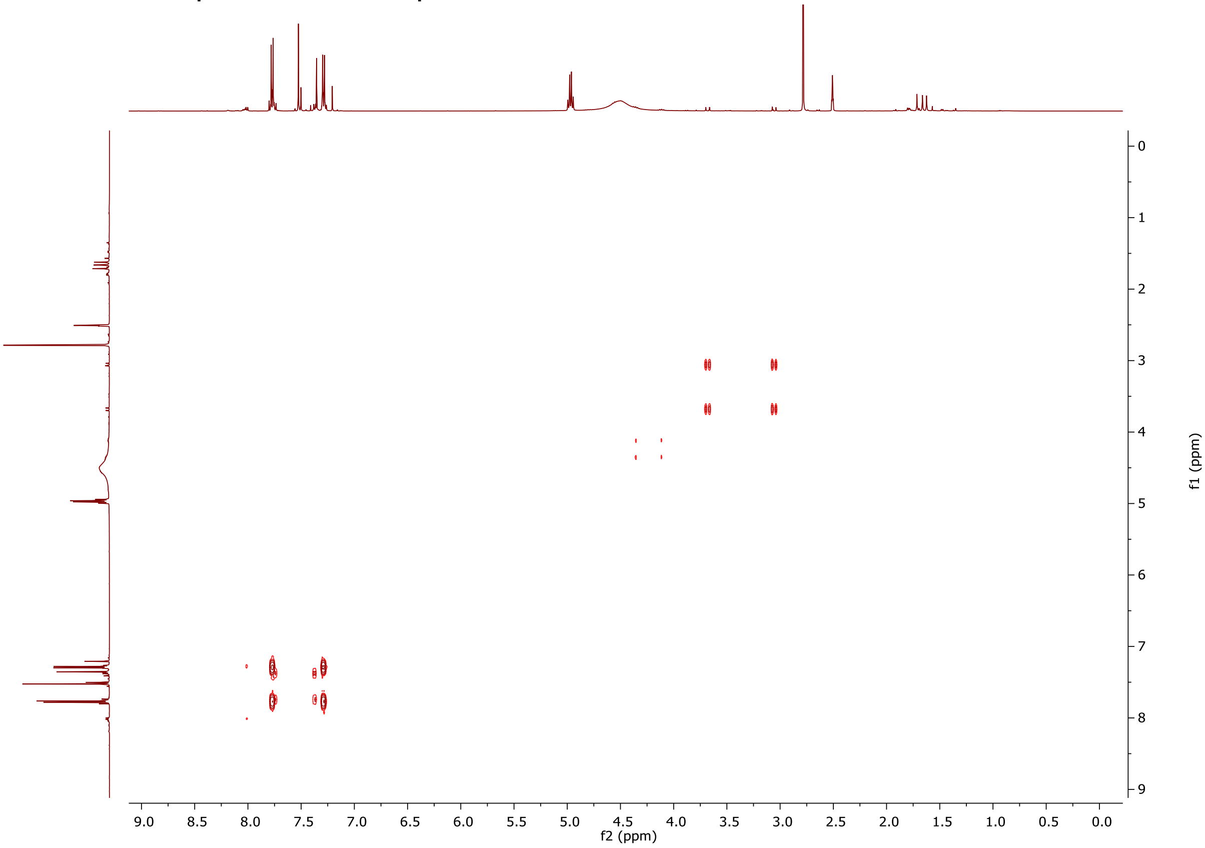

S64: HSQC spectrum of compound **12** in DMSO-d_6

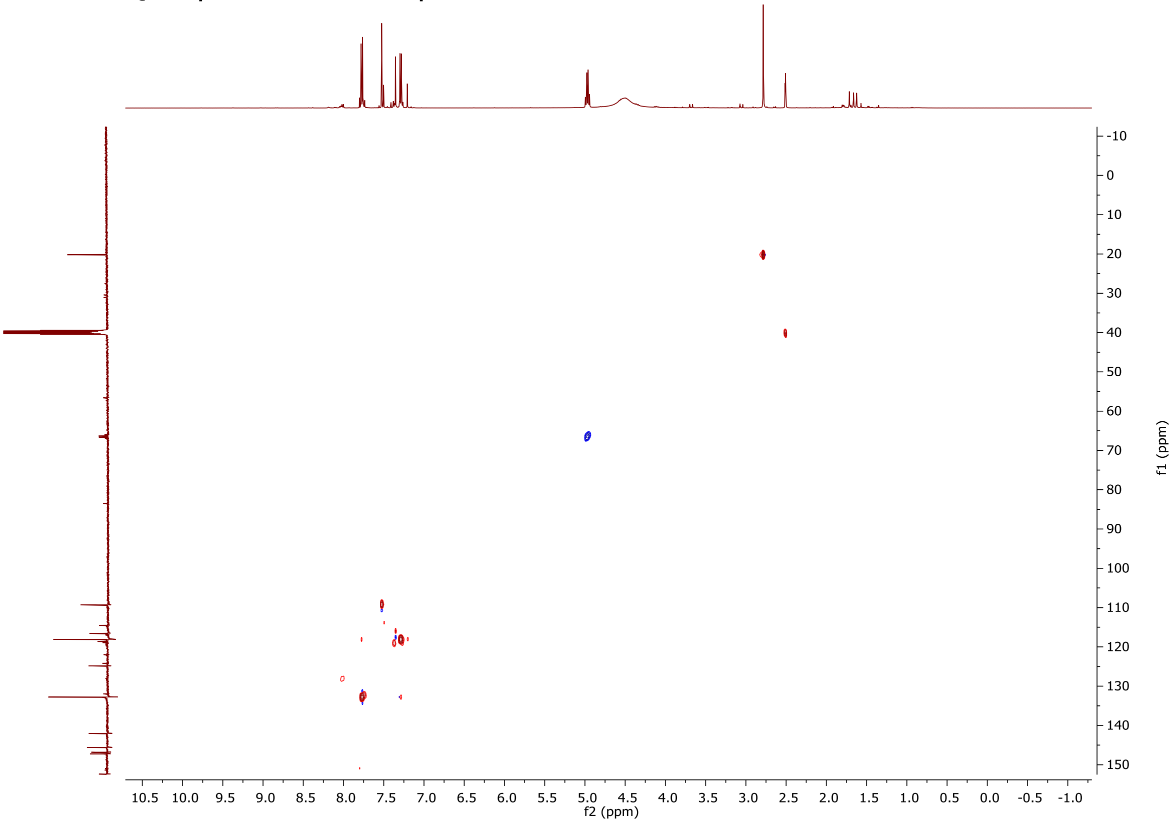

S65: HMBC spectrum of compound **12** in DMSO-d_6

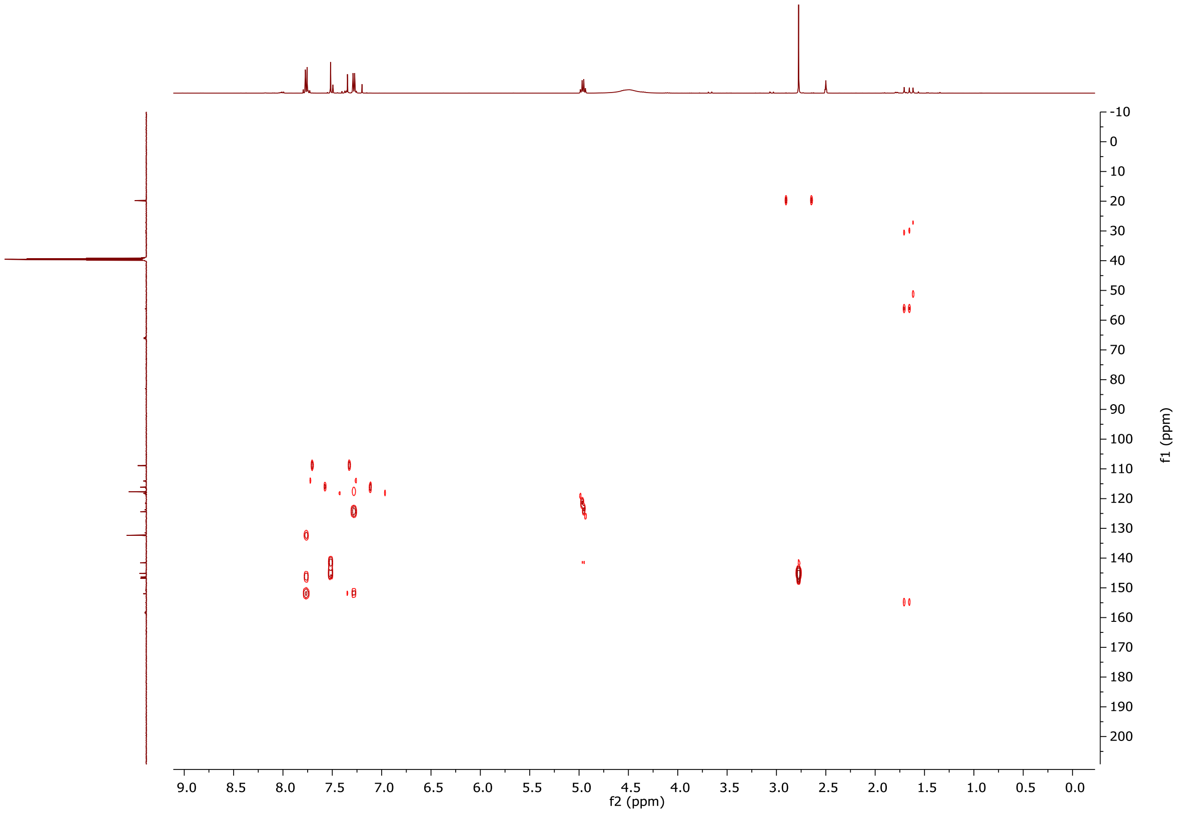

S66: ROESY spectrum of compound **12** in DMSO-d_6

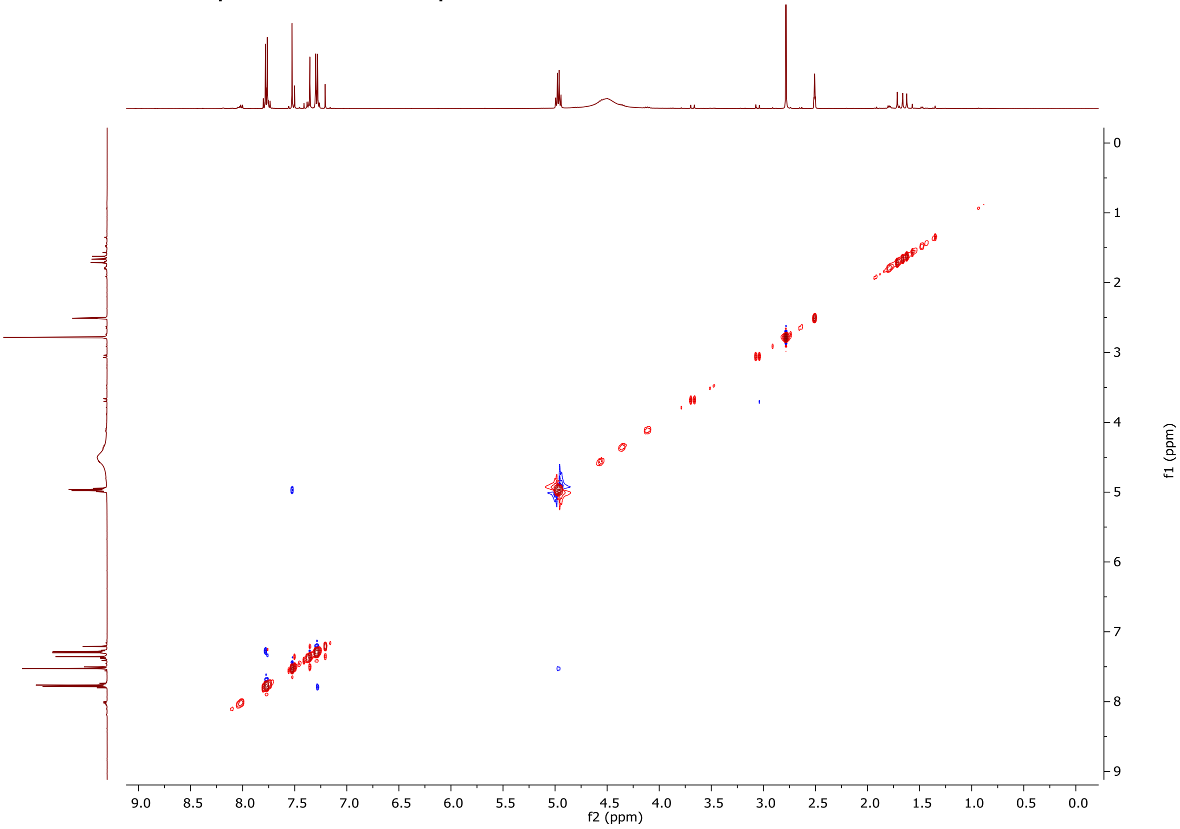

S67: HRMS of compound **12**

Griffith UNIVERSITY

High Resolution Mass Spectrum

Analysis Info

Analysis Name	D:\Data\KahYean\20200904\RAD866000001.d
Method	DirectInfusion_2018_pos.m
Sample Name	RAD866
Comment	

Acquisition Date 9/4/2020 11:41:46 AM

Instrument maXis II ETD 1823391.22321

+MS, 0.3min #18

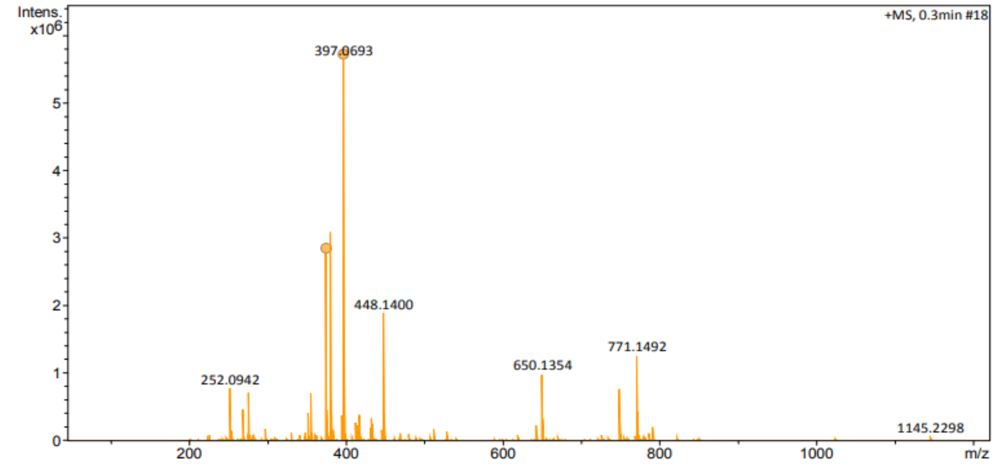

Meas. m/z	#	Ion Formula	m/z	err [ppm]	mSigma	# mSigma	Score	rdb	e⁻ Conf	N-Rule
375.0874	1	C15H12F5N4O2	375.0875	0.3	5.5	1	100.00	9.5	even	ok
397.0693	1	C15H11F5N4NaO2	397.0694	0.4	5.0	1	100.00	9.5	even	ok

S68: NMR data for compound **13** in DMSO-d_6[a]

Pos.	δ_H, mult. (J in Hz), int.	δ_C, mult. (J in Hz)	COSY	HMBC	ROESY
3		146.3, s			
5		142.8, s			
6	7.39, s, 1H	107.9, s		5, 8	
8		143.2, s			
9		146.8, s			
10		124.8, s			
11	7.74, m, 1H	132.6, s	12	3, 13, 15	12
12	7.29, m, 1H	117.6, s	11	10,14	11
13		152.0, t (3.2)			
14	7.29, m, 1H	117.6, s	15	10, 12	15
15	7.74, m, 1H	132.6, s	14	3, 11, 13	14
17	7.36, t (73.7), 1H	116.2, t (258.2)		13	
21	4.42, t (6.5), 2H	71.0, s	22	5, 22, 23	6, 22
22	2.86, t (6.5), 2H	33.9, s	21	21, 23, 24, 28	24, 28
23		137.4, s			
24	6.90, m, 1H	128.7, s	25	22, 26, 28	25
25	7.17, m, 1H	128.2, s	24, 26	23, 27	24, 26
26	7.16, m, 1H	126.4, s	25, 27	24, 28	25, 27
27	7.17, m, 1H	128.2, s	26, 28	23, 25	26, 28
28	6.90, m, 1H	128.7, s	27	22, 24, 26	27
29	2.72, s, 3H	19.7, s		6, 8, 9	

[a] Recorded at 500 MHz (^{1}H NMR) and 125 MHz (^{13}C NMR) at 25 °C.

S69: ^{1}H NMR spectrum of compound **13** in DMSO-d_6

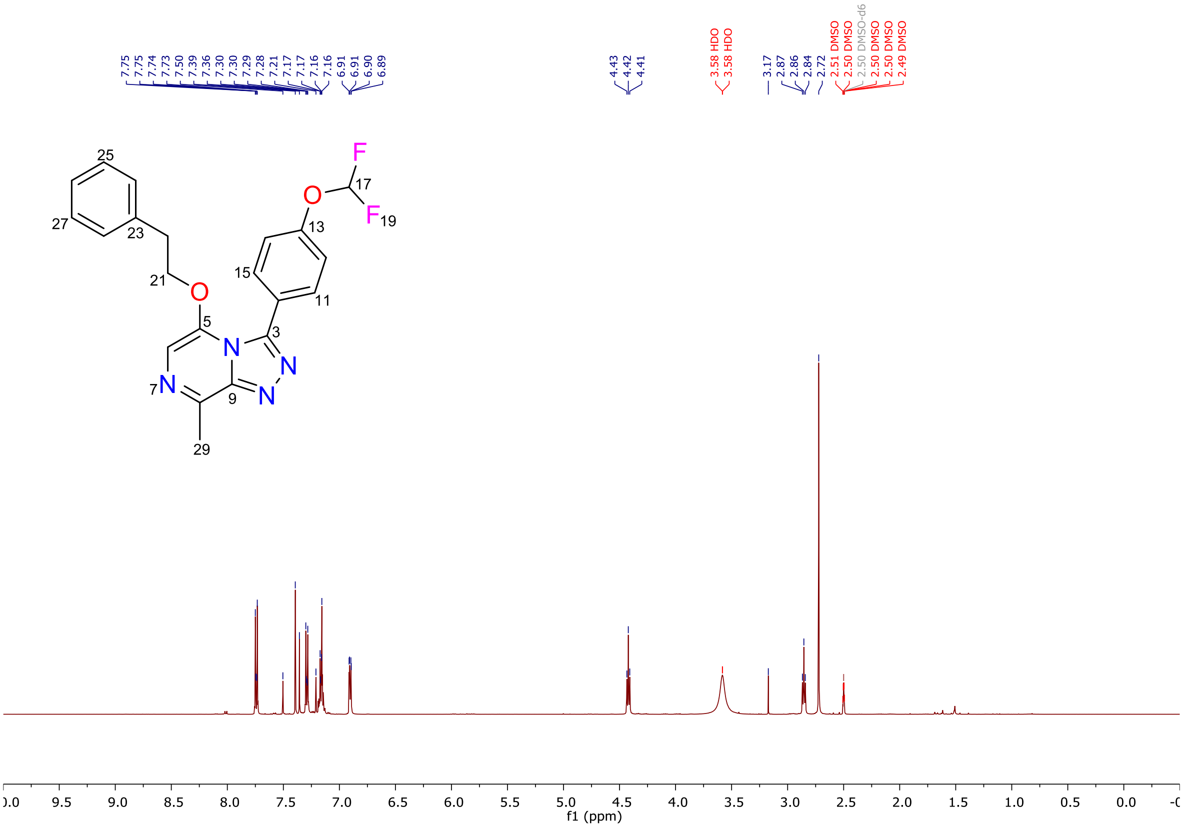

S70: ^{13}C NMR spectrum of compound **13** in DMSO-*d*$_6$

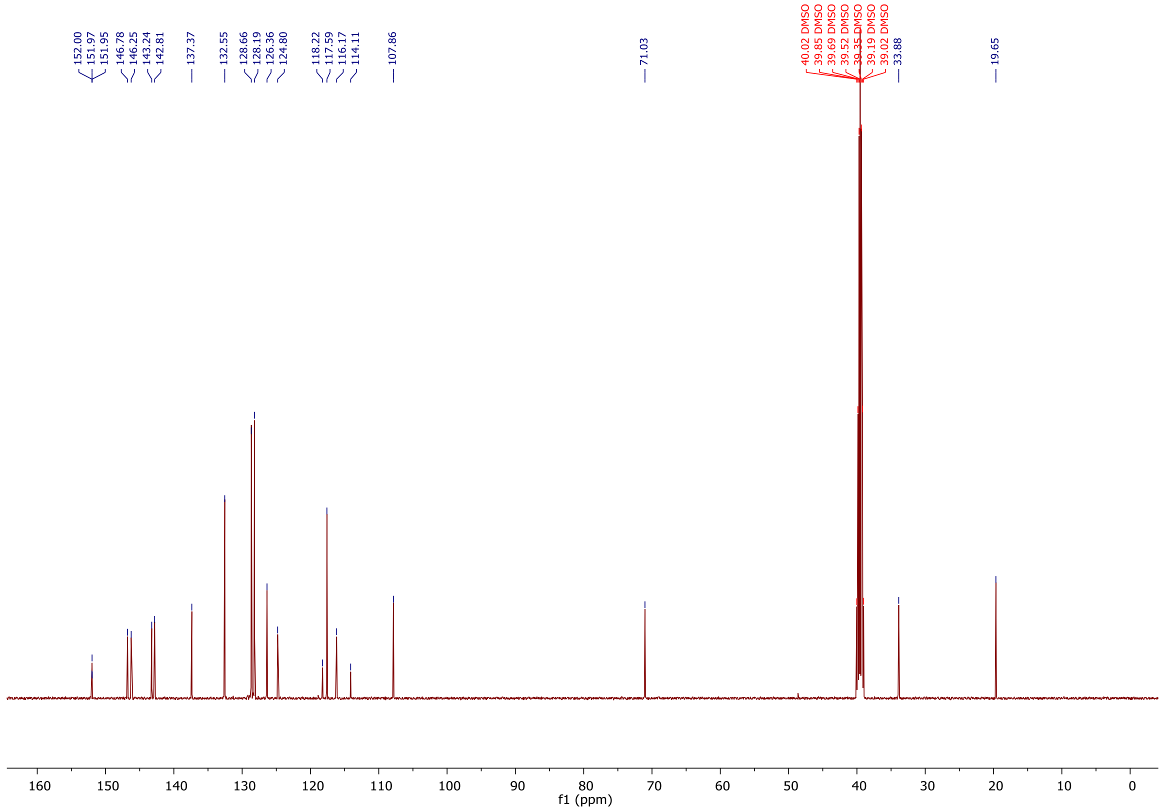

S71: COSY spectrum of compound **13** in DMSO-*d*$_6$

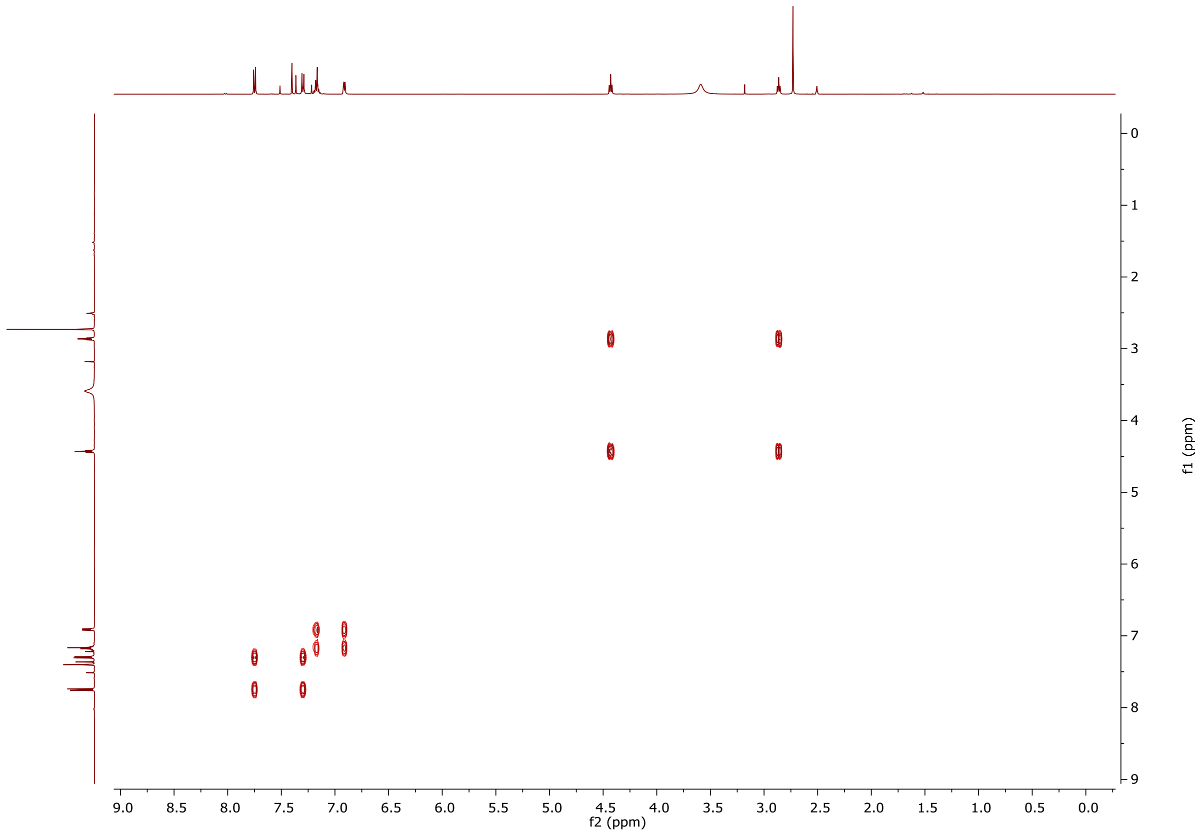

S72: HSQC spectrum of compound **13** in DMSO-d_6

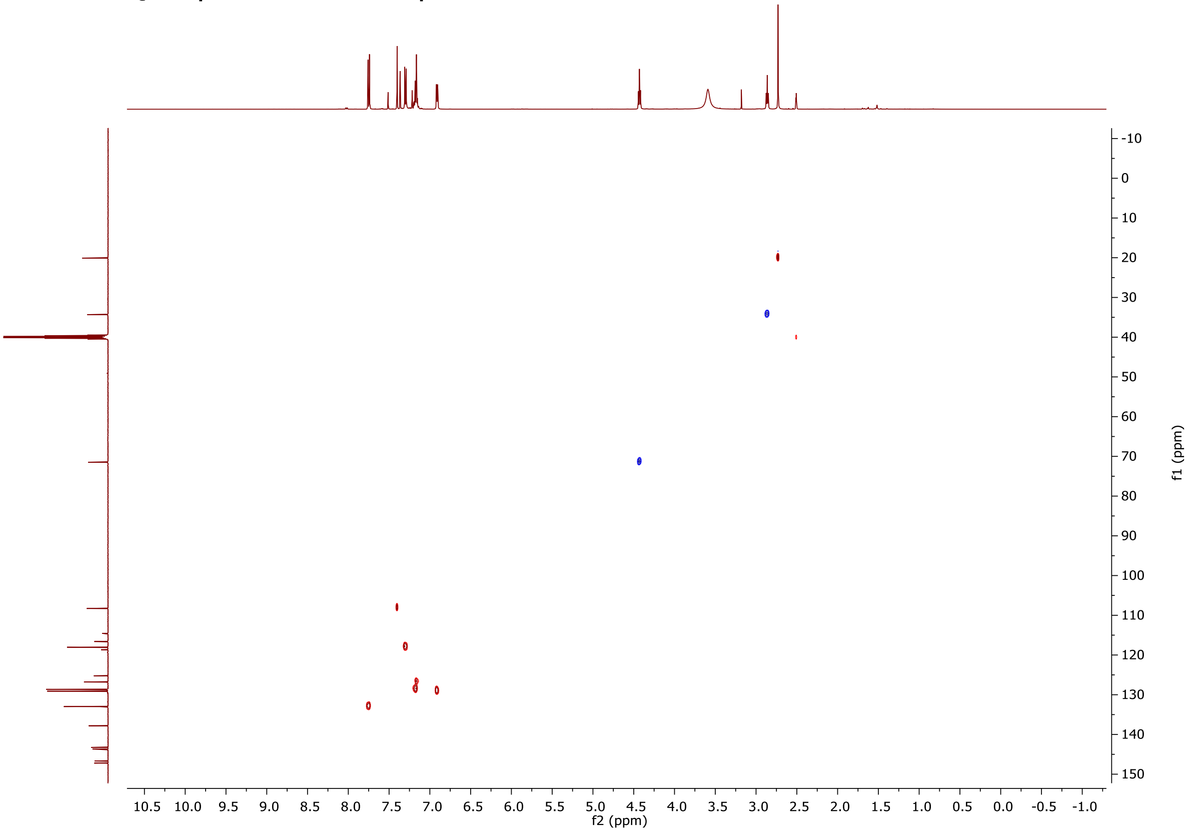

S73: HMBC spectrum of compound **13** in DMSO-d_6

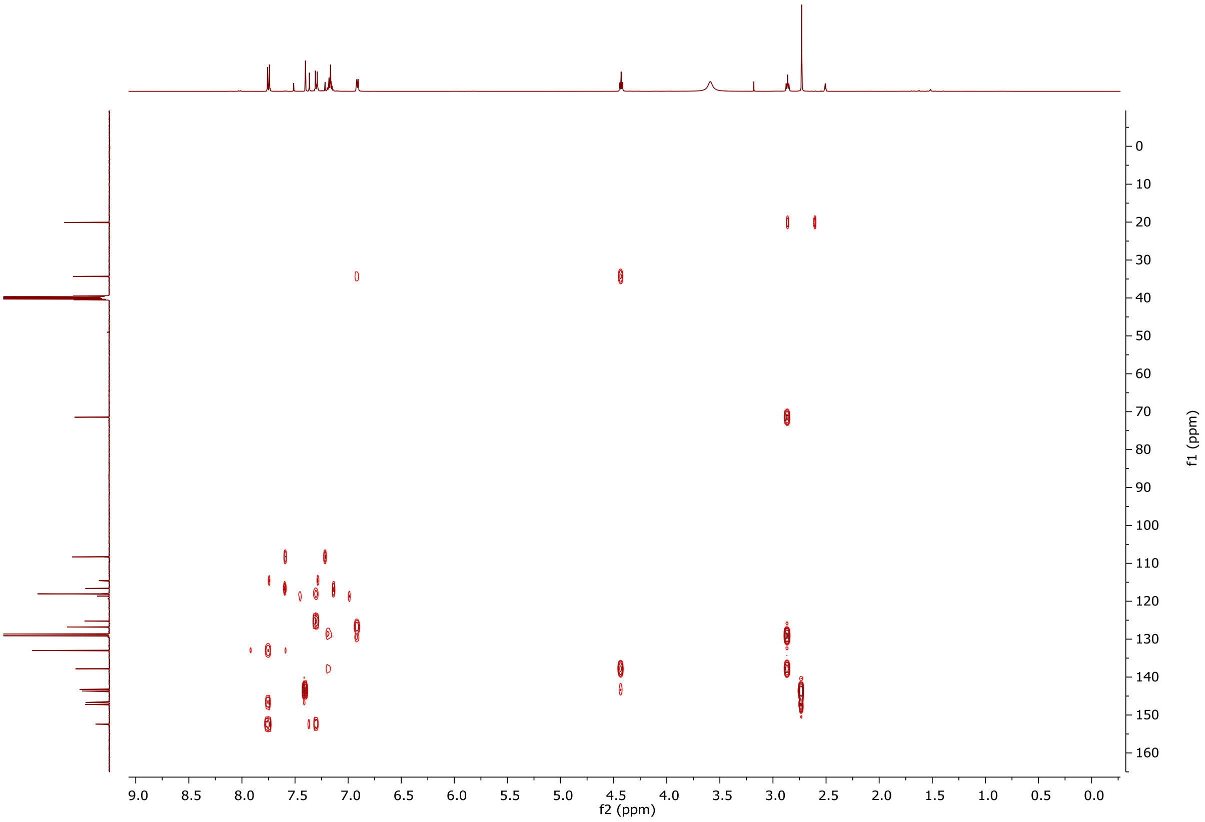

S74: ROESY spectrum of compound **13** in DMSO-d_6

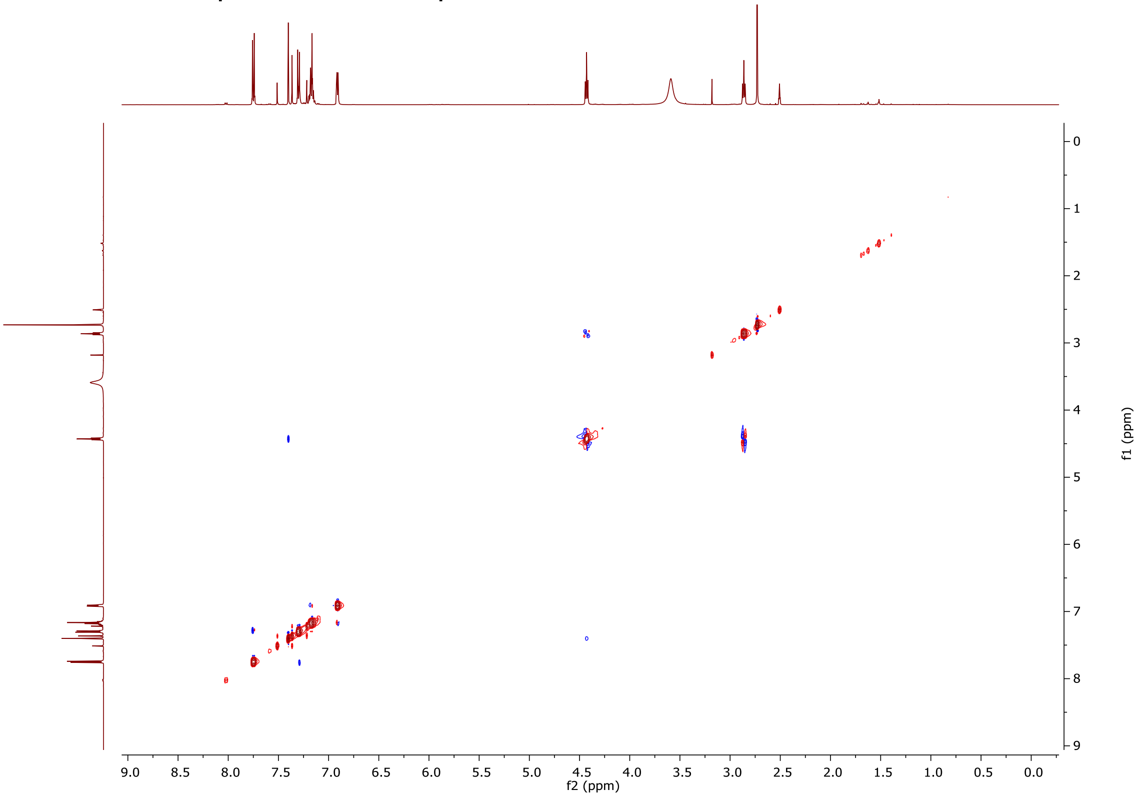

S75: HRMS of compound **13**

Griffith UNIVERSITY

High Resolution Mass Spectrum

Analysis Info

Analysis Name D:\Data\KahYean\20200904\RAD865000002.d
Method DirectInfusion_2018_pos.m
Sample Name RAD865
Comment

Acquisition Date 9/4/2020 11:35:55 AM
Instrument maXis II ETD 1823391.22321

+MS, 0.2-0.3min #10-16

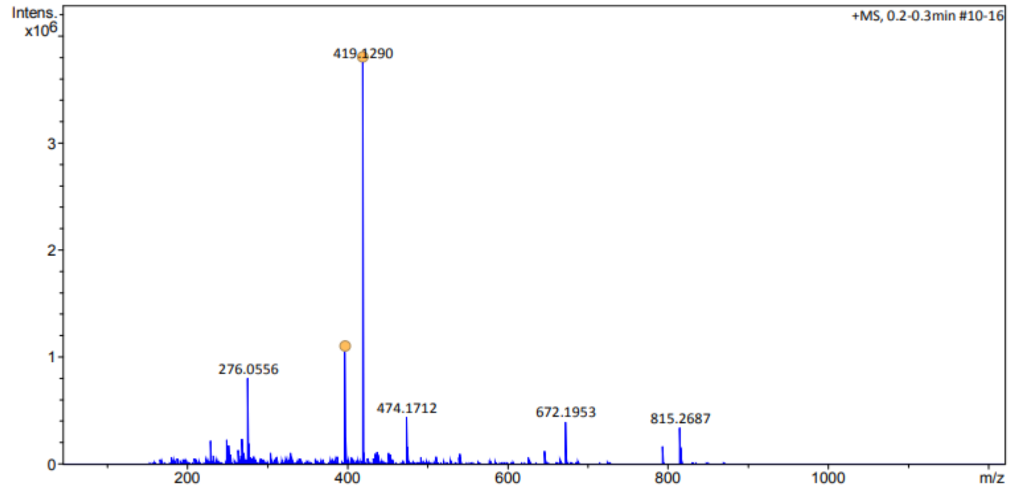

Meas. m/z	#	Ion Formula	m/z	err [ppm]	mSigma	# mSigma	Score	rdb	e⁻ Conf	N-Rule
397.1470	1	C21H19F2N4O2	397.1471	0.0	5.3	1	100.00	13.5	even	ok
419.1290	1	C21H18F2N4NaO2	419.1290	0.0	9.3	1	100.00	13.5	even	ok

S76: NMR data of compound **14** in DMSO-d_6[a]

Position	δ_H, mult. (*J* in Hz), int.	δ_C, mult. (*J* in Hz)	COSY	HMBC
3		147.5, s		
5		126.3, s		
6	8.30, s, 1H	127.8, s		5, 8
8		137.4, q (37.9)		
9		143.4, s		
10		125.6, s		
11	7.75, m, 1H	133.2, s	12	3, 13,15
12	7.67, m, 1H	128.0, s	11	10,14
13		135.8, s		
14	7.67, m, 1H	128.0, s	15	10, 12
15	7.75, m, 1H	133.2, s	14	3, 11, 13
18		119.9, q (275.1)		

[a] Recorded at 500 MHz (^{1}H NMR) and 125 MHz (^{13}C NMR) at 25 °C.

S77: ^{1}H NMR Spectrum of compound **14** in DMSO-d_6

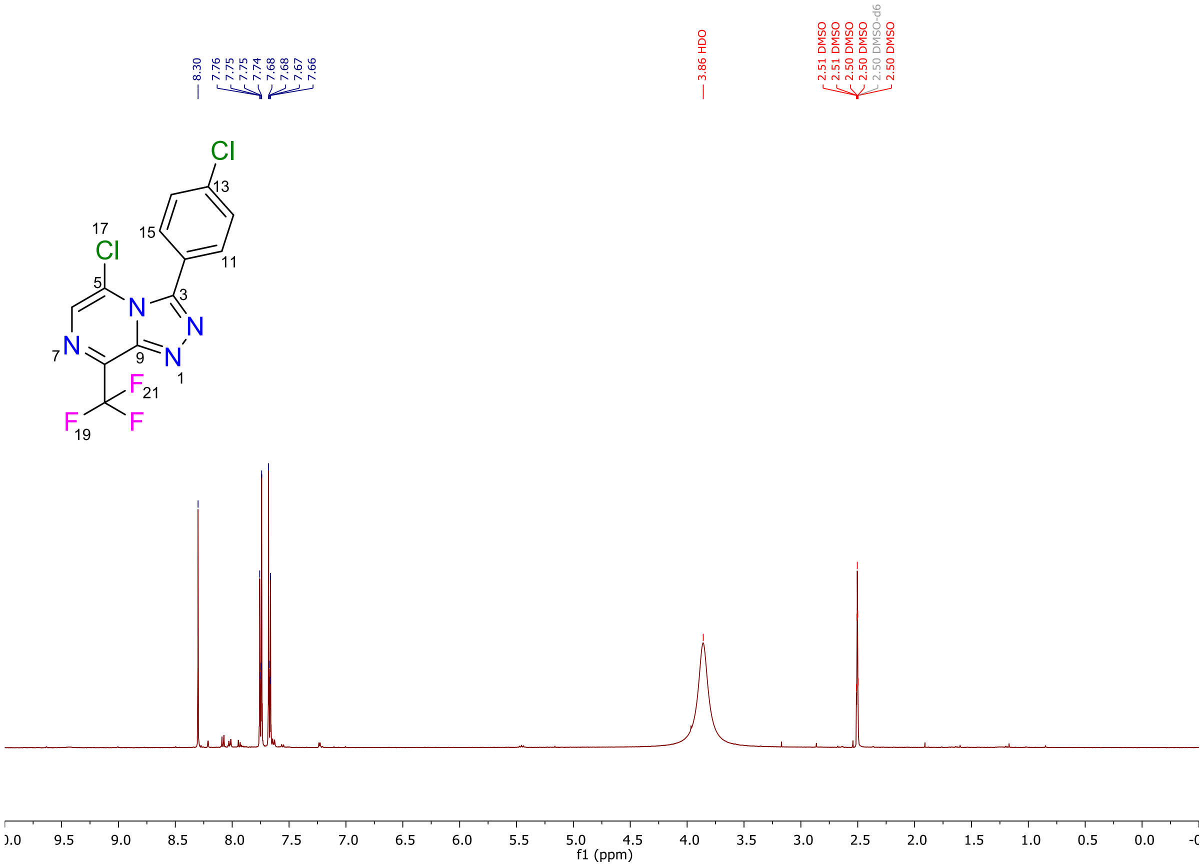

S78: ^{13}C NMR spectrum of compound **14** in DMSO-d_6

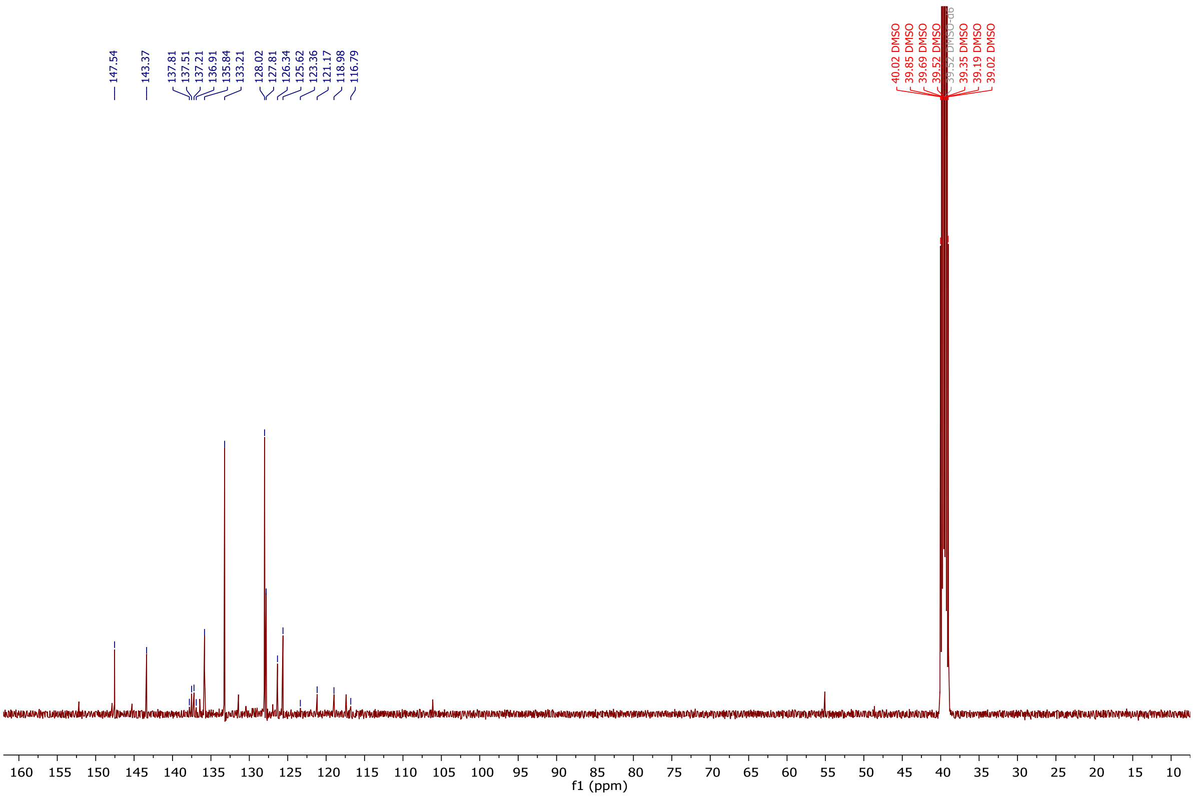

S79: COSY spectrum of compound **14** in DMSO-d_6

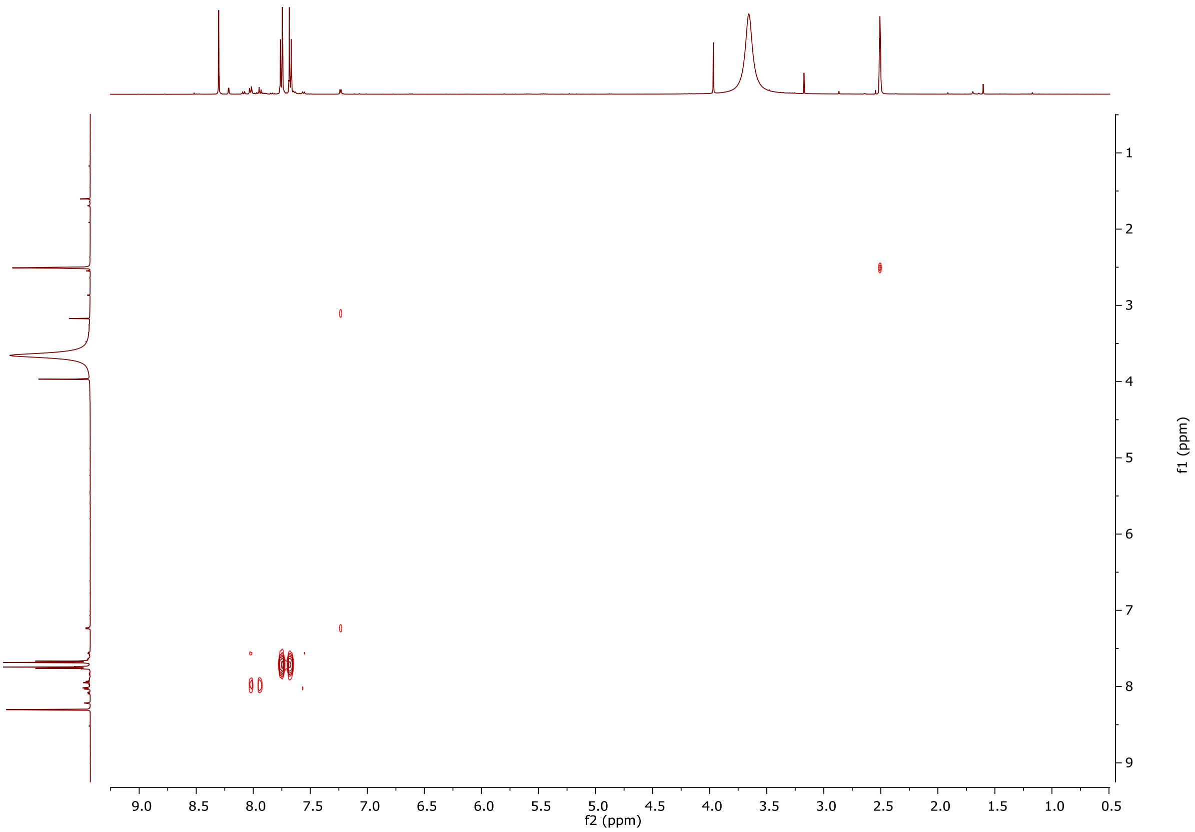

S80: HSQC spectrum of compound **14** in DMSO-d_6

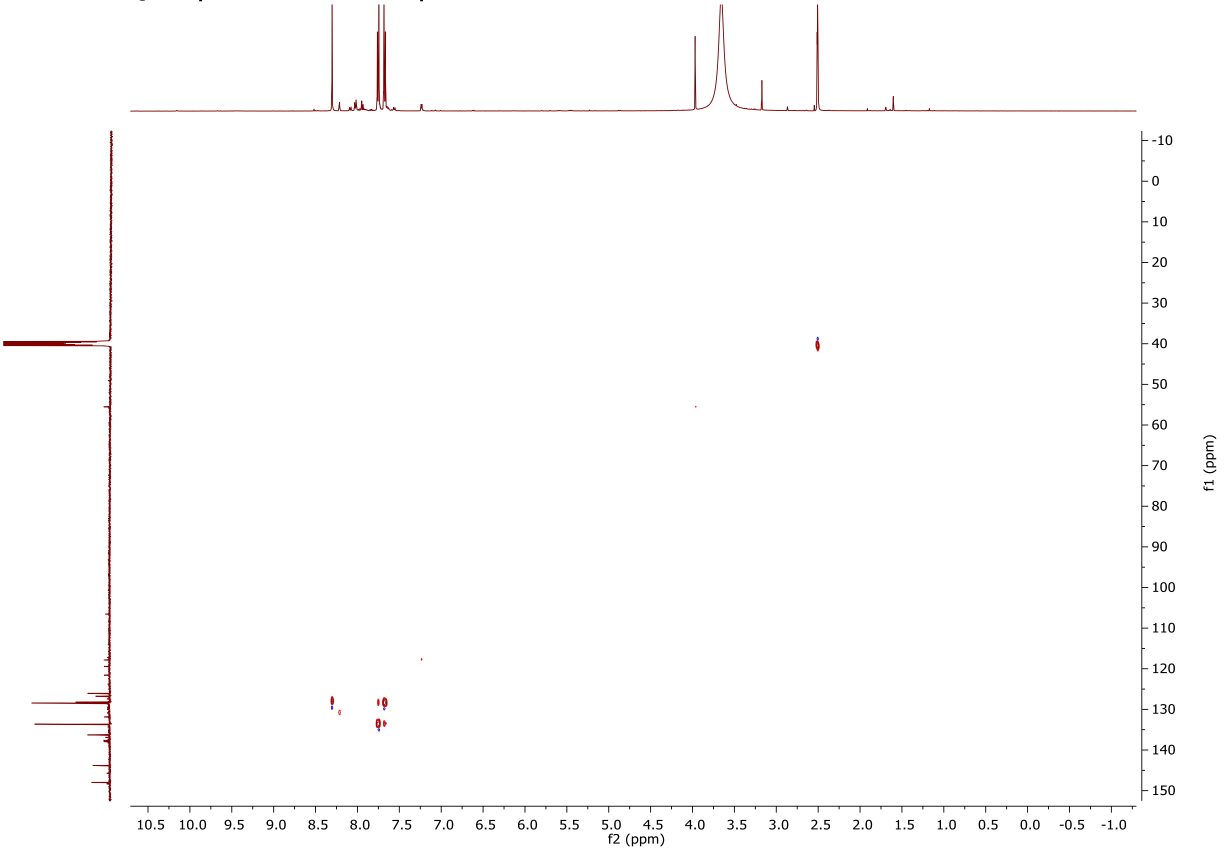

S81: HMBC spectrum of compound **14** in DMSO-d_6

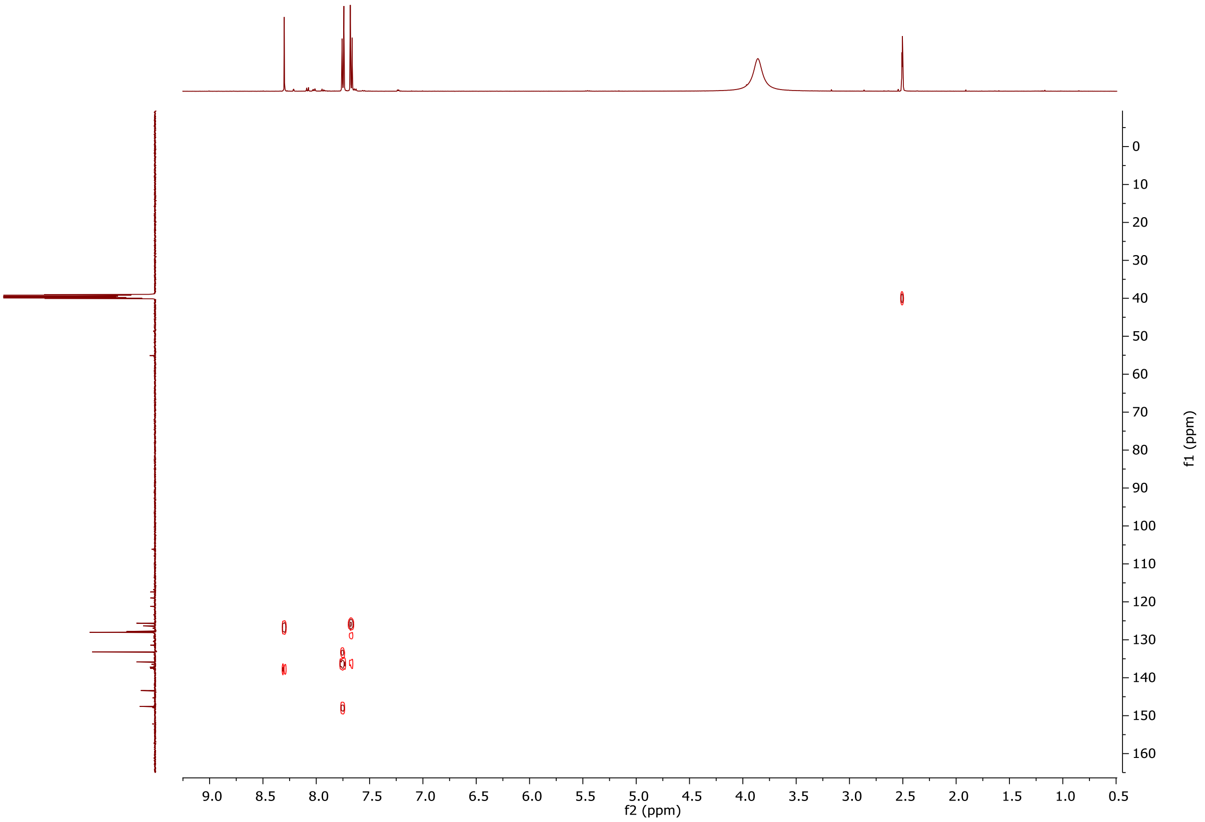

S82: HRMS of compound **14**

High Resolution Mass Spectrum

Analysis Info

Analysis Name	D:\Data\KahYean\20200904\RAD864000002.d		
Method	DirectInfusion_2018_pos.m	Acquisition Date	9/4/2020 10:42:02 AM
Sample Name	RAD864	Instrument	maXis II ETD 1823391.22321
Comment			

+MS, 0.2-0.3min #9-15

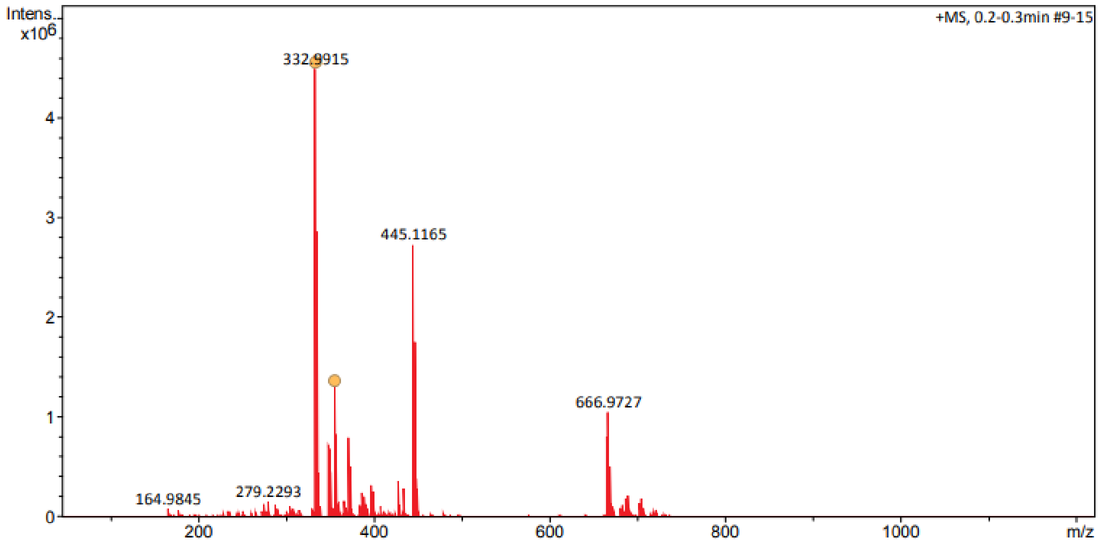

Meas. m/z	#	Ion Formula	m/z	err [ppm]	mSigma	# mSigma	Score	rdb	e⁻ Conf	N-Rule
332.9915	1	C12H6Cl2F3N4	332.9916	0.5	9.6	1	100.00	9.5	even	ok
	1	C12H8Cl2N4NaO2	332.9917	0.6	11.5	1	100.00	9.5	even	ok
354.9734	1	C12H5Cl2F3N4Na	354.9736	0.5	9.9	1	100.00	9.5	even	ok

S83: NMR data of compound **15** in DMSO-d_6[a]

Pos	δ_H, mult. (*J* in Hz), int.	δ_C, mult. (*J* in Hz)	COSY	HMBC
3		147.1, s		
5		124.2, s		
6	8.17, s, 1H	127.6, s		5, 8
8		144.9, t (31.3)		
9		143.9, s		
10		126.0, s		
11	7.75, m, 1H	133.2, s	12	3,13,15
12	7.65, m, 1H	127.9, s	11	10,14
13		135.7, s		
14	7.65, m, 1H	127.9, s	15	10, 12
15	7.75, m, 1H	133.2, s	14	3, 11, 13
18		119.9, t (240.2)		
19	2.25, t, (19.3), 3H	22.8, s		8, 19

[a] Recorded at 500 MHz (^1H NMR) and 125 MHz (^{13}C NMR) at 25 °C.

S84: ^1H NMR spectrum of compound **15** in DMSO-d_6

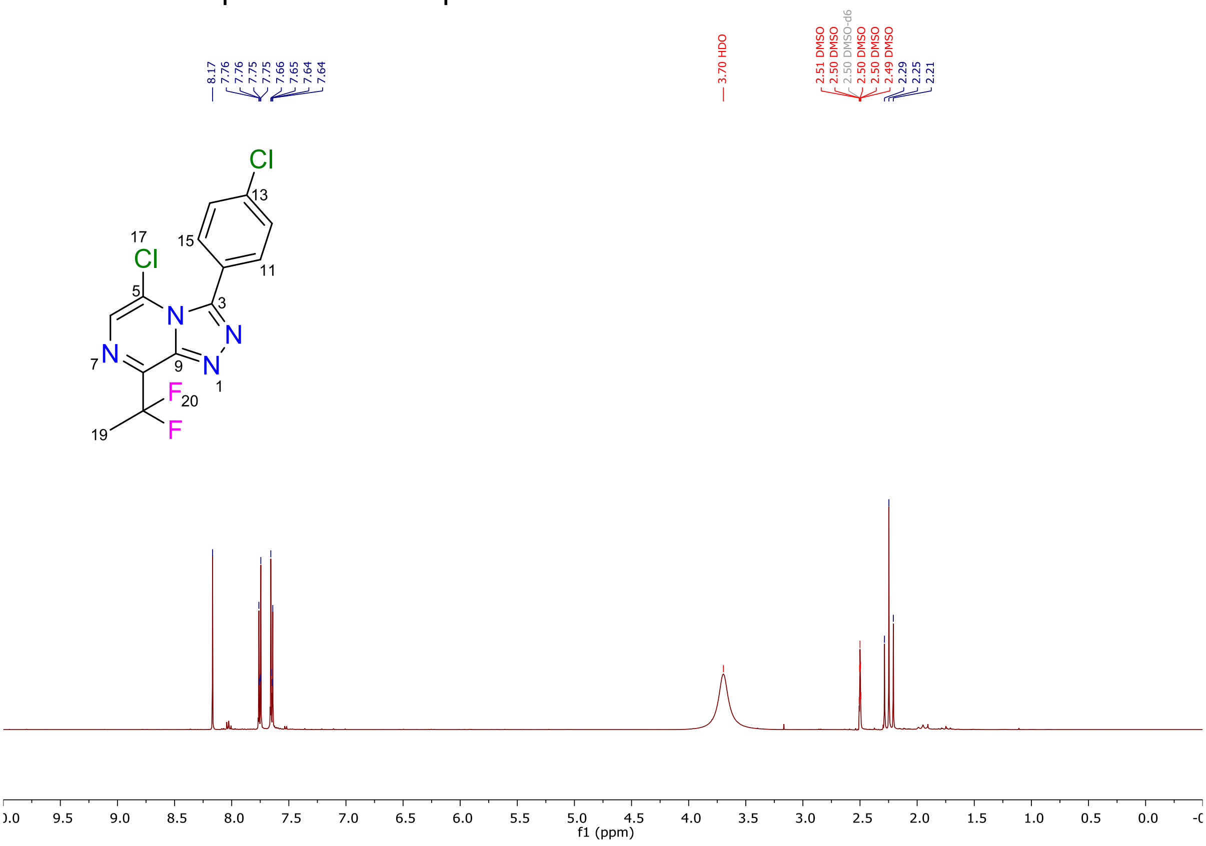

S85: ^{13}C NMR spectrum of compound **15** in DMSO-d_6

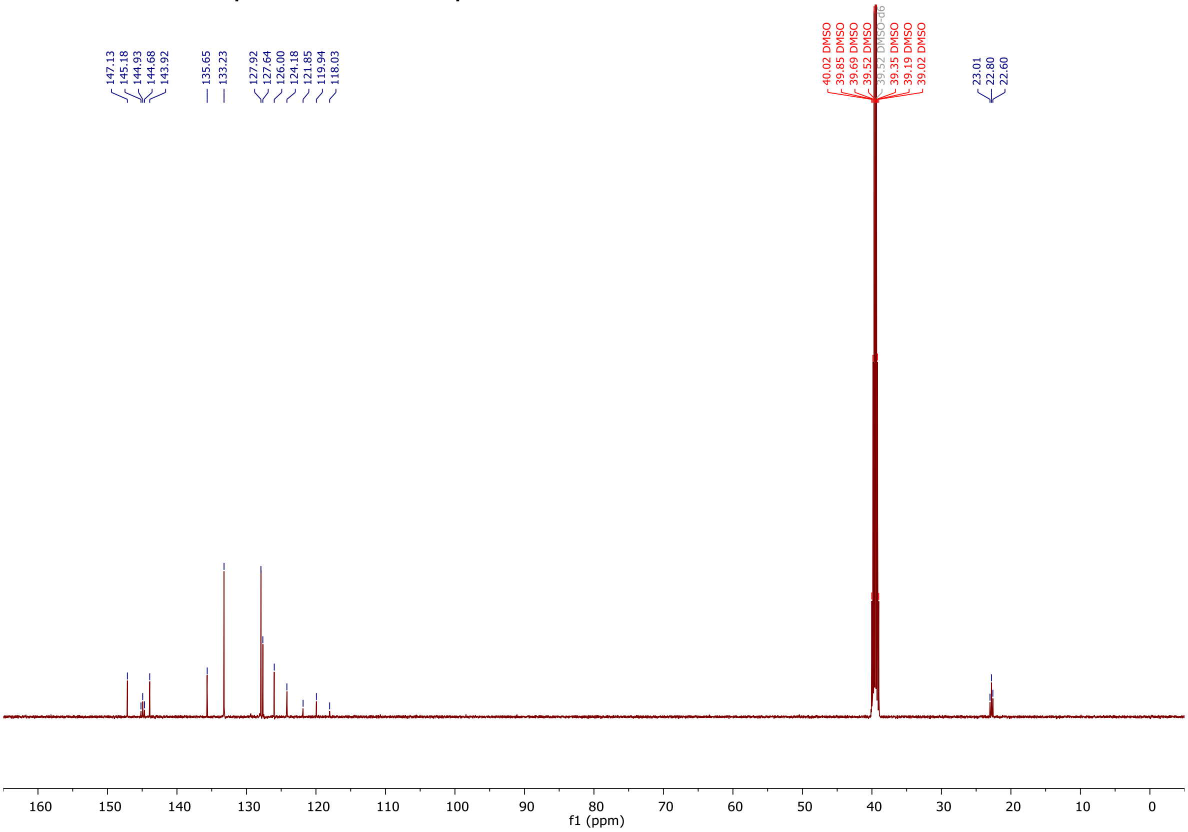

S86: COSY spectrum of compound **15** in DMSO-d_6

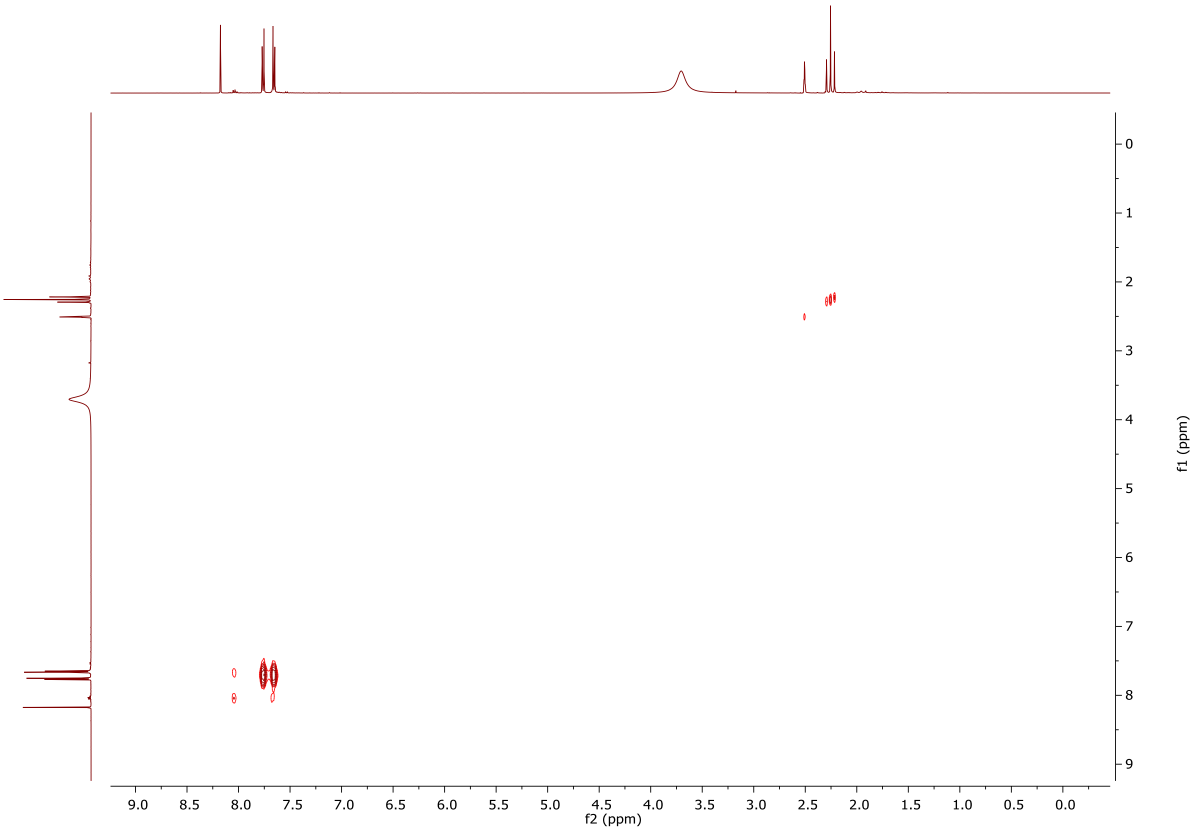

S87: HSQC spectrum of compound **15** in DMSO-d_6

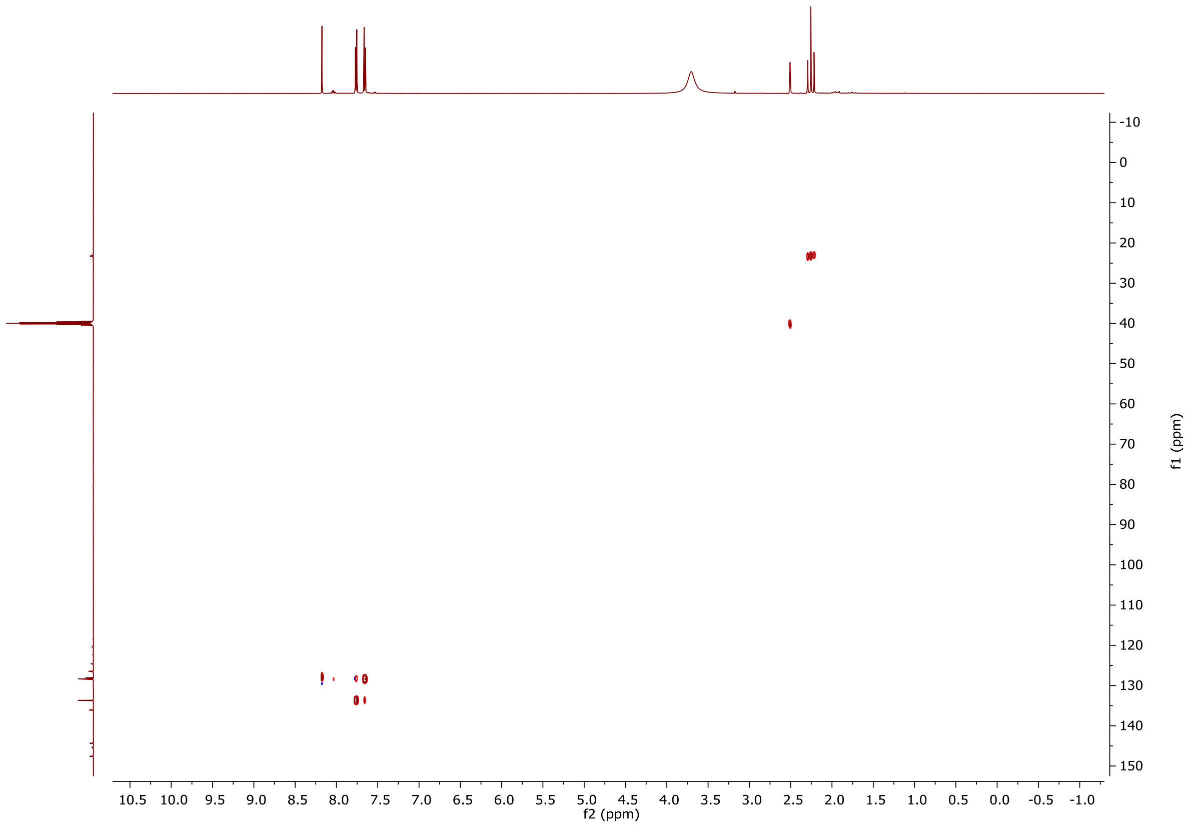

S88: HMBC spectrum of compound **15** in DMSO-d_6

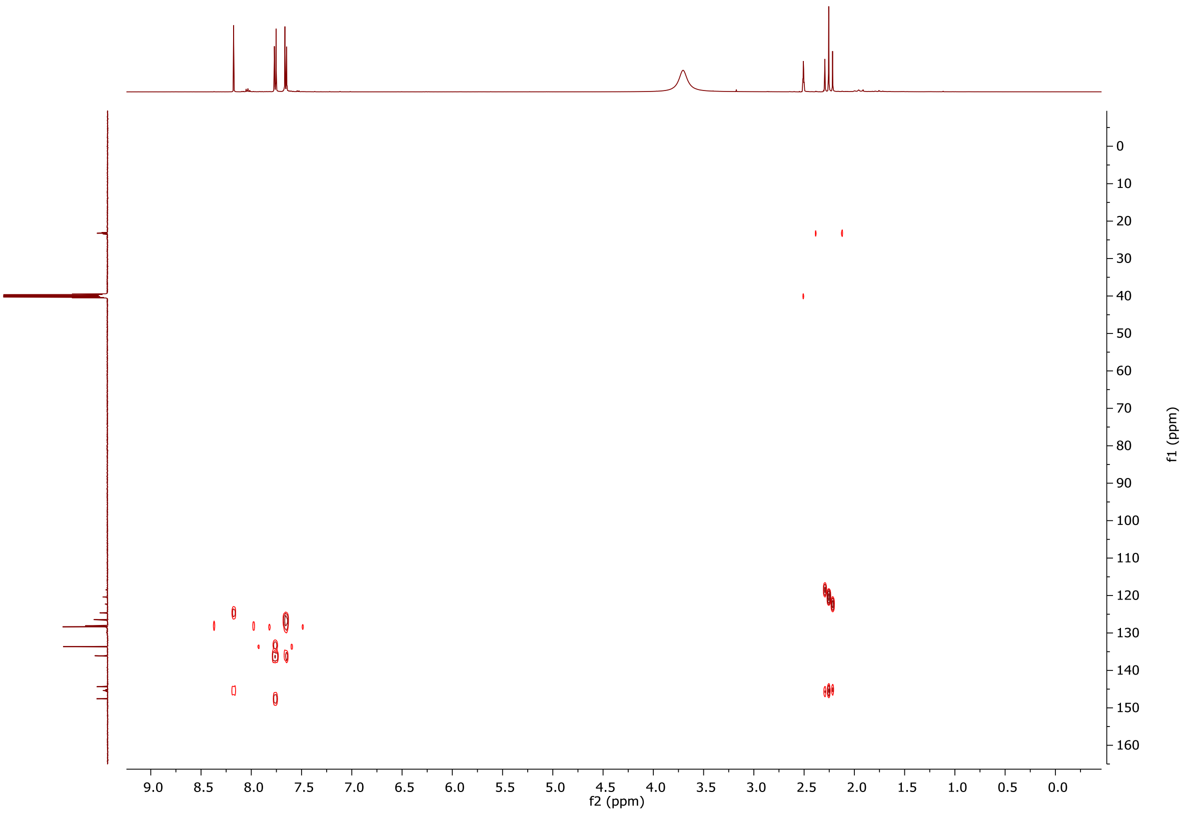

S89: HRMS of compound **15**

High Resolution Mass Spectrum

Analysis Info

Analysis Name D:\Data\KahYean\20200904\RAD867000001.d
Method DirectInfusion_2018_pos.m
Sample Name RAD867
Comment

Acquisition Date 9/4/2020 11:49:35 AM
Instrument maXis II ETD 1823391.22321

+MS, 0.2min #13

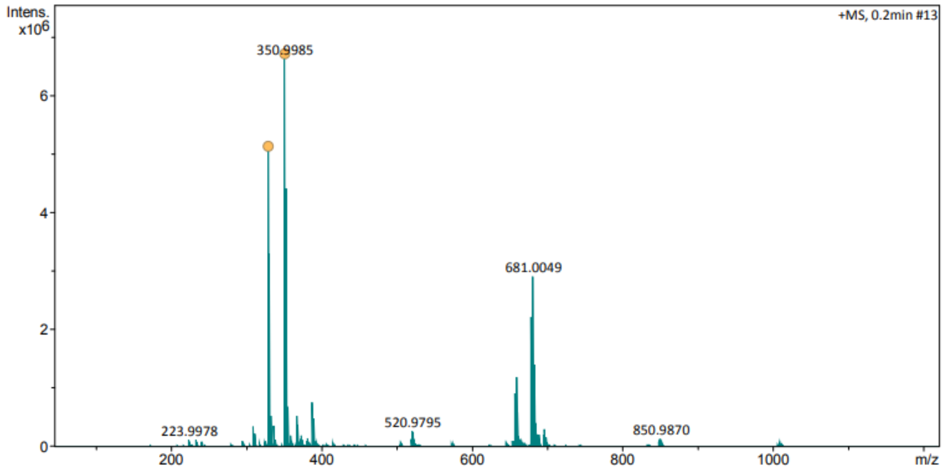

Meas. m/z	#	Ion Formula	m/z	err [ppm]	mSigma	# mSigma	Score	rdb	e¯ Conf	N-Rule
329.0166	1	C13H9Cl2F2N4	329.0167	0.4	6.3	1	100.00	9.5	even	ok
350.9985	1	C13H8Cl2F2N4Na	350.9986	0.4	7.6	1	100.00	9.5	even	ok

S90: NMR data of compound **16** in DMSO-d_6[a]

Pos.	δ_H, mult. (*J* in Hz), int	δ_C, mult. (*J* in Hz)	COSY	HMBC
3		146.4, s		
5	8.74, d (4.8), 1H	119.5, s	6	3, 6, 9
6	8.04, d (4.8), 1H	128.5, s	5	5, 8
8		146.4, t (30.9)		
9		142.6, s		
10		124.4, s		
11	7.98, m, 1H	130.3, s	12	3, 13,15
12	7.73, m, 1H	129.5, s	11	10, 13,14
13		135.6, s		
14	7.73, m, 1H	129.5, s	15	10, 12, 13
15	7.98, m, 1H	130.3, s	14	3 11, 13
17		120.0, t (239.7)		
18	2.25, t (19.2), 3H	22.7, t (25.6)		8, 17

[a] Recorded at 500 MHz (^{1}H NMR) and 125 MHz (^{13}C NMR) at 25 °C.

S91: ^{1}H NMR Spectrum of compound **16** in DMSO-d_6

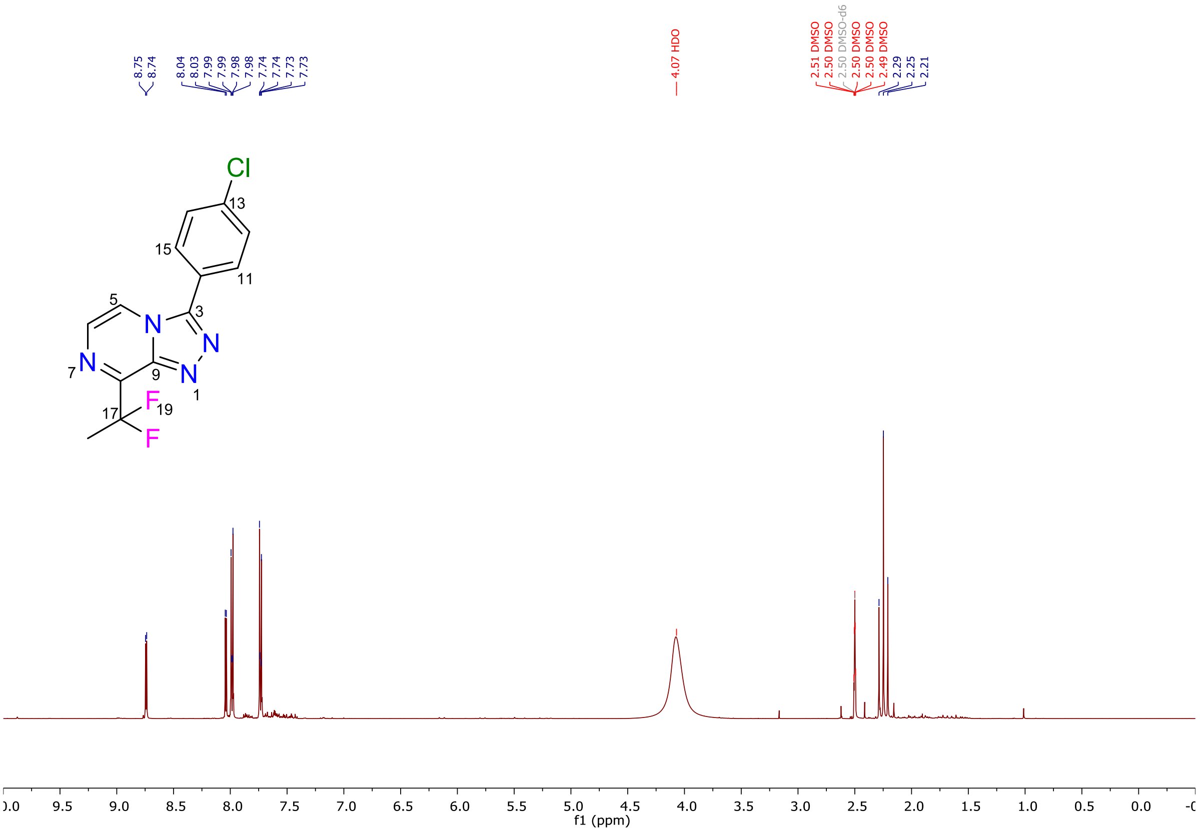

S92: ^{13}C NMR spectrum of compound **16** in DMSO-d_6

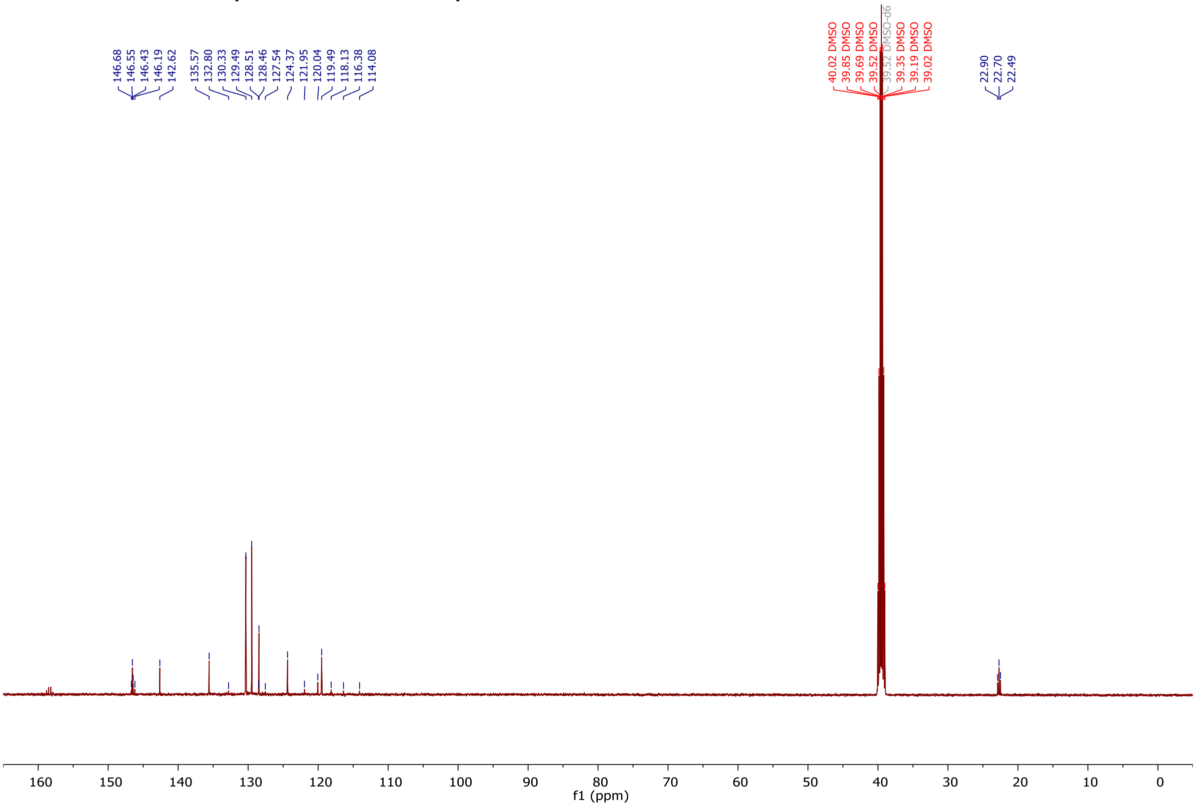

S93: COSY spectrum of compound **16** in DMSO-d_6

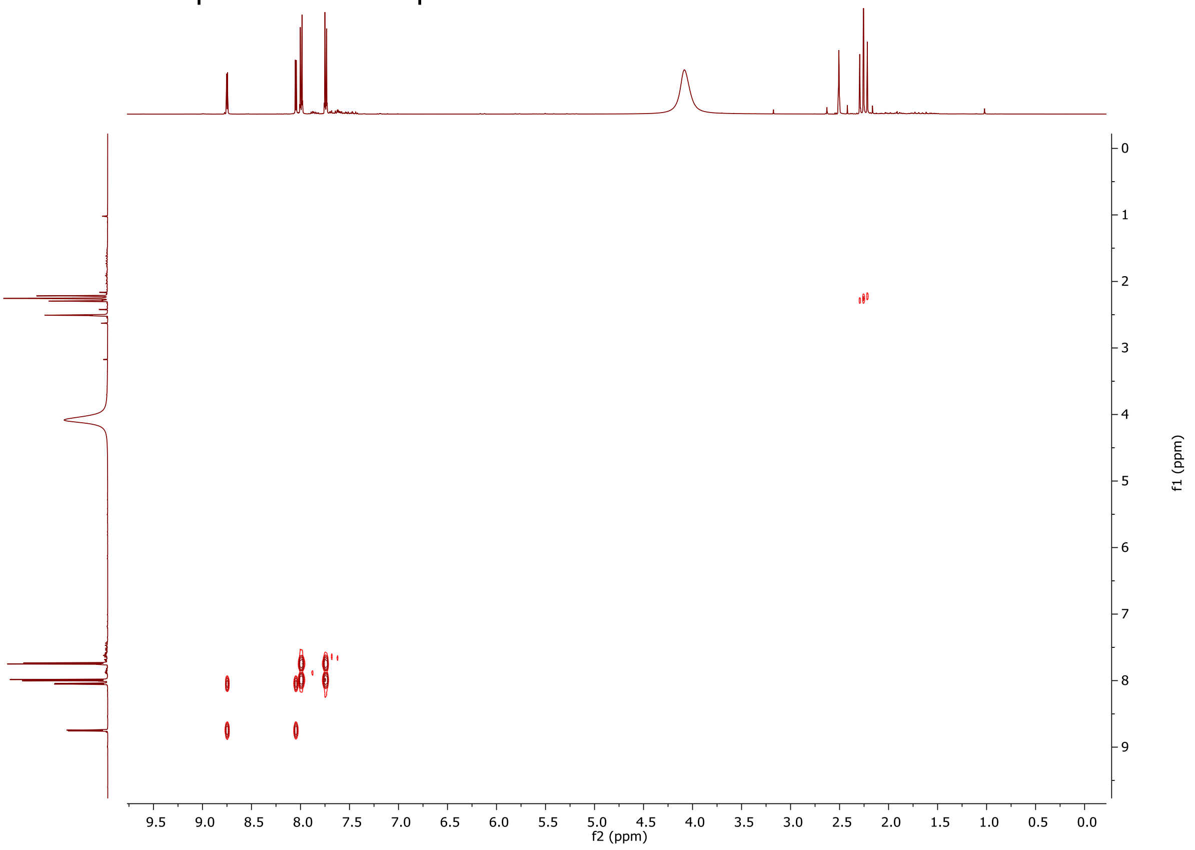

S94: HSQC spectrum of compound **16** in DMSO-d_6

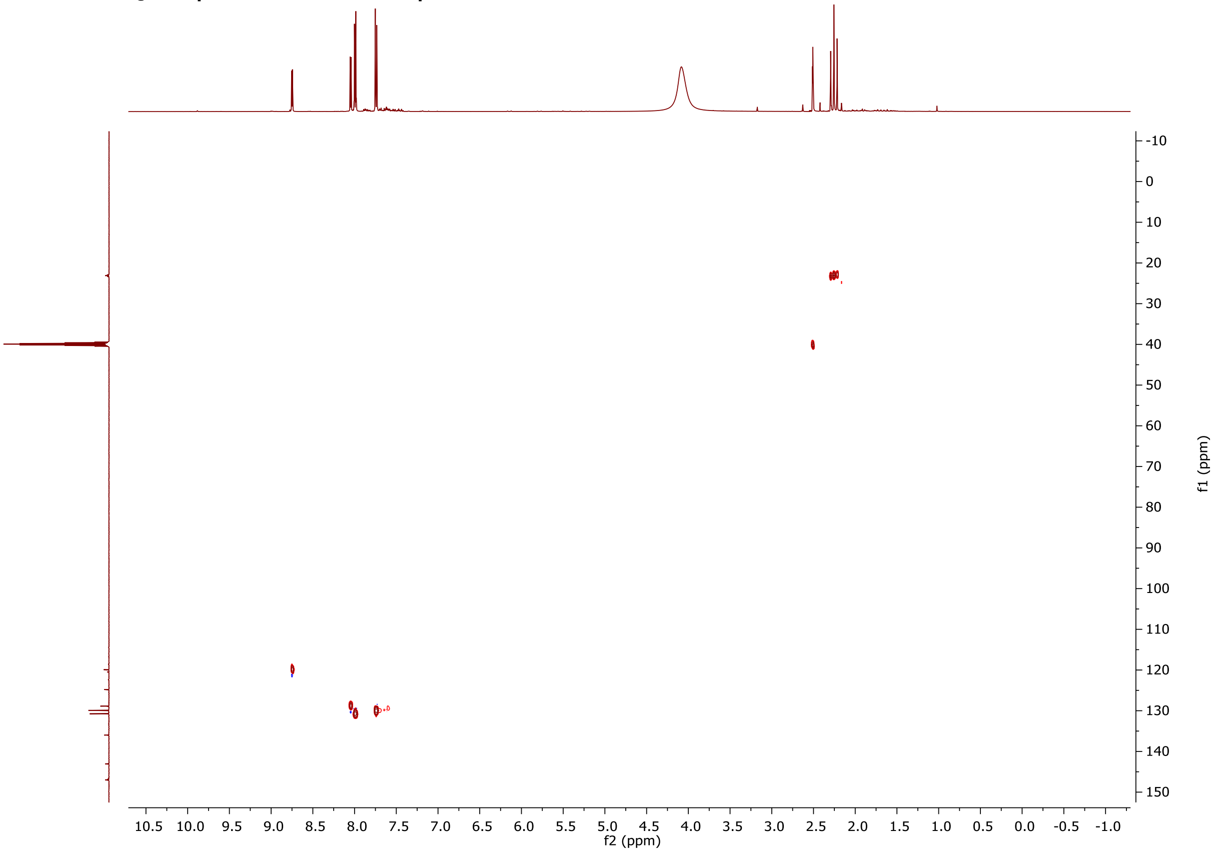

S95: HMBC spectrum of compound **16** in DMSO-d_6

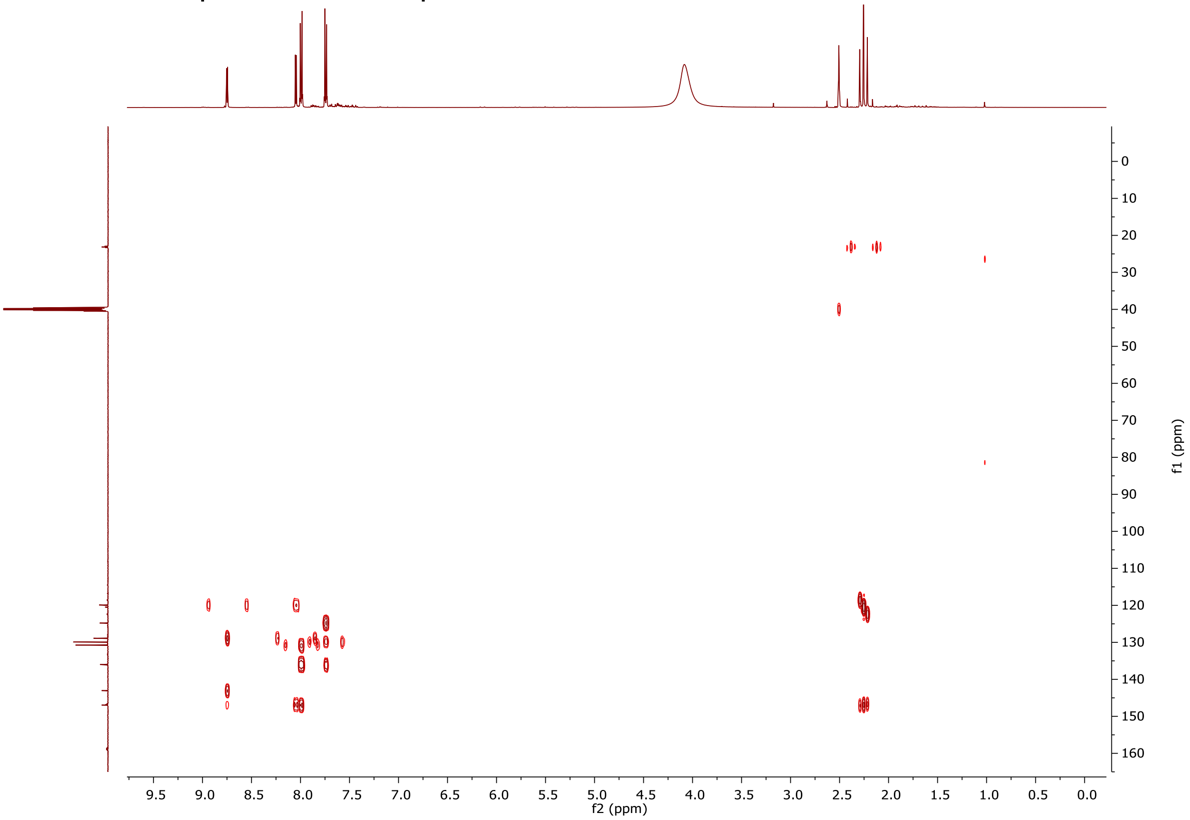

S96: HRMS of compound **16**

High Resolution Mass Spectrum

Analysis Info

Analysis Name	D:\Data\KahYean\20200904\RAD868000002.d		
Method	DirectInfusion_2018_pos.m	Acquisition Date	9/4/2020 12:05:33 PM
Sample Name	RAD868	Instrument	maXis II ETD 1823391.22321
Comment			

+MS, 0.3-0.3min #18-19

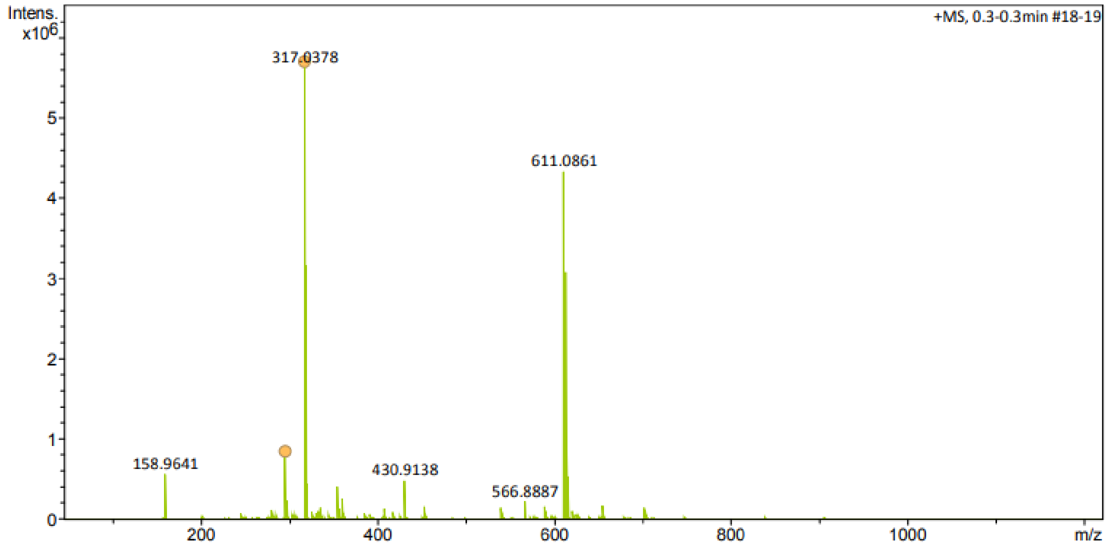

Meas. m/z	#	Ion Formula	m/z	err [ppm]	mSigma	# mSigma	Score	rdb	e⁻ Conf	N-Rule
295.0557	1	C13H10ClF2N4	295.0557	-0.1	11.0	1	100.00	9.5	even	ok
317.0378	1	C13H9ClF2N4Na	317.0376	-0.7	126.4	1	100.00	9.5	even	ok

S97: NMR data of compound **17** in DMSO-d_6[a]

Pos.	δ_H, mult. (*J* in Hz), int.	δ_C, mult. (*J* in Hz)	COSY	HMBC
3		147.1, s		
5		120.1, s		
6	7.99, s, 1H	128.5, s		5, 8, 9
8		156.3, s		
9		145.9, s		
10		126.3, s		
11	7.74, m, 1H	133.2, s	12	3, 13, 15
12	7.62, m, 1H	127.8, s	11	10, 14
13		135.4, s		
14	7.62, m, 1H	127.8, s	15	10, 12
15	7.74, m, 1H	133.2, s	14	3, 11, 13
18	3.75, m, 1H	38.5, s	19, 23	8, 20, 24
19	2.00, 2.14, m, 2H	26.7, d (9.6)	18, 20	18, 20, 21
20	2.08, 2.17, m, 2H	32.6, dd (23.8, 23.8)	19	18, 19, 21
21		123.9, dd (239.3, 241.6)		
22	2.08, 2.17, m, 2H	32.6, dd (23.8, 23.8)	23	18, 21, 23
23	2.00, 2.14, m, 2H	26.7, d (9.6)	18, 22	18, 21, 22

[a] Recorded at 500 MHz (^{1}H NMR) and 125 MHz (^{13}C NMR) at 25 °C.

S98: ^{1}H NMR Spectrum of compound **17** in DMSO-d_6

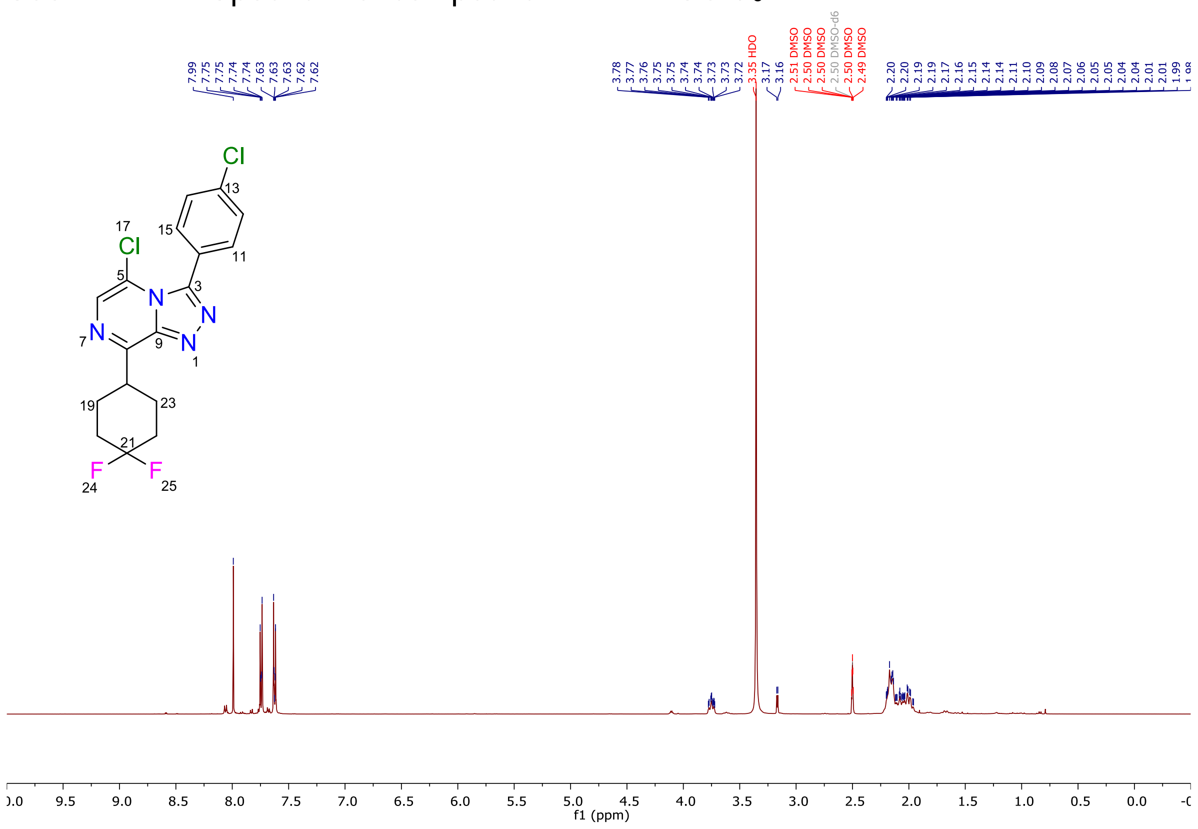

S99: ^{13}C NMR spectrum of compound **17** in DMSO-d_6

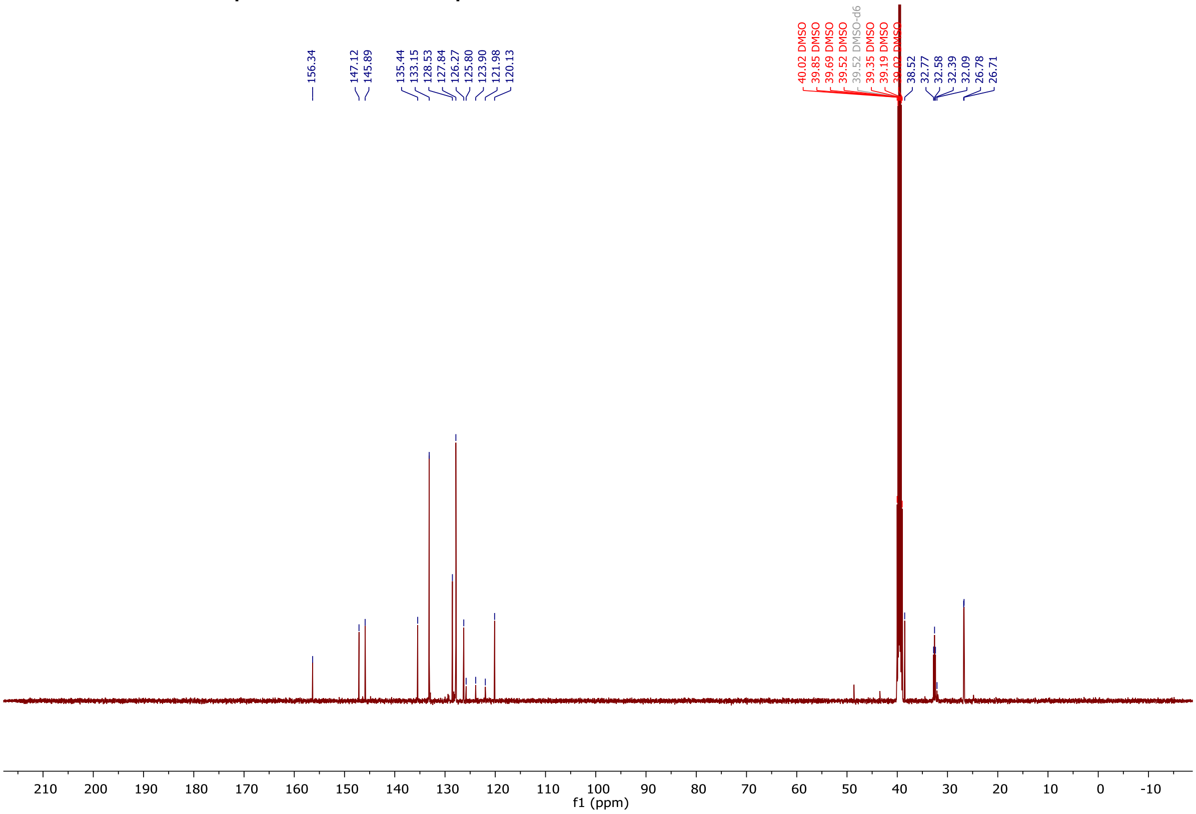

S100: COSY spectrum of compound **17** in DMSO-d_6

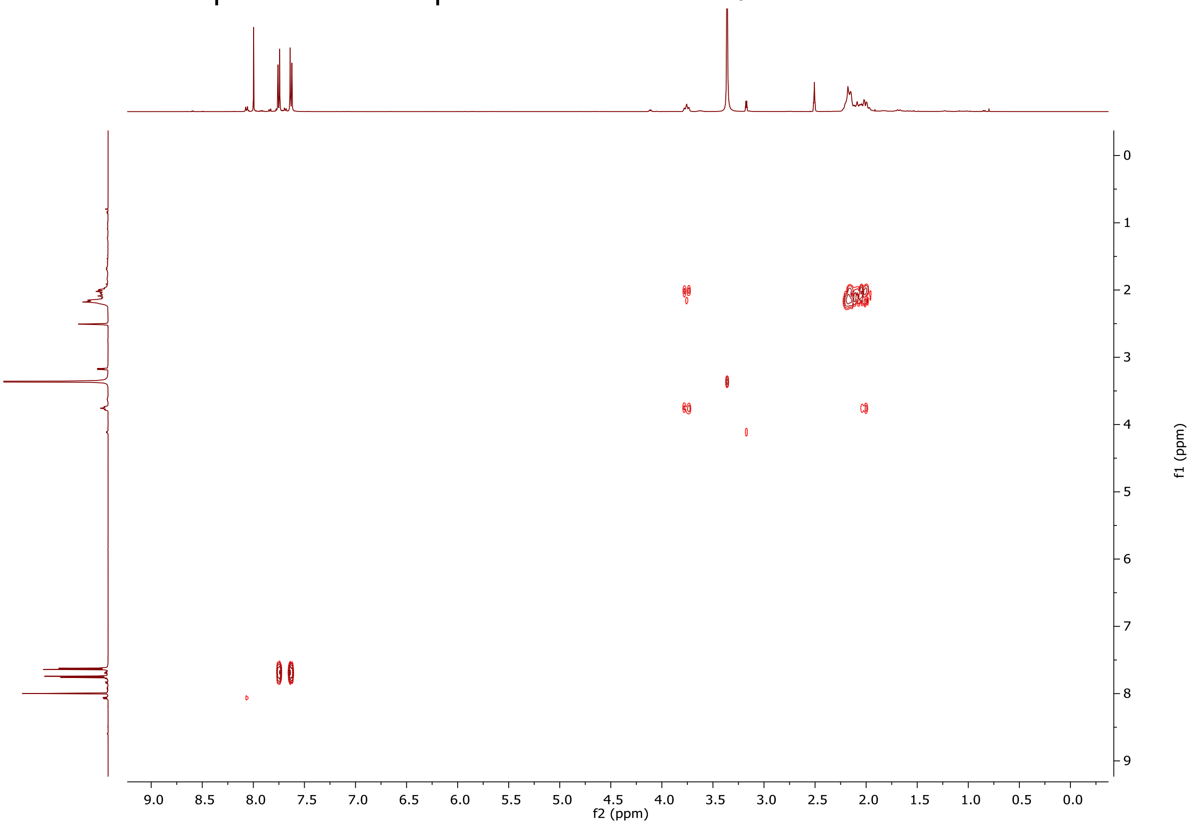

S101: HSQC spectrum of compound **17** in DMSO-d_6

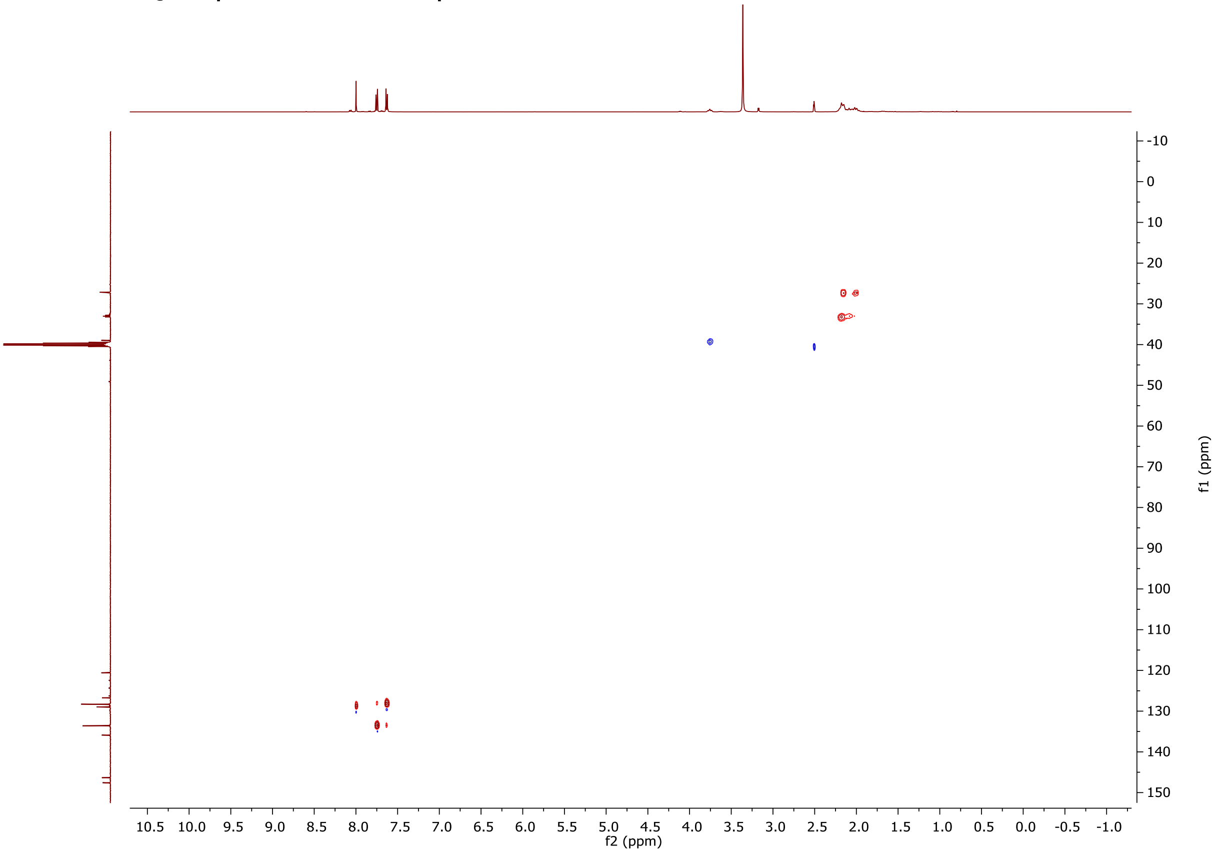

S102: HMBC spectrum of compound **17** in DMSO-d_6

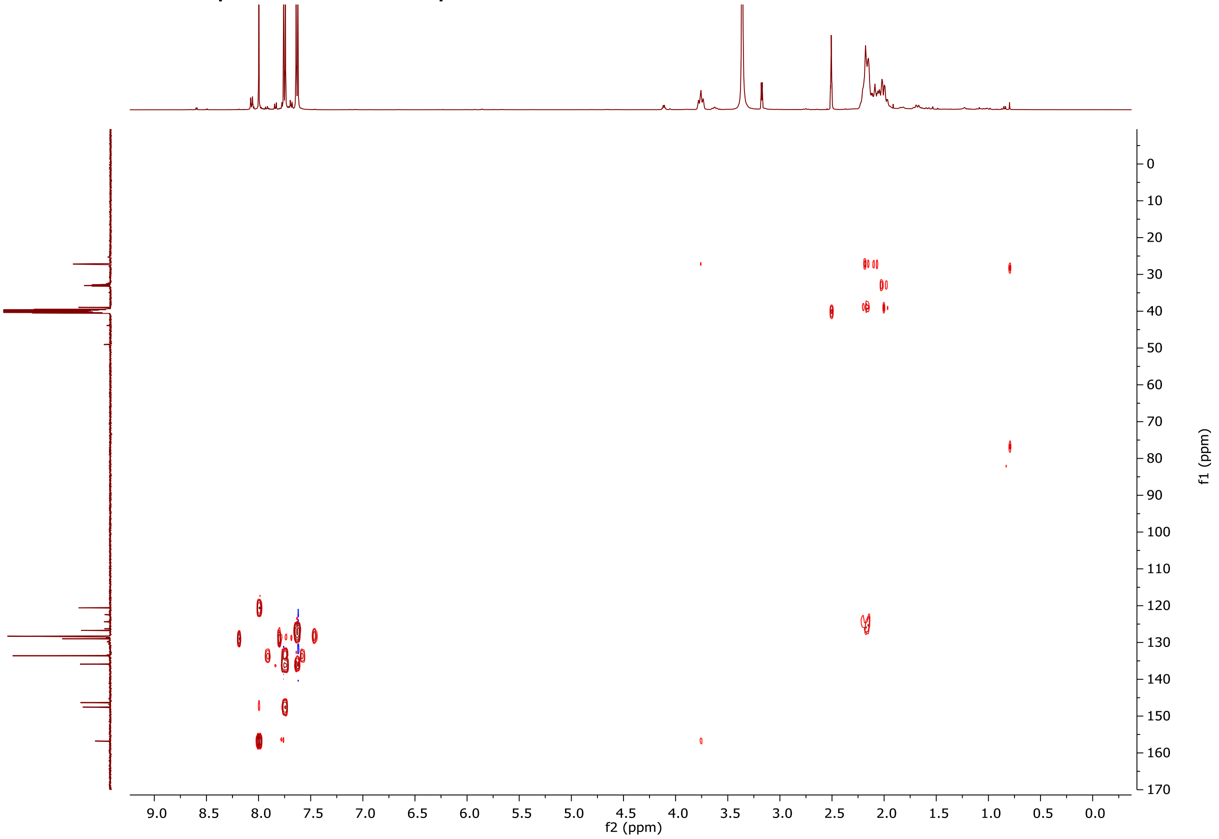

S103: HRMS of compound **17**

High Resolution Mass Spectrum

Analysis Info

Analysis Name D:\Data\KahYean\20200904\RAD869000001.d
Method DirectInfusion_2018_pos.m
Sample Name RAD869
Comment

Acquisition Date 9/4/2020 12:15:43 PM
Instrument maXis II ETD 1823391.22321

+MS, 0.5-0.5min #26-27

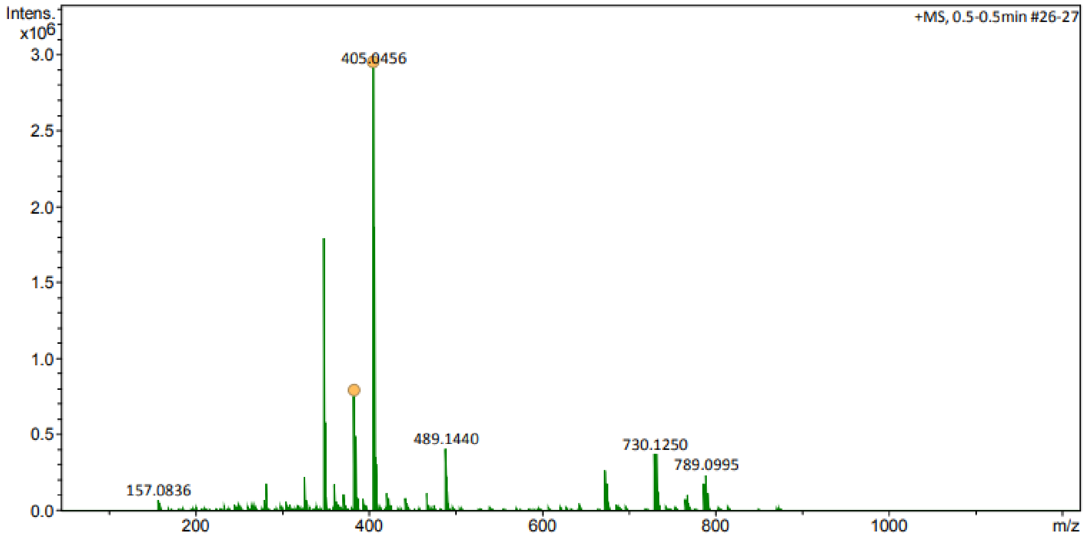

Meas. m/z	#	Ion Formula	m/z	err [ppm]	mSigma	# mSigma	Score	rdb	e⁻ Conf	N-Rule
383.0636	1	C17H15Cl2F2N4	383.0636	-0.0	7.7	1	100.00	10.5	even	ok
405.0456	1	C17H14Cl2F2N4Na	405.0456	-0.0	9.0	1	100.00	10.5	even	ok

S104: NMR data of compound **18** in DMSO-d_6[a]

Pos.	δ_H, mult. (*J* in Hz), int.	δ_C, mult. (*J* in Hz)	COSY	HMBC
3		146.4, s		
5	8.49, d (4.9), 1H	115.6, s	6	21
6	7.90, d (4.9), 1H	129.6, s	5	5, 8
8		157.6, s		
9		144.7, s		
10		124.7, s		
11	7.97, m, 1H	130.0, s	12	3, 13,15
12	7.71, m, 1H	129.4, s	11	10,14
13		135.3, s		
14	7.71, m, 1H	129.4, s	15	10, 12
15	7.97, m, 1H	130.0, s	14	3, 11, 13
17	3.75, m, 1H	38.9, s	18, 22	8, 18
18	2.03, 2.15, m, 2H	26.7, d (9.7)	17	17,19, 20, 23
19	2.06, 2.17, m, 2H	32.6, dd (24.5, 23.1)		17, 18, 20, 21
20		123.9, dd (239.5, 241.6)		
21	2.06, 2.17, m, 2H	32.6, dd (24.5, 23.1)		17, 19, 20, 22
22	2.03, 2.15, m, 2H	26.7, d (9.7)	17	17, 18, 20, 21

[a] Recorded at 500 MHz (^1H NMR) and 125 MHz (^{13}C NMR) at 25 °C.

S105: ^1H NMR spectrum of compound **18** in DMSO-d_6

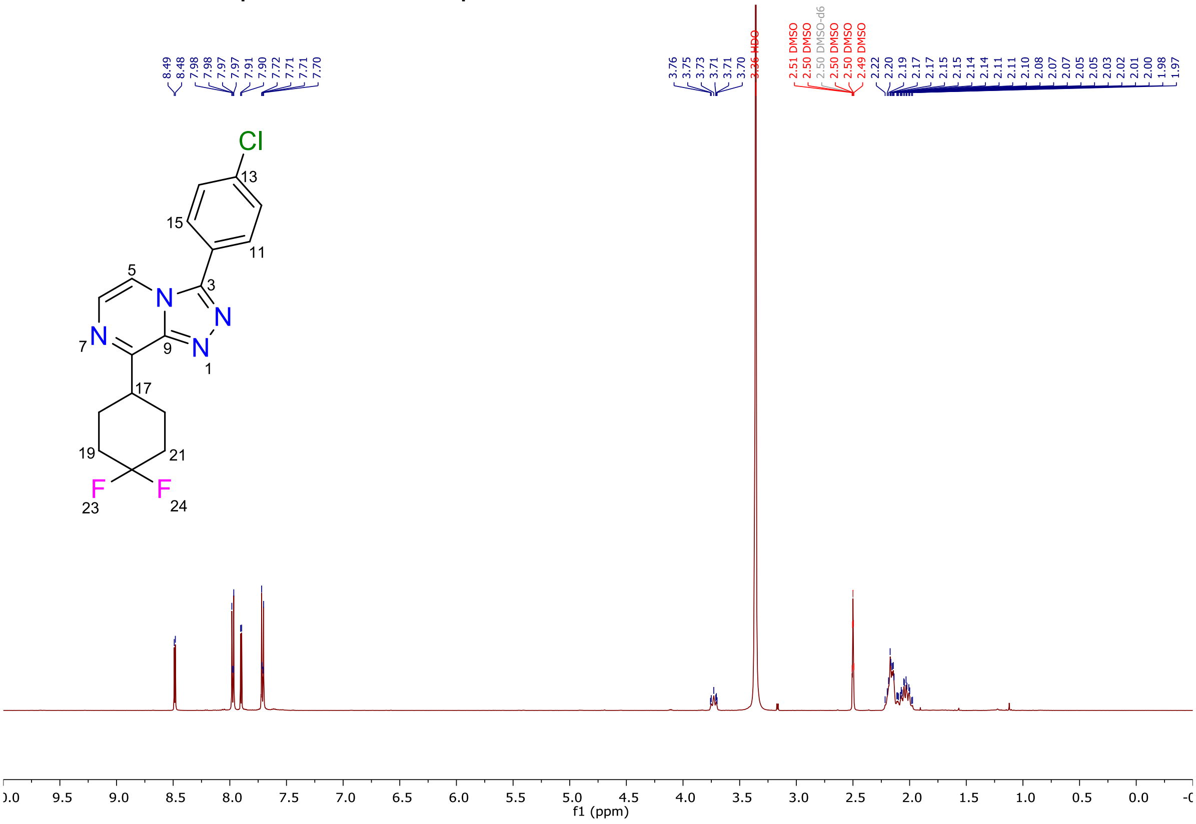

S106: ^{13}C NMR spectrum of compound **18** in DMSO-d_6

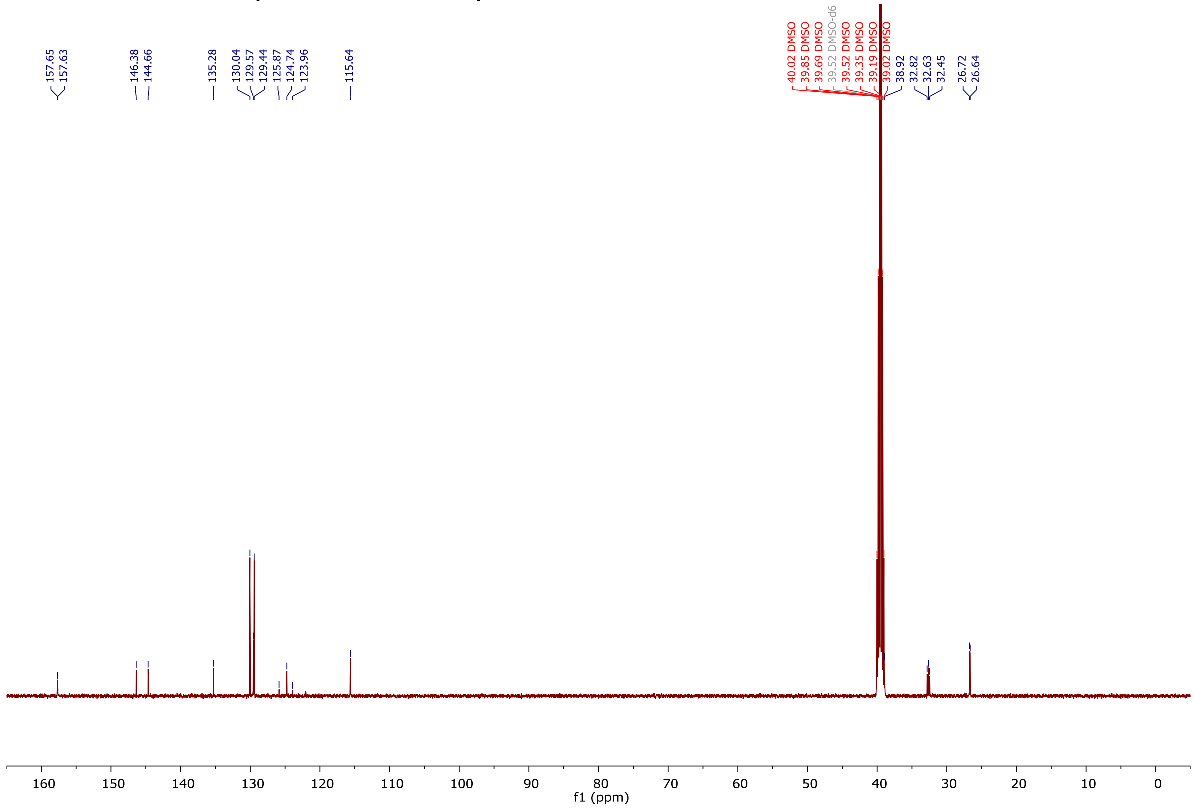

S107: COSY spectrum of compound **18** in DMSO-d_6

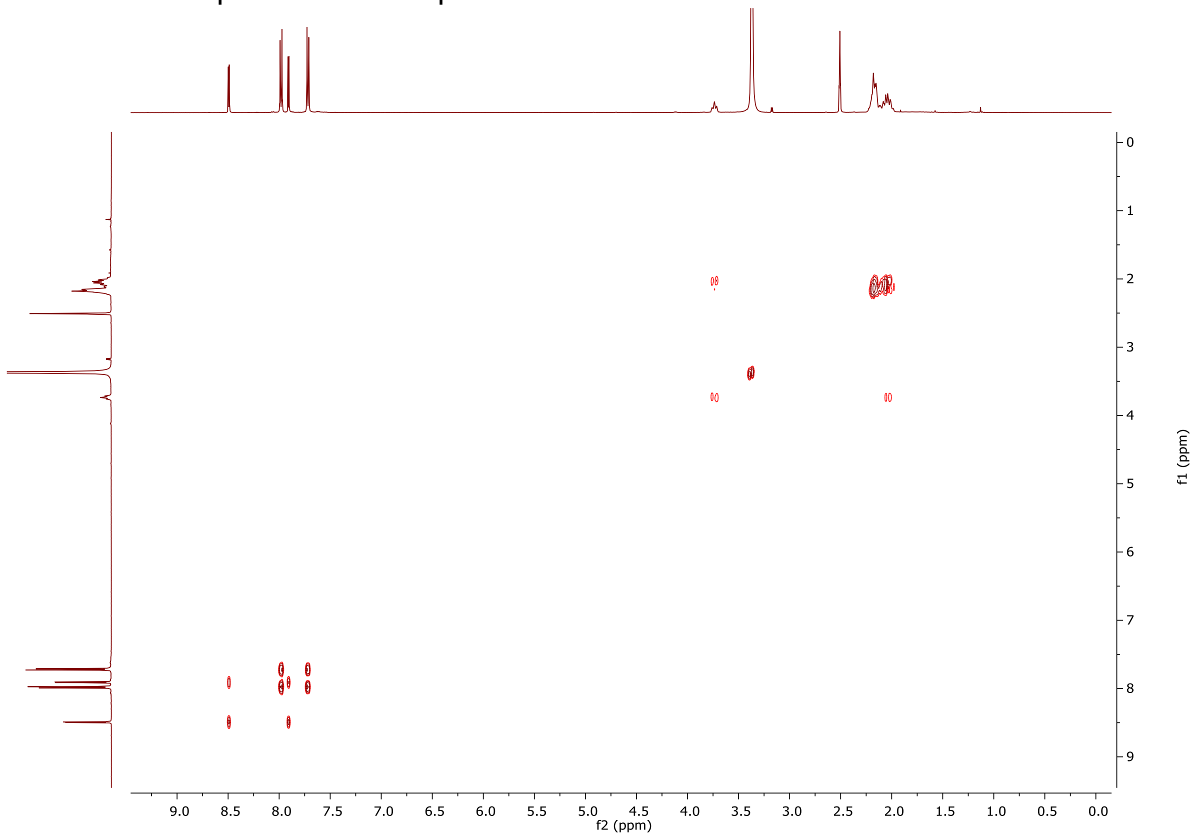

S108: HSQC spectrum of compound **18** in DMSO-d_6

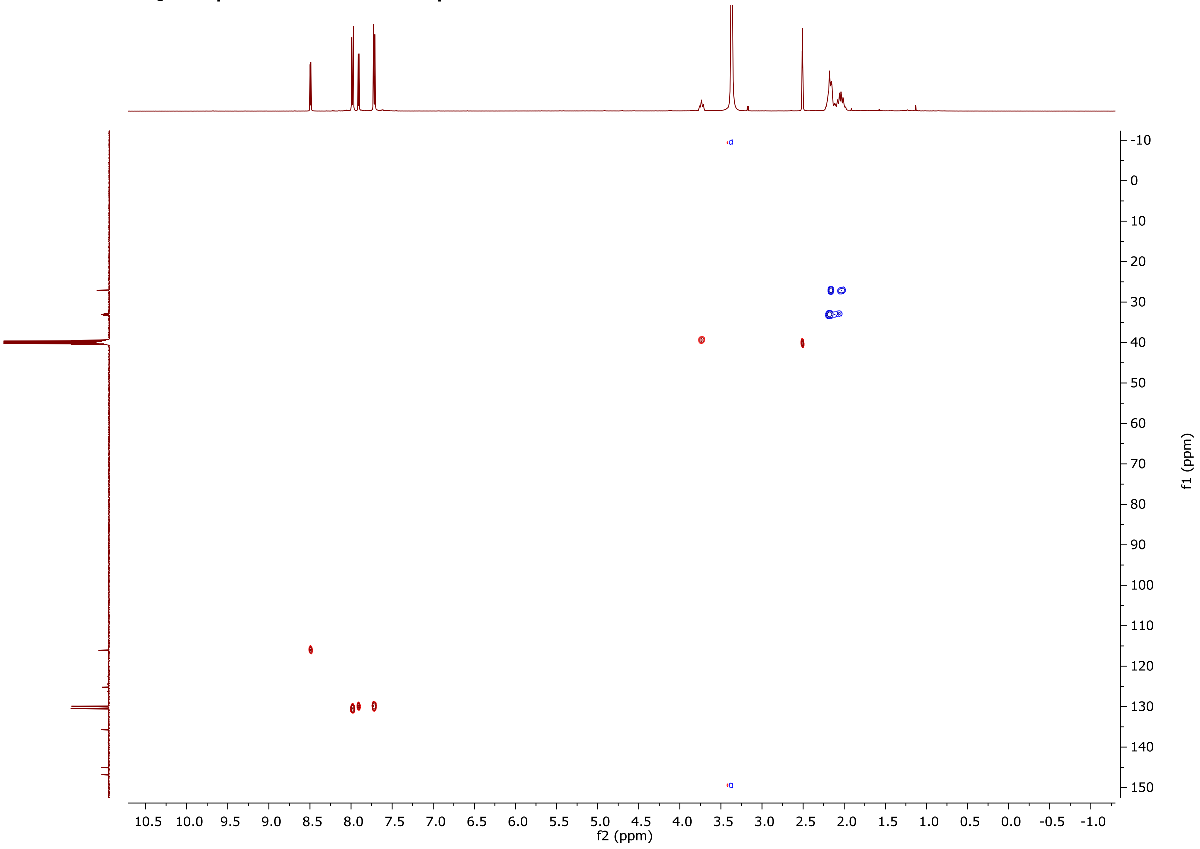

S109: HMBC spectrum of compound **18** in DMSO-d_6

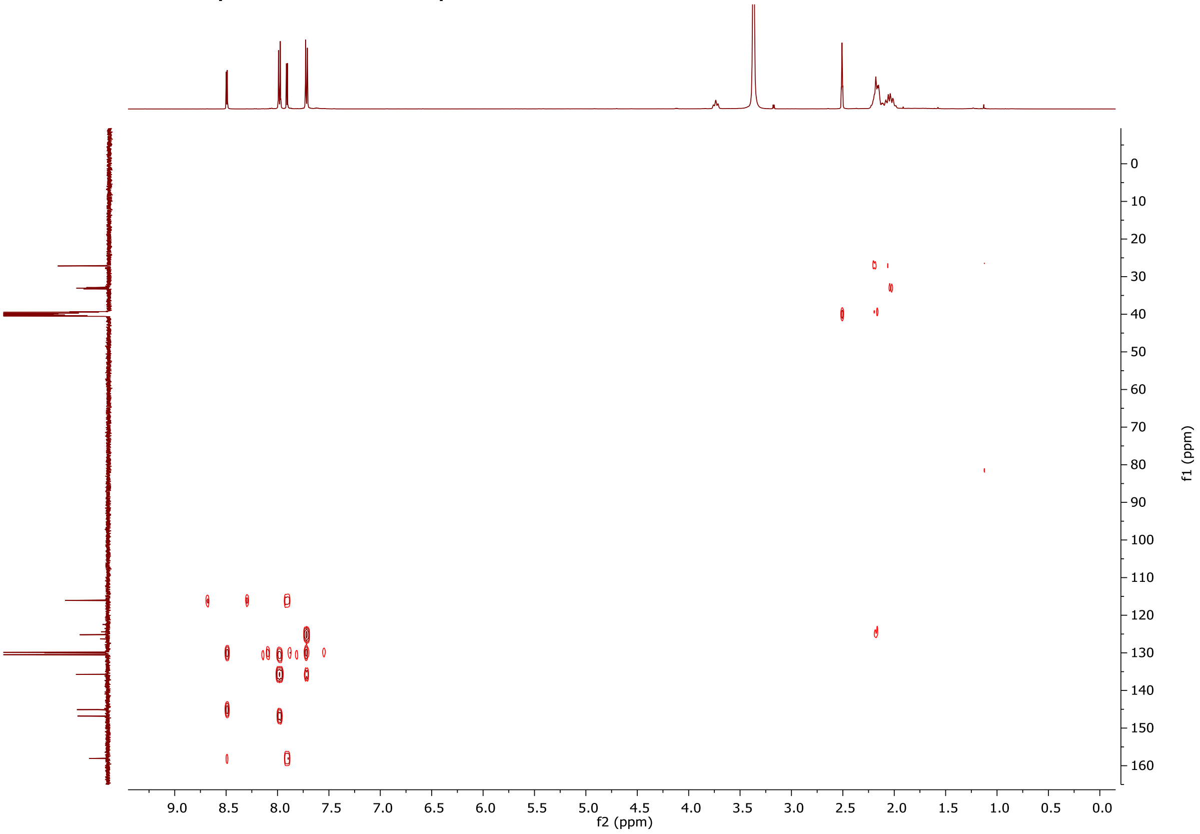

S110: HRMS of compound **18**

High Resolution Mass Spectrum

Analysis Info

Analysis Name	D:\Data\KahYean\20200904\RAD870000001.d		
Method	DirectInfusion_2018_pos.m	Acquisition Date	9/4/2020 12:22:44 PM
Sample Name	RAD870	Instrument	maXis II ETD 1823391.22321
Comment			

+MS, 0.2-0.2min #13-14

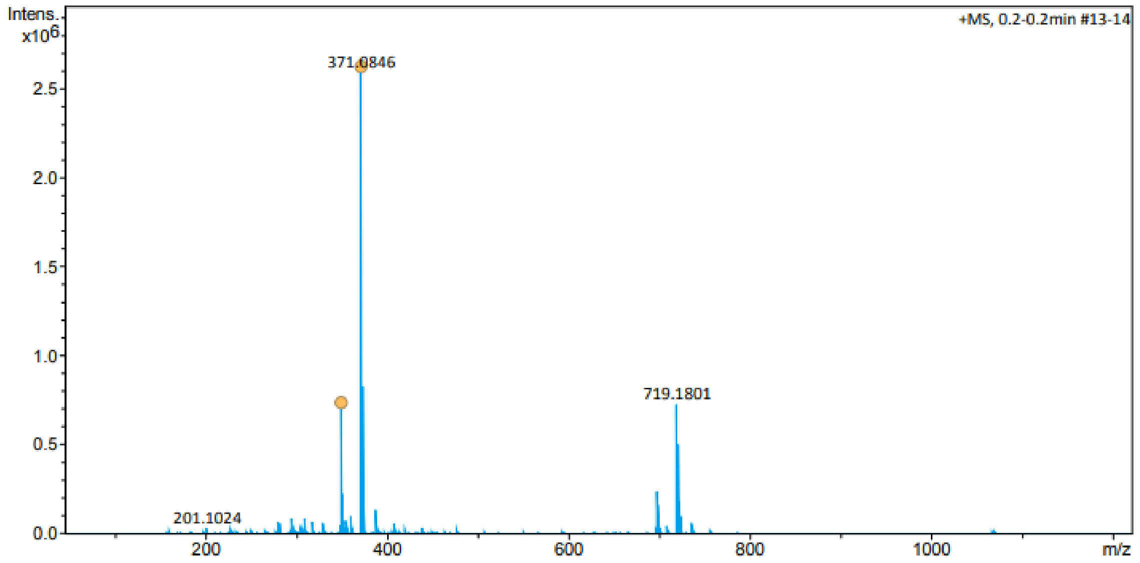

Meas. m/z	#	Ion Formula	m/z	err [ppm]	mSigma	# mSigma	Score	rdb	e⁻ Conf	N-Rule
349.1027	1	C17H16ClF2N4	349.1026	-0.2	11.4	1	100.00	10.5	even	ok
371.0846	1	C17H15ClF2N4Na	371.0846	-0.2	10.4	1	100.00	10.5	even	ok
